# Effect of Hydrogen Bonding and Chirality in Star-Shaped Molecules with Peripheral Triphenylamines: Liquid Crystal Semiconductors and Gels

**DOI:** 10.1021/acs.chemmater.3c03241

**Published:** 2024-05-02

**Authors:** Alejandro Martínez-Bueno, Santiago Martín, Josu Ortega, César L. Folcia, Roberto Termine, Attilio Golemme, Raquel Giménez, Teresa Sierra

**Affiliations:** † Departamento de Química Orgánica, Facultad de Ciencias, Universidad de Zaragoza, 50009 Zaragoza, Spain; ‡ Departamento de Química Física, Facultad de Ciencias, Universidad de Zaragoza, 50009 Zaragoza, Spain; § Laboratorio de Microscopias Avanzadas (LMA), Universidad de Zaragoza, 50018 Zaragoza, Spain; ∥ Department of Physics, Faculty of Science and Technology, Universidad del País Vasco UPV/EHU, 48080 Bilbao, Spain; ⊥ CNR-NANOTEC SS di Rende, Dipartimento di Fisica, Università della Calabria, 87036 Rende, Italy; ¶ Instituto de Nanociencia y Materiales de Aragón (INMA), CSIC-Universidad de Zaragoza, 50009 Zaragoza, Spain

## Abstract

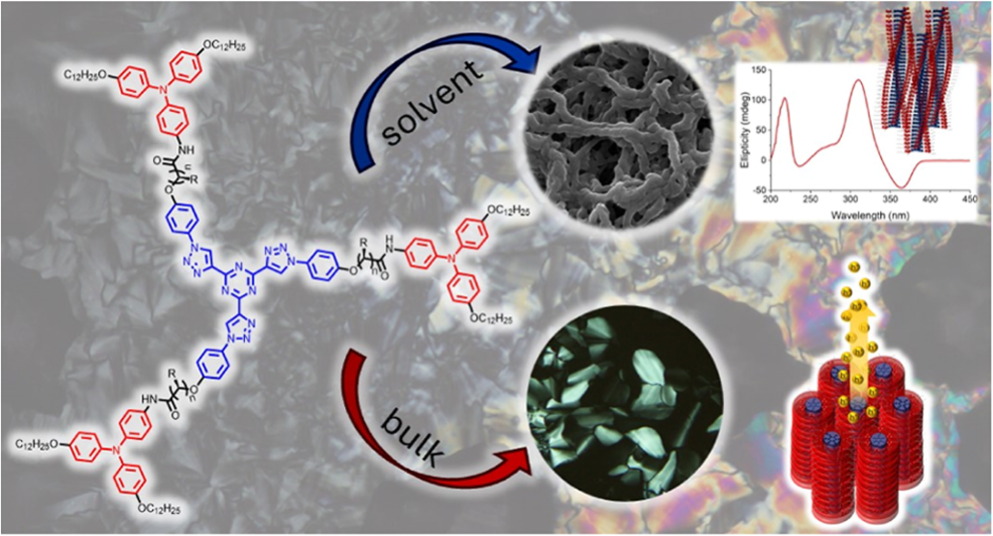

Organic semiconductors with well-defined architectures pose a suitable alternative to amorphous silicon-based inorganic semiconductors. Encouraged by the development of organic semiconductors based on columnar liquid crystals, herein, we report on a family of *C*_3_-symmetric star-shaped mesogens based on triphenylamine (TPA), a functional unit with strong electron donor character. Highly stable columnar phases with high hole mobility values were obtained out of this nonplanar functional unit, and this was achieved by using flexible amide spacers to join the TPA units to a tris(triazolyl)triazine (T) star-shaped core, allowing the formation of intermolecular hydrogen bonds. The presence of hydrogen bonds results in a stabilization of the columnar architectures either in bulk or in the presence of solvents by reinforcing π-stacking and van der Waals interactions, as deduced by Fourier-transform infrared (FTIR) and X-ray diffraction (XRD) studies. Furthermore, the introduction of a stereogenic center in the flexible spacer prompts the formation of chiral aggregates in the liquid crystal state and in the organogel formed in 1-octanol, as demonstrated by circular dichroism spectroscopy.

## Introduction

Advances in optoelectronic devices, thin-film transistors (FETs),^1^ light-emitting diodes (LEDs),^2^ or solar cells^3^ depend on the availability of high-performance semiconductors. In this respect, organic materials offer design and synthetic advantages, which include low-cost production, tunability, flexibility, and energetic efficiency.^4^ Furthermore, in contrast to the inorganic semiconductors, organic molecules are soluble in most common organic solvents which make them perfect candidates for the preparation of large-area thin films by solution processing techniques. However, there are still limitations to overcome, such as low charge carrier mobility because of orientational disorder and charge traps at grain boundary defects. In this context, columnar liquid crystals (CLCs) are good candidates to facilitate accurate solutions to those difficulties. First, they form anisotropic one-dimensional (1D) nanostructures by stacking of π-conjugated systems along which charge transport is favored,^5−7^ and their ability to dynamically respond to stimuli permits their processing into large-area oriented films.^8−12^ Like other macroscopic properties in CLCs, charge mobility is highly dependent on molecular arrangement, which in turn is dictated by intermolecular interactions and thus molecular design.

In order to obtain efficient devices with high charge mobility values and to establish a structure–property correlation, a large number of molecular architectures have been reported in recent years. Among them, planar cores such as triphenylene,^13−15^ perylene,^14,16,17^ or coronene^18−20^ are the most prominent due to their high propensity to form efficient stacks. In contrast, nonplanar propeller-shaped molecules, such as triphenylamine (TPA), with an exceptional electron-donating character, have been much less used in the design of semiconductor CLC. In fact, we described the first CLC based on TPA exhibiting high hole transport mobility, which consisted of a supramolecular mesogen formed by three TPA-containing benzoic acids hydrogen-bonded to a star-shaped *C*_3_-symmetric tris(triazolyl)triazine derivative. These complexes self-arranged into room-temperature hexagonal columnar (Col_h_) phase, albeit within a narrow temperature range.^21^ In addition to this example, only a few TPA-based CLCs have been described, all of them displaying a substitution at the para position of the three aromatic rings of a TPA core^22−27^ or multiple substitution with alkoxy chains,^28^ but no charge mobility was reported in any case. Interestingly, the presence of lateral amide groups in the TPA unit promoted the establishment of intermolecular hydrogen bonds, and this endowed this type of derivatives an alluring dual behavior^23^ by forming helical polymers with a columnar arrangement either in bulk or in the presence of solvents.^29,30^ Furthermore, taking advantage of the radical oxidation that TPA undergoes in chlorinated solvents,^28,31^ the assembly in supramolecular polymers can be controlled by light^32^ or electric fields,^33^ fostering the implementation of self-assembled hole-transporting nanowires.

**Scheme 1:**
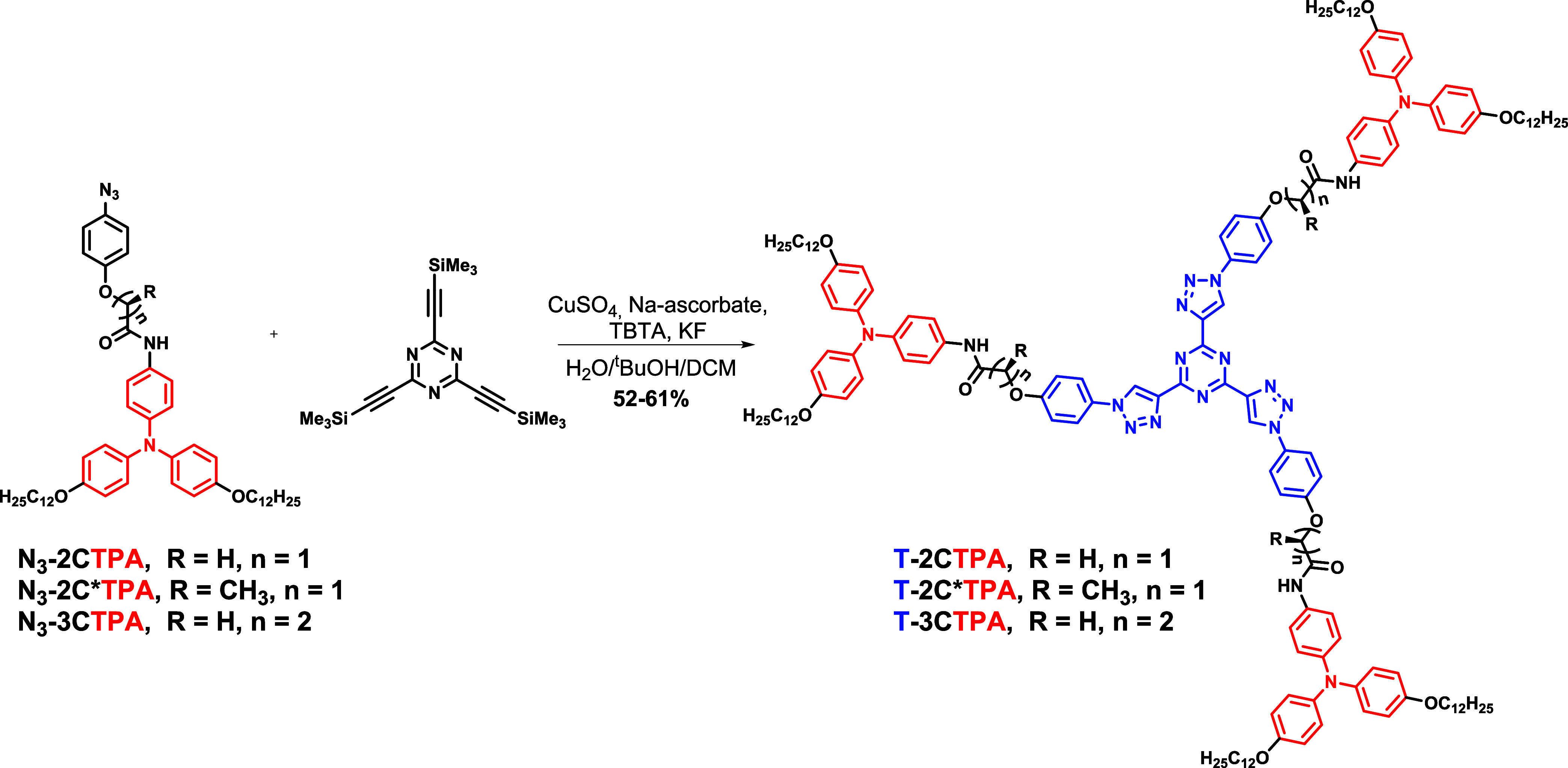
Synthesis of TPA-tris(triazolyl)triazine Derivatives

Based on this background, we set out to obtain TPA-containing functional materials, which exhibit stable columnar mesomorphism and dual behavior. To this end, we present here a new family of star-shaped *C*_3_-symmetric tricarboxamides with three peripheral TPAs attached via flexible amide-containing spacers to a tris(triazolyl)triazine (T) core (Scheme 1). The rationale of the mesogen design is based on a recent work in which we explored the inclusion of flexible amide spacers between the T core and three trialkoxyphenyl peripheral groups. We demonstrated that the hydrogen-bonding interactions established along the column promoted the formation of hexagonal columnar (Col_h_) mesophases at room temperature, BCC phases at high temperatures as well as the formation of columnar aggregates in the presence of 1-octanol.^34^ Here, and unexpectedly, the presence of nonplanar TPA moieties stabilizes the formation of columnar mesophases over a wide temperature range, whose properties are governed by the nature of the spacer, which dictates the formation of intermolecular hydrogen bonds. Moreover, TPA endows the liquid crystal materials with semiconducting properties obtaining charge mobility values in the order of 10^–2^ cm^2^ V^–1^ s^–1^. Additionally, like their trialkoxyphenyl analogues, these materials gel 1-octanol forming Col_h_ arrays without disturbing intermolecular hydrogen bonds, and in some cases also gel 1,4-dioxane and heptane. The presence of the chiral spacer allowed us to propose the formation of helical organizations in which the chirality is transferred from the molecule to the columnar stacks, both in the mesophase and in the gels.

## Results and Discussion

### Synthesis

Star-shaped compounds were synthesized by a triple one-pot copper-catalyzed azide–alkyne cycloaddition^35^ between 2,4,6-tris[(trimethylsilyl)ethynyl]-1,3,5-triazine and the aromatic azides with a TPA moiety, in a mixture of water, *tert*-butanol, and dichloromethane, following a modified synthetic protocol previously described by our group (Scheme 1).^34^ To avoid alkyne side-polymerization, the ethynyl groups were deprotected *in situ* by adding an aqueous solution of KF to the reaction mixture.^36^

**Scheme 2:**
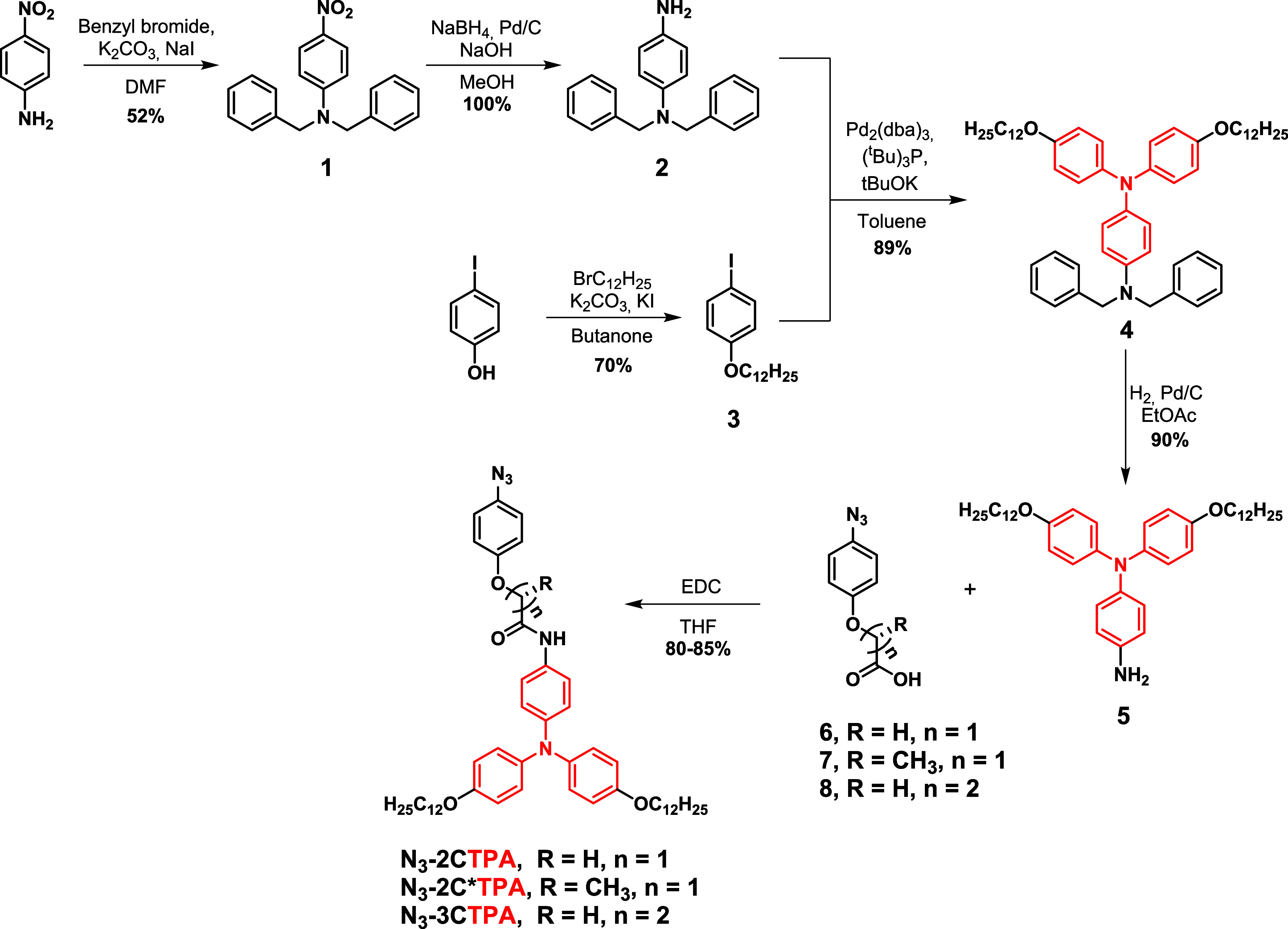
Synthesis of Aromatic Azides with TPA Moiety

The aromatic azides with a TPA moiety (**N**_**3**_**-2CTPA**, **N**_**3**_-2C*TPA, and **N**_**3**_**-3CTPA**) were synthesized through an amidation reaction between the TPA moiety functionalized with an amine group **5**, and azide containing carboxylic acids **6**, **7**, and **8** described previously,^34^ as depicted in Scheme 2.

The synthesis of TPA moiety **5** was previously reported using two different strategies. The first one consists of a modified Ullman reaction between *p*-nitroaniline and 1-alkyloxy-4-iodobenzene and subsequent reduction of the nitro group using tin chloride.^31^ The second one uses p-nitrofluorobenzene in a bis(4-alkyloxyphenyl)amine previously synthesized through a Buchwald–Hartwig C–N coupling and subsequent reduction of the nitro group using hydrazine in the presence of Pd/C.^33^ Despite the first strategy being more direct than the second, long reaction times are needed to get good yields. In this work, we use a different strategy which consists of a Buchwald–Hartwig C–N coupling of *N*^1^,*N*^1^-bis(phenylmethyl)-1,4-benzenediamine, **2**, with *p*-dodecyloxyiodobenzene (**3**). The reaction takes place overnight with high yields. For this purpose, *p*-nitroaniline was protected with two benzyl groups and the nitro group was quantitatively reduced in 30 min with sodium borohydride in the presence of Pd/C^37^ to yield the aniline **2**. Subsequently, the aniline **2** was coupled with **3** giving the TPA protected amine **4**, which was debenzylated by hydrogenolysis to yield **5**.

### Liquid Crystal and Thermal Properties

The thermal properties and liquid crystal behavior of the three compounds, **T-2CTPA**, T-2C*TPA, and **T-3CTPA**, were studied by polarized optical microscopy (POM), thermogravimetric analysis (TGA), differential scanning calorimetry (DSC), and X-ray diffraction (XRD). Thermal data are summarized in Table 1.

**Table 1:** Thermal Properties of the Corresponding Columnar Mesophases

compound	thermal properties^a^ (*T* °C, [Δ*H* kJ/mol])	*T*_5%_^b^
**T-2CTPA**	Col_h(g)_ 117 Col_h_ 273 [3.0] I^c^	335
T-2C*TPA	Col_h_ 152 [1.3] I^d^	338
**T-3CTPA**	Col_h_ 165 [16.3] Col_tet_ 244 [4.4] I^c^	319

a Col_h(g)_: glassy hexagonal columnar mesophase, Col_h_: hexagonal columnar mesophase, I: isotropic liquid, Col_tet_: tetragonal columnar mesophase.

b Temperature corresponding to a 5% weight loss by thermogravimetry.

c Thermal data obtained from the second heating process at a rate of 20 °C/min.

d Thermal data obtained from the second heating process at a rate of 2 °C/min.

**Figure 1:**
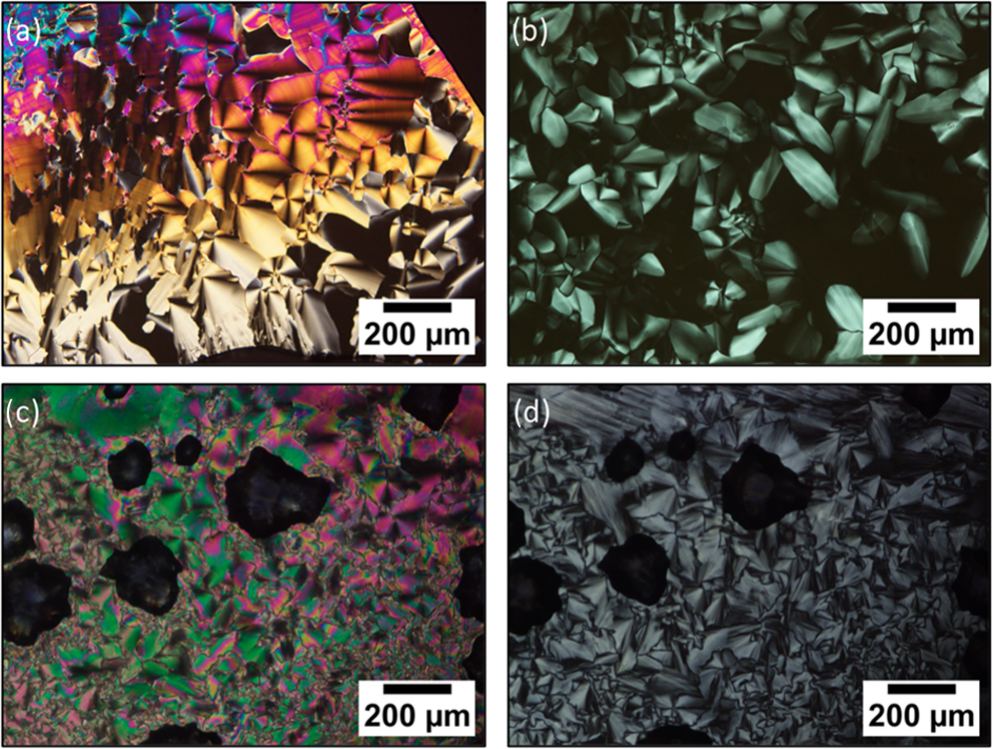
Photomicrographs of textures observed by POM for (a) **T-2CTPA** at room temperature, (b) T-2C*TPA at room temperature, (c) **T-3CTPA** at room temperature, (d) **T-3CTPA** at 180 °C on cooling from the isotropic liquid.

Birefringent focal conic fan-shaped textures observed by POM revealed that all of the compounds exhibit columnar mesomorphism on cooling from the isotropic liquid (Figure 1). Furthermore, for the compound with the longest spacer, **T-3CTPA**, two mesophases appeared during the cooling process, observed as a drastic color change of the texture (Figure 1c,d).

**Figure 2:**
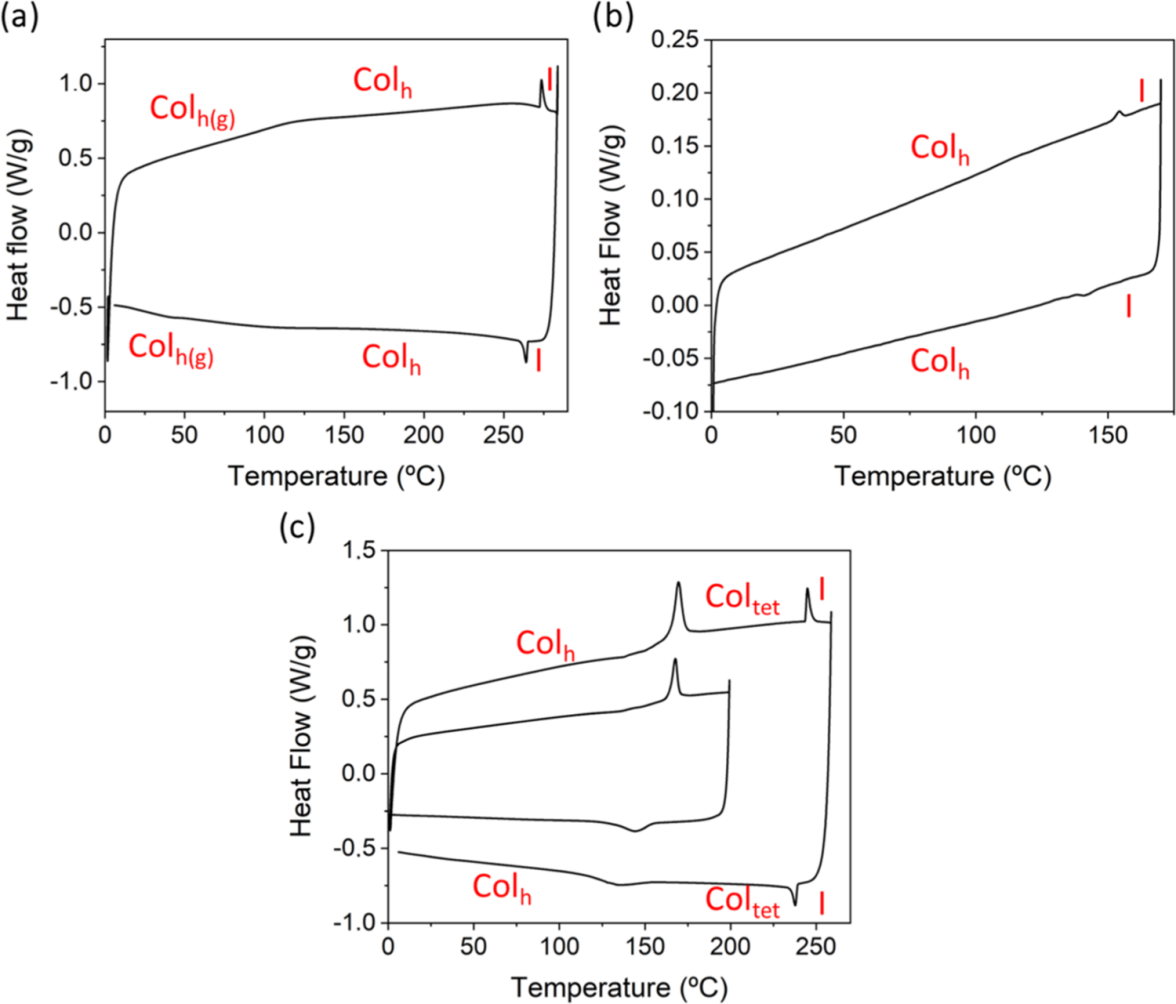
DSC thermograms of compounds (a) **T-2CTPA**, (b) T-2C*TPA, and (c) **T-3CTPA**. The thermograms were recorded at a rate of 10 °C/min, for **T-2CTPA** and **T-3CTPA**, and 2 °C/min for T-2C*TPA.

In addition to POM observations, DSC analysis evidenced that all of the three compounds display liquid crystalline properties in a broad range of temperatures. On the heating process, compound **T-2CTPA** (Figures 2a and S9) shows a glass transition at 117 °C and a peak with onset at 273 °C corresponding to the transition to the isotropic liquid. On cooling, the formation of the mesophase and the glass transition appear at 265 °C and at 105 °C, respectively. The thermogram of the chiral analogue T-2C*TPA (Figures 2b and S10) shows the transition from the mesophase to the isotropic liquid as a peak with onset at 152 °C, during the heating process, and the mesophase formation at 146 °C, during the cooling cycle. In this case, the mesophase formation was observed by POM as a slow process, and therefore the DSC scans were performed at 2 °C/min in order to see correctly the peak associated with the appearance of the mesophase.

In contrast to their analogues, the thermogram of compound **T-3CTPA** (Figures 2c and S11) shows two transitions, the first one at 165 °C and the second one at 244 °C, the last one corresponding to the transition to the isotropic liquid. The first transition coincides with the color variation of the texture observed by POM, and it is due to a mesophase change as explained by XRD studies. Both transitions can be also observed in the cooling cycle.

Columnar mesomorphism was confirmed by X-ray diffraction (XRD) experiments measured at room temperature in samples cooled from the isotropic liquid (from 160 °C for T-2C*TPA, or from 200 °C for **T-2CTPA** and **T-3CTPA**). The XRD data are summarized in Table 2.

**Table 2:** X-ray Diffraction Data

compound	*T* (°C)	mesophase	lattice parameters	*d*_obs_ (Å)	*d*_calc_ (Å)	Miller indices (*hkl*)
**T-2CTPA**	r.t.	**Col**_**h**_	*a* = 49.5 Å	42.9	42.9	(100)
			*c* = 3.4 Å	24.7	24.8	(110)
				21.5	21.5	(200)
				16.2	16.2	(210)
				4.5 (dif)		
				3.4		(001)
T-2C*TPA	r.t.	**Col**_**h**_	*a* = 40.6 Å	35.2	35.2	(100)
				4.5 (dif)		
**T-3CTPA**	r.t.	**Col**_**h**_	*a* = 47.5 Å	41.1	41.1	(100)
				23.9	23.7	(110)
				20.6	20.6	(200)
				15.5	15.5	(210)
				4.5 (dif)		
	180 °C	**Col**_**tet**_	*a* = 44.8 Å	44.8	44.8	(100)
				31.8	31.7	(110)
				15	14.9	(300)
				4.5 (dif)		

**Figure 3:**
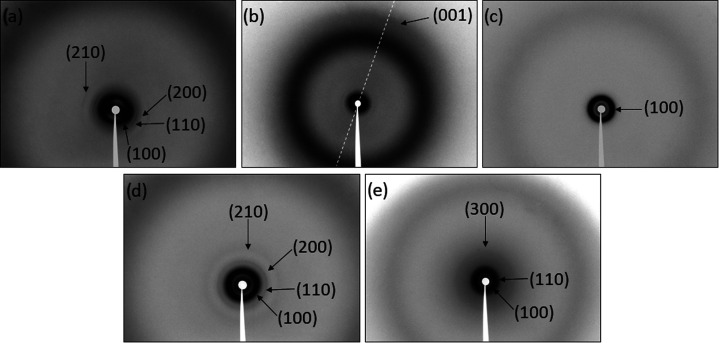
X-ray diffractograms for the compounds: (a) **T-2CTPA**, r.t., (b) **T-2CTPA** partially aligned sample along the capillary axis (dashed line), r.t., (c) T-2C*TPA, r.t., (d) **T-3CTPA**, rt, and (e) **T-3CTPA**, 170 °C.

At room temperature, the diffractograms of compounds **T-2CTPA** and **T-3CTPA** show four reflections at the small angle region related to distances with a ratio *d*, *d*/√3, *d*/√4 and *d*/√7, which correspond to the respective indices (100), (110), (200) and (210) of a hexagonal lattice (Figures 3a,b,d and SI12a,c), with lattice parameters 49.5 and 47.5 Å, respectively. The diffractogram of the chiral compound T-2C*TPA shows only one reflection at low angles (Figures 3c and SI12b), which does not allow to confirm unambiguously the hexagonal symmetry of the mesophase. However, based on the texture observed by POM (Figure 1b) and previous reports for other tris(triazolyl)triazine structures with a discotic shape and similar liquid crystal behavior,^35,38^ the formation of a Col_h_ mesophase with a lattice parameter 40.6 Å is proposed. For all of the three compounds, a diffuse (dif) broad reflection is observed at 4.5 Å due to the liquid-like arrangement of the alkyl chains, confirming the liquid crystalline behavior. For compound **T-2CTPA**, an outer diffuse halo at 3.4 Å is observed, and reinforced along the alignment direction when the material is partially aligned along the capillary axis (Figure 3b) and corresponds to a periodic intracolumnar distance. In order to estimate the number of molecules per unit cell (*Z*), density measurements were carried out by the buoyancy method (see the SI). For compounds **T-2CTPA** and **T-3CTPA**, density values of 1.1 g/cm^3^ were obtained while for the chiral compound T-2C*TPA a density of 1 g/cm^3^ was measured. Considering the cell parameter (*a*) of the hexagonal network and assuming a π-stacking distance (*c*) of 3.4 Å, as observed in the XRD pattern of **T-2CTPA**, the number of molecules per unit cell is *Z* = 2 for compounds **T-2CTPA** and **T-3CTPA**, and Z = 1 for compound T-2C*TPA, which is in agreement with its much smaller lattice parameter.

The result of *Z* = 2 for **T-2CTPA** and **T-3CTPA** is in contrast with analogous trisamide tris(triazolyl)triazine compounds derived from trialkoxyaniline and a total of nine terminal chains, where the Col_h_ phase was formed by one molecule per unit cell.^34^ Nevertheless, it aligns with the value of *Z* = 2 found in the columnar mesophases of tris(triazolyl)triazine derivatives with six peripheral chains,^35,39^ which is possible due to the low energy difference between the *C*_3_ and asymmetric conformations of the tris(triazolyl)triazine core.^40^**T-2CTPA** and **T-3CTPA** possess a low number of peripheral tails, precisely six, as substituents in nonplanar TPA units, and their disposition is rather unusual, which might not inherently promote efficient space filling. Accordingly, it is reasonable to propose the arrangement of two molecules in a non-C_3_-symmetric conformation creating a disk-like entity surrounded by 12 alkyl tails in a similar manner to what was previously described.^35,39^ This, in turn, facilitates the formation of columns, favoring the Col_h_ phase with higher space-filling efficiency across a broad temperature range. Unlike **T-2CTPA** and **T-3CTPA**, the chiral derivative T-2C*TPA shows a much smaller lattice parameter and behaves as an individual stacking unit (*Z* = 1). It is evident that the branched spacer leads to higher steric demand that would favor the star-shaped *C*_3_ conformation of T-2C*TPA, making it less prone to form dimers and allowing the stacking of individual molecules, likely in a helical disposition as deduced from CD spectra (see below).

Compound **T-3CTPA** was also studied above 170 °C to get information about the second mesophase observed by POM and DSC. Above this temperature, the XRD diffraction pattern is significantly different and presents three reflections at small angles, with spacings at a ratio *d*, *d*/√2, and *d*/√9 (Figures 3e and SI12d). These reflections are related with the indices (100), (110), and (300) of a tetragonal lattice with a parameter *a* = 44.8 Å.^27,41^

### Hydrogen Bond Studies by FTIR

**Figure 4:**
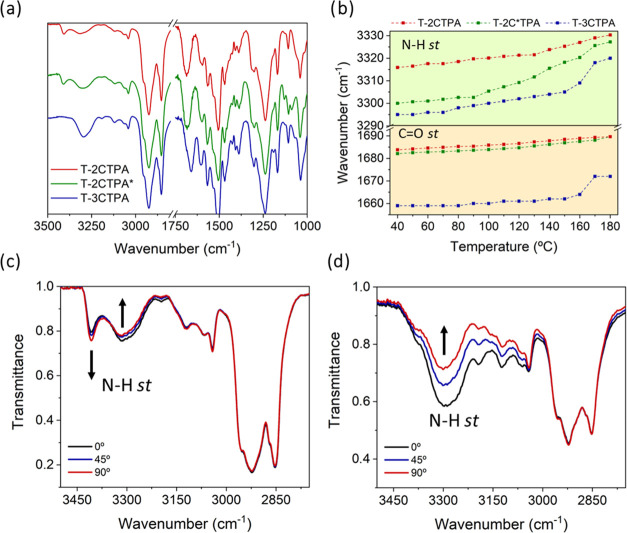
(a) FTIR spectra of **T-2CTPA** (red), T-2C*TPA (green), and **T-3CTPA** (blue) at room temperature; (b) spectral shifts of the hydrogen-bonded N–H st band (green) and C=O st band (yellow) during the heating process; (c) polarized FTIR spectra (the 0° of the polarizer were made to coincide with horizontally oriented columns) for **T-2CTPA**, (d) for **T-3CTPA**.

Intracolumnar hydrogen bonds established between amides were studied by FTIR at variable temperatures and by polarized FTIR on mechanically aligned samples (Figures 4 and SI13–15). At room temperature, the two compounds with the shortest spacer (**T-2CTPA** and T-2C*TPA) show two N–H *st* bands around 3300 and 3400 cm^–1^, which correspond to hydrogen-bonded and free N–H bonds, respectively. Also, the C=O *st* band comes out as a broad band between 1655 and 1715 cm^–1^, which agrees with overlapped hydrogen-bonded and free C=O bands. The presence of both types of bands, namely, associated and nonassociated, indicate a partial involvement of the amide groups in intermolecular hydrogen bonds. However, the intensity ratio between the hydrogen-bonded and the free N–H *st* bands is higher for T-2C*TPA compared to **T-2CTPA**, and this can be tentatively related with the different stacking structures proposed above. In contrast, compound **T-3CTPA** shows only one N–H *st* band at 3295 cm^–1^ and a sharp C=O *st* band at 1660 cm^–1^, corresponding to fully hydrogen-bonded N–H and C=O groups, which could be accounted for by the higher flexibility of the 3C spacer that facilitates amide associations.

When compounds **T-2CTPA** and T-2C*TPA were heated, the intensity of the hydrogen-bonded N–H *st* band gradually decreased and shifted to higher wavenumbers, whereas the intensity of the free N–H *st* band increased (Figures 4b, SI13, and SI14). The C=O *st* band also shifted to higher wavenumbers as expected because of hydrogen bonding weakening with increasing temperature. Compound **T-3CTPA** showed a similar behavior as the temperature increased (Figures 4b and S15). However, coinciding with the transition from the Col_h_ to the Col_tet_ mesophase at 170 °C, both N–H and C=O *st* bands exhibited an abrupt shift to higher wavenumbers and a drastic decrease of intensity. Moreover, a shoulder at 3410 cm^–1^ corresponding to the free N–H *st* band appeared, and this indicates that the Col_h_ to Col_tet_ transition occurs along with partial cleavage of intramolecular hydrogen bonds.

In order to investigate the directionality of the intermolecular hydrogen bonds, polarized FTIR studies were conducted for mechanically aligned samples. Successful alignment was achieved exclusively for compounds **T-2CTPA** and **T-3CTPA** by shearing the materials between two KBr plates at 200 °C, as confirmed by POM (Figures SI16 and SI17). However, attempts to align at various temperatures, compound T-2C*TPA did not show successful alignment. The polarized FTIR spectra of both compounds revealed noticeable intensity changes in the main bands when the polarizer was rotated (Figure 4c,d). These intensity changes were more pronounced for **T-3CTPA** (Figure 4d) compared to **T-2CTPA** (Figure 4c), which could be attributed to a significant presence of non-hydrogen-bonded N–H groups in the latter, as mentioned earlier. The most meaningful variations were observed in the N–H *st* band, which displayed the highest intensity when the polarizer is parallel to the shearing direction, and therefore aligned with the columnar direction. This observation is consistent with the formation of intermolecular hydrogen bonds involving amide groups mainly along the direction of the columns. Furthermore, the intensity of C_ar_-H *st* bands decreased upon rotation of the polarizer to a position orthogonal to the column axis. This suggests a preferred alignment of certain C_ar_-H bonds along the columnar axis, and this must be related with aromatic rings lying out of the plane of the tris(triazolyl)triazine core. Indeed, this deviation has already been determined by theoretical calculations for the phenyl groups linked to the triazole rings,^40^ as well as for TPA units surrounding a hydrogen-bonded disk-like structure with a tris(triazolyl)triazine core.^21^

### Chiroptical Properties of the Mesophase

**Figure 5:**
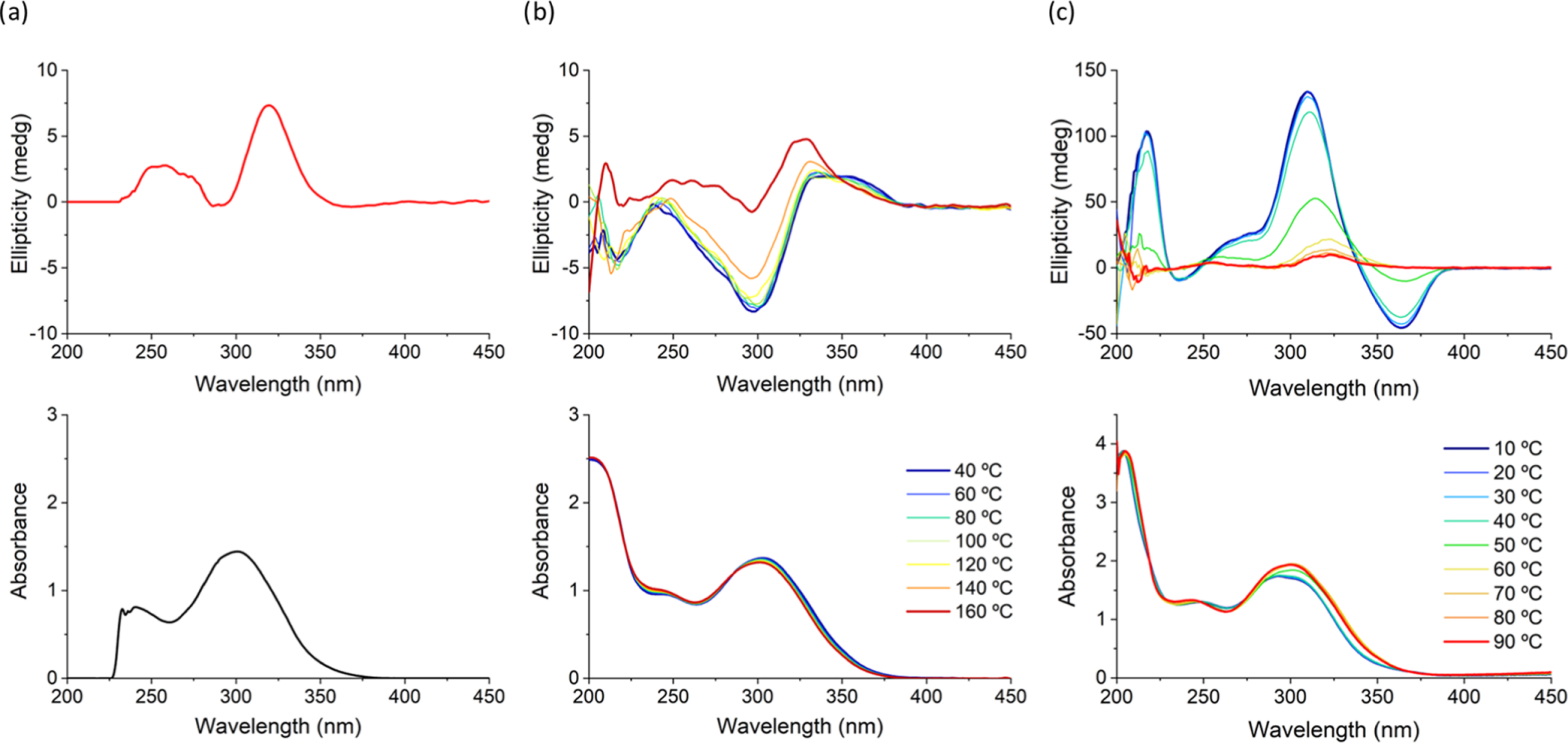
Circular dichroism (top) and UV/vis spectra (down) recorded for T-2C*TPA in (a) THF solution (10^–5^ M), (b) thin film at different temperatures, and (c) 0.5 wt % gel in 1-octanol.

Electronic circular dichroism (CD) and ultraviolet–visible (UV–vis) spectra of T-2C*TPA were recorded for a 10^–5^ M THF solution (Figure 5a) and for a thin film at variable temperature (Figure 5b) to study molecular chirality and to verify its transference to the columnar assembly in the mesophase.

T-2C*TPA exhibits CD activity in both dilute solution in THF (10^–5^ M) and the mesophase, although the corresponding spectra are different. The CD spectrum of the THF solution displays two positive bands at 319 nm and around 255 nm, which lie within the UV–vis absorption region of the two chromophores (Figure 5a). It is interesting to note at this point that the azide precursor, **N**_**3**_-2C*TPA, does not show optical activity at the same concentration (Figure SI20). This indicates that the stereogenic center in the spacer does not cause a detectable chiral perturbation into the TPA chromophore, preventing **N**_**3**_-2C*TPA from being CD active. As a result, it can be postulated that the star-like shape of the molecule restricts the relative conformational freedom of both the core and the arms. This characteristic serves as a first level of transmission of chirality from the spacer to the molecule, making it well-suited for a chiral self-assembly process. A thin film of T-2C*TPA at room temperature exhibits a notably different CD spectrum (Figure 5b, blue line), characterized by two negative CD bands at 210 and 297 nm, corresponding to both chromophores, as confirmed by the UV–vis spectra of the molecule and of its individual chromophores (Figure SI21).^28^ Additionally, a positive band prominently appears at 355 nm, which can be exclusively associated with TPA units in a chiral environment. These observations suggest that the self-assembly of T-2C*TPA in the columnar phase represents a second level of chiral transmission, with the molecules stacked in a chiral manner. CD spectra recorded at different temperatures further support the conveyance of molecular chirality to the columnar organization. Above the transition to the isotropic liquid, the CD spectrum displays the signals observed for the molecularly dissolved T-2C*TPA indicating that the bands at 160 °C are solely attributable to molecular chirality. Upon cooling just below the transition from the isotropic liquid, the CD spectrum recovers the profile observed at room temperature, and this confirms that the optical activity of T-2C*TPA in the mesophase results from a helical arrangement of molecular chromophores along the column.

### Charge Transport Properties

The electrochemical properties of the new compounds were studied by cyclic voltamperometry for determining the redox potentials and the HOMO and LUMO levels in solution (Table SI1) and in film (Table 3), with the aim of establishing the feasibility of the materials for electron- and hole-injection processes.

**Table 3:** Electrochemical Data of the Compounds **T-2CTPA**, T-2C*TPA, and **T-3CTPA** in Thin Film

compound	*E*^red^ (V) vs Ag/AgCl	*E*_1/2_^ox^ (V) vs Ag/AgCl	*E*^red^ (V)^a^ vs FOC	*E*_1/2_^ox^ (V)^a^ vs FOC	HOMO (eV)^b^	LUMO (eV)^c^
**T-2CTPA**	–1.77	0.76	–2.20	0.33	–5.13	–2.60
T-2C*TPA	–1.75	0.77	–2.18	0.31	–5.14	–2.62
**T-3CTPA**	–1.71	0.75	–2.14	0.32	–5.12	–2.66

a *E*_1/2_ = 0.43 V vs Ag/AgCl.

b *E*_HOMO_ = −e[*E*_1/2_^ox^ vs FOC + 4.8 V].

c *E*_LUMO_ = −e[*E*^red^ vs FOC + 4.8 V].

All three compounds show similar cyclic voltamperograms with a reversible oxidation wave at 0.75–0.77 V corresponding to the oxidation process of the triphenylamine units to the radical cation^42^ (Table 3), and a reversible reduction wave between −1.71 and −1.77 V due to the presence of the tris(triazolyl)triazine core (Figures SI22 and SI23).^39^ According to this, the HOMO and LUMO values were estimated around −5.1 and −2.6 eV, respectively, which are consistent with the electron donor character of the TPA units and the electron-acceptor character of the core.^21^

**Figure 6:**
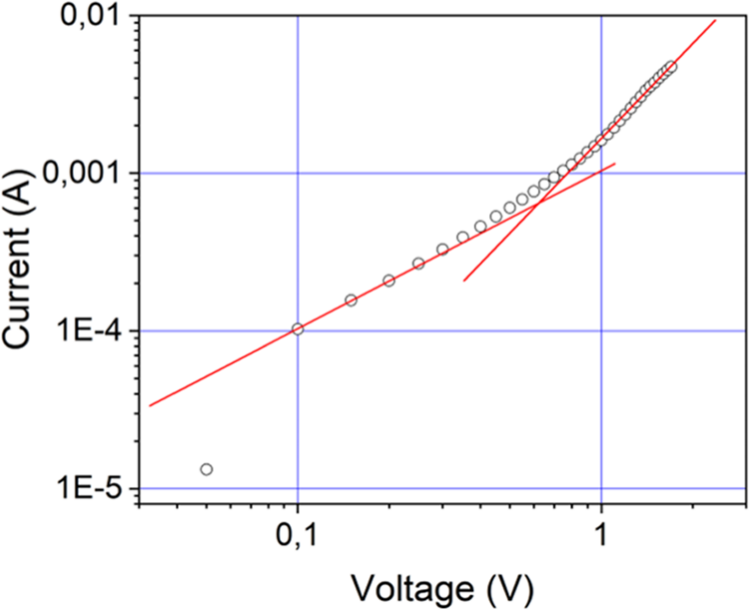
Typical *J*–*V* curve observed for compound T-2C*TPA at rt.

The charge transport properties of the materials were studied via space charge limited current (SCLC) method in solution-processed thin films (all of the experimental details are given in the SI).^20,43^ According to the HOMO energy level of the materials (*E*_HOMO_ ≈ −5.13 eV), Au was selected as injecting electrode (*W*_Au_ ≈ −5.1 eV) in order to get an effective ohmic contact. Indium Tin Oxide (ITO) was selected as counter electrode due to its work function (*W*_ITO_ ≈ −4.7 eV), which is considerably lower than the LUMO energy level of the materials (*E*_LUMO_ ≈ −2.62) eV. All of the three compounds showed the typical current/voltage curves with linear to quadratic transitions around 1 V (Figure 6).

SCLC measurements are sensitive to column orientation since charges must travel between both electrodes. In order to optimize charge mobility, thermal treatments were performed on all three samples to enhance column alignment perpendicular to the substrate. The specific thermal treatment of each sample was tailored based on their thermal properties. Thus, T-2C*TPA was annealed at 20 °C below the clearing point for 4 h, while compounds **T-2CTPA** and **T-3CTPA** were annealed at 200 °C for 1 h to prevent decomposition. Subsequently, all three compounds were slowly cooled down to room temperature. After the thermal treatment, T-2C*TPA displayed no birefringence between crossed polarizers, indicating a homeotropic alignment of the columns, which is favorable for precise charge mobility measurements. Indeed, consistent high mobility values across the sample, in the range 10^–2^–10^–3^ cm^2^ s^–1^ V^–1^, were measured (Figure SI24b). On the other hand, **T-2CTPA** and **T-3CTPA** exhibited low birefringent textures with small domains (Figure SI25). The trend of the hole mobility values was similar for both materials. Some areas of the samples showed high mobility values (10^–2^–10^–3^ cm^2^ s^–1^ V^–1^), while others showed medium-low values (10^–5^–10^–8^ cm^2^ s^–1^ V^–1^) (Figure SI24a,c). These results highlight the well-established strong correlation between the measured charge mobility and the orientational order in the mesophase, in terms of both the orientation of columns relative to the electrode surfaces and the size of the orientational domains.

As the tris(triazolyl)triazine core has electron-acceptor character (*E*_LUMO_ ≈ – 2.6 eV),^15^ electron mobility cells were prepared by using CsCO_3_ coated ITO (*W* ≈ −2.9–3.1 eV)^44^ as injecting electrode and Al (*W*_Al_ ≈ 4.2 eV) as counter electrode. Unfortunately, in this case, the typical current/voltage curves were not observed, and the obtained currents were in the order of 10^–9^ A.

### Gelation Properties

Aligned with our interest in discovering versatile molecules for crafting functional materials in various environments, such as bulk and solution, we further investigated the potential of these three molecules to serve as components of soft self-assembled materials fabricated in solvents. Indeed, preliminary NMR experiments at different concentrations indicate that even in CDCl_3_, which is a good solvent for the three compounds, they all exhibit upfield shifting of protons of the tristriazolyltriazine core. This phenomenon arises from mutual shielding of the aromatic rings, evidencing aggregation through pi-stacking (Figure SI26),^40,45^ which is further enhanced by H-bonds between amide groups, inferred from the downfield shift of the NH proton. Consequently, the feasibility of preparing gel materials through the self-aggregation of these molecules was investigated using different organic solvents. Table 4 collects the results observed at a concentration of 1 wt %. All three compounds are capable of gelling 1-octanol and forming opaque gels stable at room temperature. Additionally, **T-3CTPA** was found to gel other types of solvents such as heptane and dioxane.

**Table 4:** Gelation Test^a^ Results in Organic Solvents at 1 wt %, and Gel-Sol Transition Temperatures (in Parentheses)

compound	heptane	dodecane	1-octanol	cyclohexane	1,4-dioxane	toluene
**T-2CTPA**	S	S	G (85 °C)	S	P	S
T-2C*TPA	S	S	G (60 °C)	S	S	S
**T-3CTPA**	G (85 °C)	S	G (60 °C)	S	G (50 °C)	P

a S = solution, G = gel, P = precipitate

**Figure 7:**
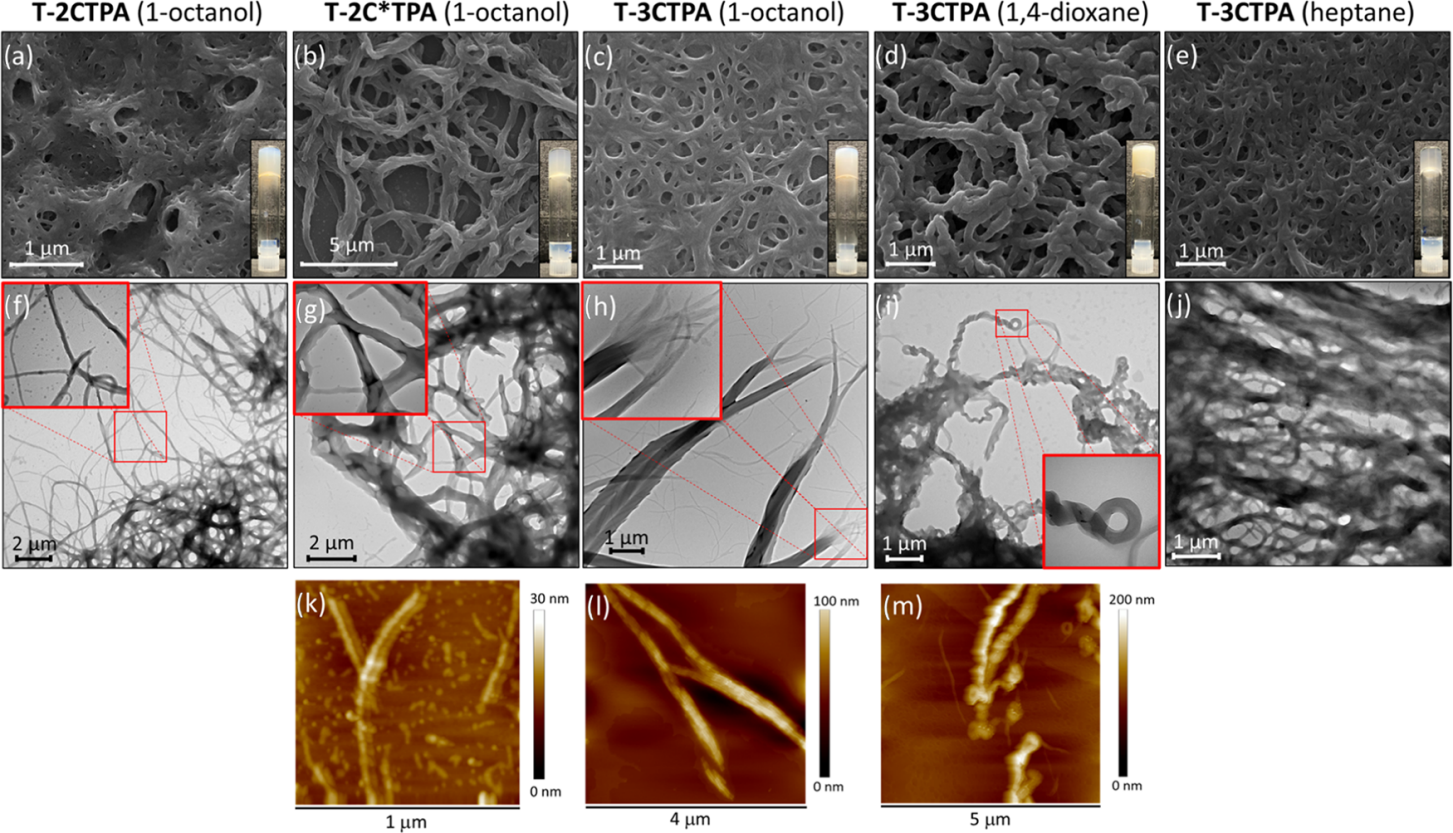
SEM, TEM, and AFM images of xerogels of (a, f) **T-2CTPA** from 1-octanol. (b, g, k) T-2C*TPA from 1-octanol. (c, h, l) **T-3CTPA** from 1-octanol, (d, i, m) **T-3CTPA** from dioxane, and (e, j) **T-3CTPA** from heptane.

The morphology of the aggregates responsible for immobilizing the solvent was observed by SEM, TEM, and AFM in the xerogels prepared from gels at 1 wt % (Figure 7). All three xerogels prepared from 1-octanol show fibrillar morphology. The xerogel of compound **T-2CTPA** showed entangled fibril bundles with a width of 170 nm on average (Figure 7a,f). The xerogel of the chiral analogue T-2C*TPA displays twisted fibers with a mean width of 200 nm (Figure 7b,g). Further details of the twisted structure could be observed in AFM images that show fibers with a coil structure of around 100 nm width and 10 nm medium height (Figure 7k). For the xerogel of compound **T-3CTPA**, fibers with a mean width of 200 nm were observed by SEM (Figure 7c). Moreover, TEM and AFM images showed helical fibril bundles of 400 nm formed by smaller fibers with an average width of 35 nm and a helix pitch of 200 nm (Figure 7h,l). Twisted fibers were also observed for the 1,4-dioxane xerogel of compound **T-3CTPA**, which exhibits helical fibers with a mean width of 150 nm and helix pitches of 150 nm (Figure 7d,i,m). In contrast, the xerogel formed by a hydrocarbon solvent, heptane, displays large bundles of fibers with a mean width of 100 nm (Figure 7e,j).

**Figure 8:**
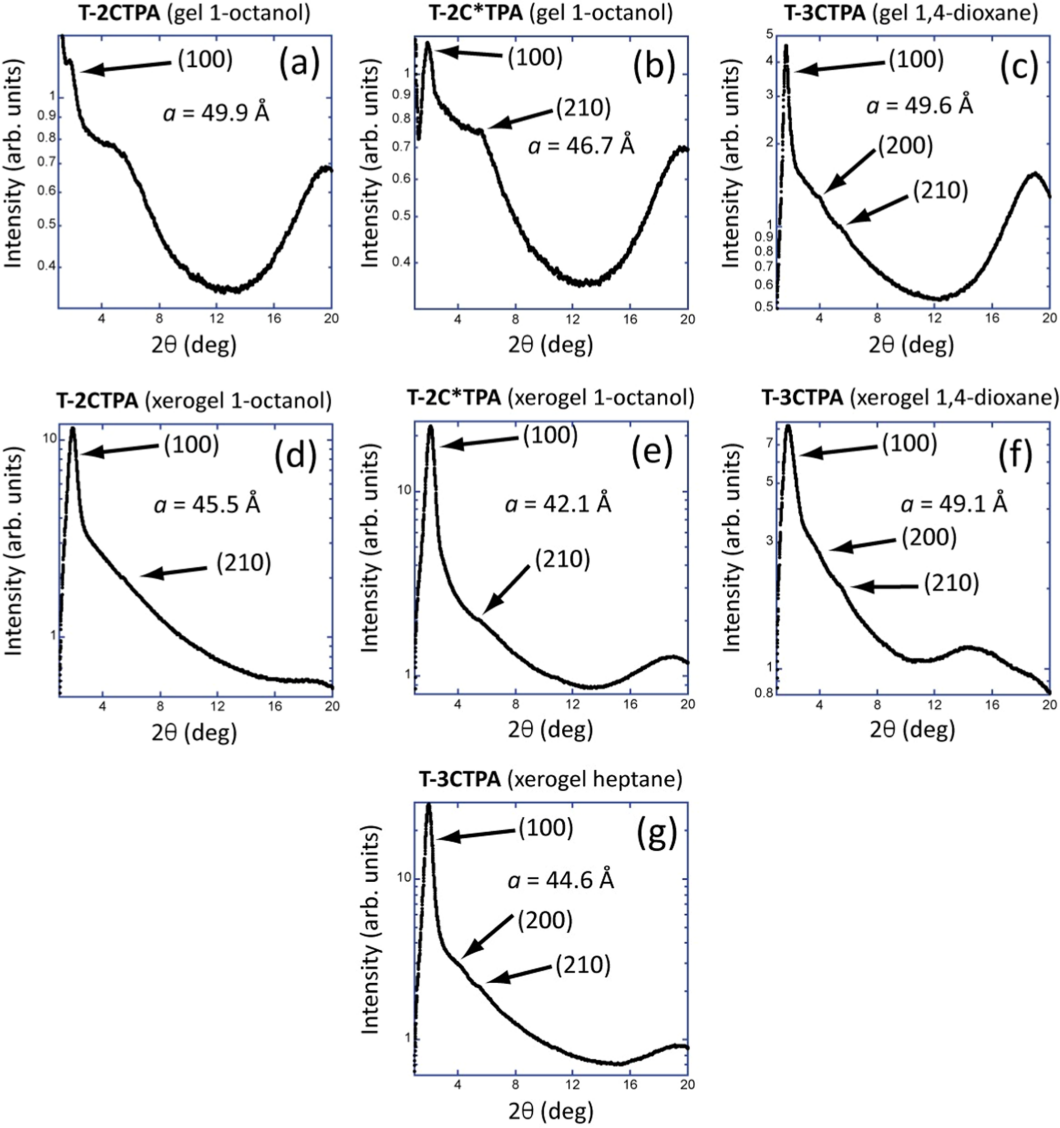
X-ray diffractograms of gels at 5 wt % and xerogels of compounds (a, d) **T-2CTPA** in 1-octanol, (b, e) T-2C*TPA in 1-octanol, (c, f) **T-3CTPA** in 1,4-dioxane, and (g) xerogel of **T-3CTPA** in heptane with their corresponding *a* parameter.

To gain understanding of the molecular arrangement within the fibers, XRD studies were conducted on both gels and their corresponding xerogels. A concentration of 5 wt % was selected to ensure sufficiently intense reflections for accurate measurements (Figure 8). Unfortunately, at this concentration, 1-octanol **T-3CTPA** precipitates in 1-octanol, and therefore XRD studies were not carried out on this sample.

The diffractograms of the xerogels from the 1-octanol gels of **T-2CTPA** (Figure 8d) and T-2C*TPA (Figure 8e) show two maxima in the small angle region corresponding to distances with a ratio of *d*, *d*/√7, which are related with the Miller indexes (100) and (210) of a hexagonal columnar organization with lattice parameters *a* = 45.5 Å for the xerogel of **T-2CTPA** and *a* = 42.1 Å for T-2C*TPA. The corresponding diffractograms in the gel state show for both compounds a broad reflection that corresponds to a periodic distance of 14.6 Å, which can be related with the longitudinal distance of the solvent molecules, and a maximum at wide angles corresponding to a distance of 4.5 Å, due to the liquid-like arrangement of the alkyl chains. At the small angle region, both diffractograms are slightly different. Whereas the T-2C*TPA gel displays the same pattern as the xerogel (Figure 8b,e, respectively), for the **T-2CTPA** gel, only one main reflection is observed (Figure 8a). According to the distances measured, a hexagonal columnar arrangement can be proposed with lattice parameters *a* = 49.9 Å for **T-2CTPA** and *a* = 46.7 Å for T-2C*TPA gels, which are significantly larger than those calculated for the xerogels given the presence of solvent molecules within the columns. Compared with the mesophase, the lattice parameter obtained for the gel of T-2C*TPA is larger, as expected if we consider that 1-octanol can interact with the tris(triazolyl)triazine by hydrogen-bonding interactions forming a larger stacking entity.^34^ However, this increase is not observed for **T-2CTPA**, which shows practically the same lattice parameter in the gel as in the mesophase, larger than that of the xerogel.

For the self-assembly of **T-3CTPA** in 1,4-dioxane, XRD diffractograms of the gel (Figure 8c) and xerogel (Figure 8f) exhibit three reflections in the small angle corresponding to distances with a ratio *d*, *d*/√4, and *d*/√7, and which are related with the reflections (100), (200) and (210) of a hexagonal lattice, with lattice parameters *a* = 49.6 and 49.1 Å, respectively. In the case of heptane, XRD diffractograms in the gel state show only a broad band due to the solvent, whereas the xerogel displays three reflections in the small angle in a ratio *d*, *d*/√4, and *d*/√7, which confirmed a hexagonal arrangement with lattice parameter *a* = 44.6 Å.

**Figure 9:**
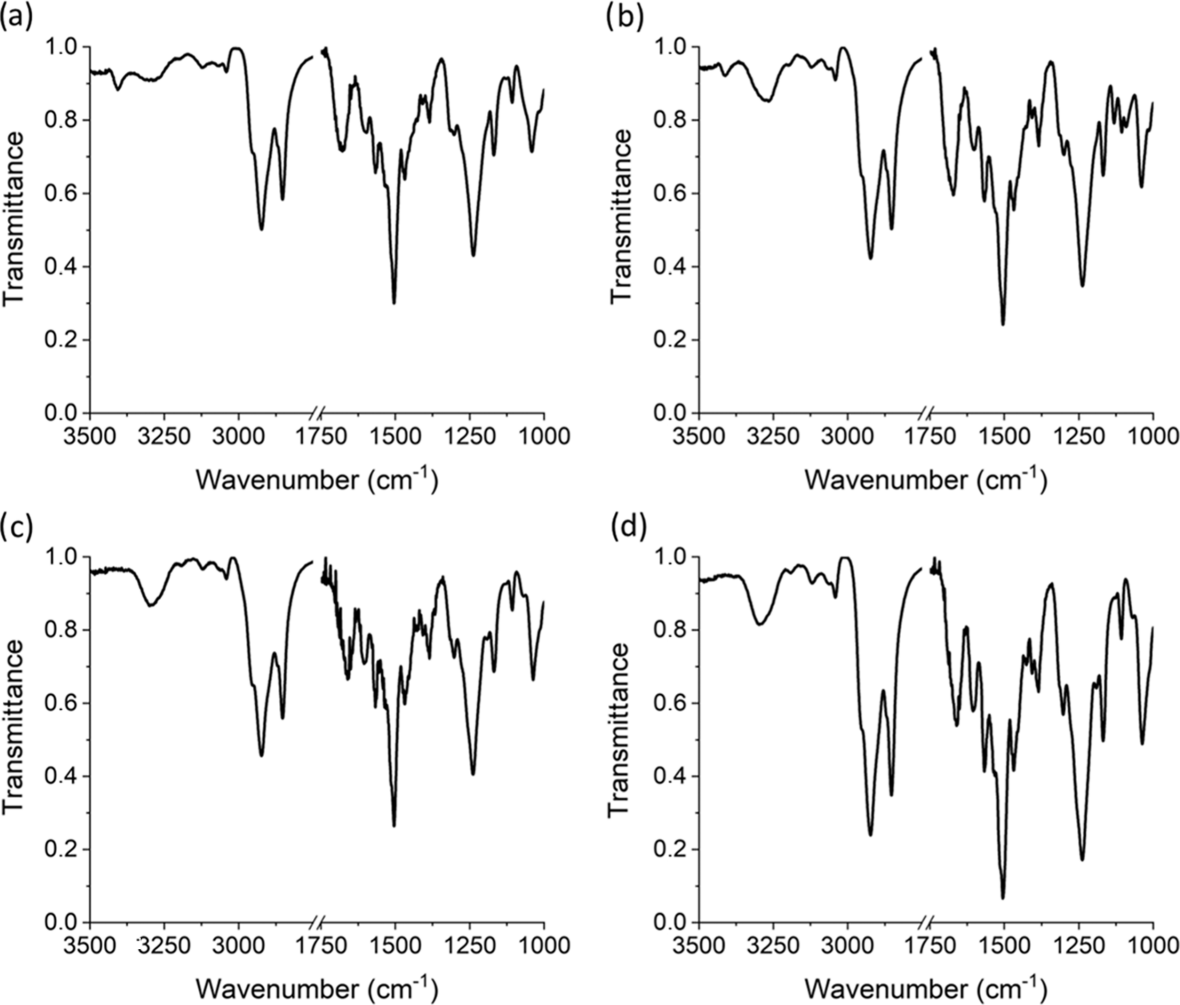
FTIR spectra of xerogels at room temperature: (a) **T-2CTPA** from 1-octanol, (b) T-2C*TPA from 1-octanol, (c) **T-3CTPA** from heptane, (d) **T-3CTPA** from 1,4-dioxane.

Intermolecular hydrogen-bonding interactions in xerogels were also studied by FTIR. As can be seen in Figure 9, the xerogels of the compounds with a shorter spacer **T-2CTPA** and T-2C*TPA show two N–H *st* bands as in the mesophase (Figure 5), one due to free N–H bonds (3410 cm^–1^) and another due to N–H bonds associated by hydrogen bonds (3280 cm^–1^). It can also be seen that the intensity ratio between the hydrogen-bonded and the free N–H *st* bands is significantly different in both compounds, being smaller for the achiral compound **T-2CTPA**. Even more evident than in the mesophase, this must be related with weaker intermolecular hydrogen-bonding interactions within **T-2CTPA** aggregates. This is also confirmed by the C=O *st* band, which appears at higher wavenumbers (1680 cm^–1^) for this compound with respect to its chiral analogue T-2C*TPA (1670 cm^–1^). The xerogels of the compound with the longest spacer **T-3CTPA** showed, as in the mesophase, a single N–H *st* band (3295 cm^–1^) and a sharp C=O *st* band at 1660 cm^–1^, consistent with a full involvement of amide groups in hydrogen-bonding interactions.

### Chiroptical Properties of Gels

The chiroptical properties of the T-2C*TPA gel in 1-octanol were investigated in samples with a concentration of 0.5 wt % at both room temperature and above the gel-to-sol transition (Figure 5c). The CD spectrum recorded at room temperature exhibits intense optical activity across the entire absorption range. However, as the temperature increased, the intensity of the CD signals decreased significantly, and the shape of the curve underwent significant changes. Notably, the CD spectrum recorded above the gel-to-sol transition resembled that of the THF solution (Figure 5a). These observations suggest that molecular chirality in the spacer induces long-range supramolecular chirality along the supramolecular stacks, with the resulting handedness being opposite to the chiral architecture formed in the mesophase. In particular, intense positive CD bands appeared in the gel at 215, 270, and 310 nm, which are associated with π–π* transitions of tris(triazolyl)triazine^35^ and TPA chromophores. A definitive proof of the formation of aggregates with a chiral architecture lies in the negative band around 360 nm. This band only manifested on aggregated species, not in the CD spectrum above the gel–sol transition, the THF solution CD spectrum, or even the CD spectrum of the isotropic liquid. This band is attributed to TPA units arranged in a left-handed helical manner, likely driven by a frozen chiral-propeller conformation.^46^ It is also interesting to remark that this is in contrast to the mesophase, in which this band is visible with opposite sign. These results provide compelling evidence for the formation of aggregates with a chiral architecture in the gel phase, consistently with AFM observations.

## Conclusions

In order to prepare well-defined and stable columnar LCs including the hole-transporting TPA unit, a new family of *C*_3_-symmetric star-shaped tricarboxamides with flexible amide spacers linking a tris(triazolyl)triazine core with three TPA peripheral units have been synthesized via CuAAC reaction. The introduction of amide linkages fosters intermolecular hydrogen bonding which, in turn, strengthens π-stacking and van der Waals interactions as elucidated by FTIR spectroscopy. This results in the promotion of columnar liquid crystalline behavior across a wide temperature range despite of the low tendency of nonplanar TPA units to form liquid crystal phases. The length of the flexible spacer influences significantly the mesophase behavior of the new mesogens. The derivatives with the shortest spacers (**T2-CTPA** and T-2C*TPA) display hexagonal columnar arrangements within the whole mesomorphic range, whereas the longest spacer (**T-3CTPA**) induces a tetragonal columnar phase at high temperature. Furthermore, intermolecular hydrogen-bonding interactions also favor self-assembly in organic solvents, leading to the formation of organogels with fibrillar morphologies and hexagonal columnar molecular arrangements as confirmed by their XRD studies.

Charge transport properties were studied by the SCLC method at room temperature in solution-processed thin films. The results validate that the inclusion of TPA functional units imparts favorable semiconducting properties to the LC materials, characterized by high hole mobility values within the range of 10^–2^ cm^2^ s^–1^ V^–1^.

The presence of a stereogenic center in the spacer favors the transmission of molecular chirality to the aggregates of the columnar phases in which both the T core and the TPA units arrange in a chiral environment. The transmission of chirality is also observed in the aggregates that form a gel in 1-octanol by AFM and CD, exhibiting CD spectra compatible with TPA units arranged in left-handed helical manner. Chirality does not seem to exert a significant influence on improving mobility values, despite the helical disposition of molecules that could facilitate orbital overlapping. Nevertheless, the presence of the chiral spacer does endow the compound with lower transition temperatures and enhanced stability for thermal treatments, giving rise to improved columnar alignment and more uniform measurements.

In this respect, the fact that these compounds show a dual liquid crystalline/gel behavior offers the opportunity for processing not only in bulk to harness the order of the columnar mesophase but also as gels to produce fibers with a similar columnar arrangement. This opens the door to explore novel device configurations for evaluating semiconducting properties.

## Experimental Section

### Chemicals

All reagents were purchased from Aldrich or Fisher Scientific and used without further purification. Anhydrous CH_2_Cl_2_ and THF were purchased from Scharlab and dried by using a solvent purification system.

### Synthesis and Characterization of TPA-Tris(triazolyl)triazine Derivatives

The experimental procedures for the synthesis of the intermediates are reported in the Supporting Information. The final compounds **T-2CTPA**, T-2C*TPA, and **T-3CTPA** were synthesized according to the following procedure: CuSO_4_·5H_2_O (0.15 mmol), aryl azide (3.3 mmol), sodium ascorbate (0.3 mmol), and TBTA (0.15 mmol) were added to a mixture of H_2_O/*^t^*BuOH/CH_2_Cl_2_ (1:2:8, 33 mL) under Ar atmosphere and vigorous stirring. After 15 min, 2,4,6-tris(trimethylsilylethynyl)-1,3,5-triazine (1 mmol) was added and the solution turned red immediately. Then, KF solution (3.3 mmol in 1 mL of water) was added dropwise over 2 h and the mixture was stirred overnight at room temperature in the darkness. The mixture was diluted with CH_2_Cl_2_ (30 mL) and the organic phase was washed with aqueous 0.1 M EDTA-Na_2_ solution (3 × 30 mL) and brine (3 × 30 mL). The organic layer was dried with MgSO_4_, filtrated, and the solvent was removed under reduced pressure. **T-2CTPA:** The crude was purified by flash chromatography using DCM/MeOH (9:1) and the product was obtained as a yellow-brown solid. Yield: 61%. ^**1**^**H NMR** (400 MHz, CDCl_3_): δ 9.11 (s, 3H, triazole), 8.22 (s, 3H, NH) 7.90–7.79 (m, 6H, ArH), 7.46–7.36 (m, 6H, ArH), 7.22–7.14 (m, 6H, ArH), 7.05–6.98 (m, 12H, ArH), 6.97–6.91 (m, 6H, ArH), 6.85–6.75 (m, 12H, ArH), 4.69 (s, 6H, OCH_2_) 3.93 (t, *J* = 6.4 Hz, 12H, OCH_2_), 1.84–1.71 (m, 12H, CH_2_), 1.52–1.18 (m, 108H, CH_2_), 0.89 (t, *J* = 6.5 Hz, 18H, CH_3_). ^**13**^**C NMR** (100 MHz, CDCl_3_): δ 166.8, 165.3, 157.8, 155.5, 146.3, 146, 140.9, 131.3, 129.7, 126.3, 125.9, 122.6, 121.7, 121.6, 116.1, 115.4, 68.4, 68.1, 32.1, 29.8, 29.8, 29.8, 29.6, 29.5, 29.5, 26.2, 22.8, 14.3. **IR** (KBr, cm^–1^): 3406 (NH free), 3305 (NH associated), 3041 (C_Ar_–H), 2924 (Csp^3^-H), 2853 (Csp^3^-H), 1680 (C=O), 1595 (C–C_Ar_), 1564 (NH δ), 1503 (C–C_Ar_), 1467 (C–C_Ar_), 1237 (C–O). **MS** (MALDI^+^, dithranol): 2564.58 [M]^+^. **EA** calculated (%) for C_159_H_210_N_18_O_12_: C 74.44, H 8.25, N 9.83; found: C 74.76, H 8.15, N 9.61. T-2C*TPA: The crude was purified by flash chromatography using DCM/MeOH (95:5) and the product was obtained as a yellow-brown solid. Yield: 52%. ^**1**^**H NMR** (400 MHz, CDCl_3_): δ 9.10 (s, 3H, triazole), 8.11 (s, 3H, NH) 7.85–7.77 (m, 6H, ArH), 7.42–7.33 (m, 6H, ArH), 7.20–7.11 (m, 6H, ArH), 7.03–6.95 (m, 12H, ArH), 6.94–6.88 (m, 6H, ArH), 6.83–6.74 (m, 12H, ArH), 4.86 (q, *J* = 6.5 Hz, 3H, OCH_2_), 3.90 (t, *J* = 6.4 Hz, 12H, OCH_2_), 1.80–1.68 (m, 21H, CH_2_ + CH_3_), 1.50–1.20 (m, 108H, CH_2_), 0.87 (t, *J* = 6.4 Hz, 18H, CH_3_). ^**13**^**C NMR** (100 MHz, CDCl_3_): δ 169.3, 166.9, 157.6, 155.5, 146.2, 146, 141, 131.3, 130, 126.2, 126, 122.8, 121.8, 121.4, 117, 115.4, 76.2, 68.5, 32.1, 29.8, 29.8, 29.7, 29.7, 29.6, 29.5, 29.5, 26.2, 22.8, 14.2. **IR** (KBr, cm^–1^): 3406 (NH free), 3305 (NH associated), 3042 (C_Ar_–H), 2923 (Csp^3^-H), 2852 (Csp^3^-H), 1680 (C=O), 1599 (C–C_Ar_), 1567 (NH δ), 1505 (C–C_Ar_), 1469 (C–C_Ar_), 1238 (C–O). **MS** (MALDI+, dithranol): 2606.43 [M]^+^. **EA** calcd (%) for C_162_H_216_N_18_O_12_: C 74.62, H 8.35, N 9.67; found: C 74.56, H 8.44, N 9.60. **T-3CTPA:** The crude was purified by flash chromatography using DCM/MeOH (9:1) and the product was obtained as a brown solid. Yield: 55%. ^**1**^**H NMR** (400 MHz, tetrahydrofuran-d_8_): δ 9.43 (s, 3H, triazole), 9.07 (s, 3H, NH), 8.04–7.95 (m, 6H, ArH), 7.53–7.42 (m, 6H, ArH), 7.22–7.11 (m, 6H, ArH), 6.98–6.89 (m, 12H, ArH), 6.88–6.82 (m, 6H, ArH), 6.81–6.73 (m, 12H, ArH), 4.42 (t, *J* = 6.3 Hz, 6H, OCH_2_), 3.91 (t, *J* = 6.4 Hz, 12H, OCH_2_), 2.79 (t, *J* = 6.3 Hz, 6H, CH_2_), 1.50–1.20 (m, 108H, CH_2_), 0.89 (t, *J* = 6.8 Hz, 18H, CH_3_). ^**13**^**C NMR** (100 MHz, tetrahydrofuran-d_8_): δ 168.5, 168.1, 160.4, 156.3, 145.6, 142.5, 134.5, 131.6, 126.8, 123.1, 122.7, 121.1, 116.3, 68.9, 65.8, 37.7, 33, 30.8, 30.8, 30.6, 30.6, 30.5, 27.3, 23.7, 14.6. **IR** (KBr, cm^–1^): 3295 (NH associated), 3042 (C_Ar_–H), 2923 (Csp^3^-H), 2852 (Csp^3^-H), 1660 (C=O), 1602 (C–C_Ar_), 1567 (NH δ), 1504 (C–C_Ar_), 1469 (C–C_Ar_), 1239 (C–O). **MS** (MALDI+, dithranol): 2606.70 [M]^+^. **EA** calcd (%) for C_162_H_216_N_18_O_12_: C 74.62, H 8.35, N 9.67; found: C 74.83, H 8.54, N 9.45.

### Liquid Crystal Properties

The mesophases were examined by **polarizing optical microscopy** (POM) using a polarizing optical microscope Olympus BX51 equipped with an Olympus DP152 digital camera and connected to a Linkam THMS600 hot stage and a Linkam TMS94 controller. Transition temperatures and enthalpies were obtained by differential scanning calorimetry (DSC) with DSC TA Instruments Q20 and Q2000 at heating and cooling rates of 20 °C min^–1^. The apparatus was previously calibrated with indium (156.6 °C, 28.71 J g^–1^). Powder X-ray experiments were performed in a Pinhole diffractometer (Anton Paar) operating with a point focused Ni-filtered Cu–Kα beam. The samples were held in Lindemann glass capillaries (0.9 mm diameter) and heated with a variable-temperature attachment. The diffraction patterns were collected on photographic films. Gel and xerogel X-ray diffraction diagrams were recorded using a Stoe Stadivari goniometer equipped with a Genix3D microfocus generator (Xenocs) and a Dectris Pilatus 100 K detector. Temperature control was achieved using a nitrogen-gas Cryostream controller (Oxford Cryosystems) allowing for a temperature control of about 0.1 °C. Lindemann capillaries of diameter 0.6 mm were utilized. In the case of gels and xerogels, the materials were held in loops of 300–500 μm in diameter (MiTeGen). Monochromatic Cu–Kα radiation (λ = 1.5418 Å) was used. The exposure time was 2 min. For variable-temperature FTIR experiments, the samples were prepared on KBr pellets with a concentration of the product of 1–2% (w/w). The pellets were heated in a Mettler FP80 HT hot stage. For polarized FTIR experiments, a ZnSe polarizer from Pike Technologies was used and the materials were placed between two KBr polished IR crystal windows (13 mm diameter × 2 mm thickness) purchased from Aldrich. Charge mobility of the three liquid crystal materials was measured by the space charge limited current (SCLC) method in solution-processed samples. Hole mobility devices were prepared by spin-coating 110 μL of a solution of the material in CHCl_3_ (350 nm thickness, 30 mg/mL at 1500 rpm for 1 min) onto a glass covered by 3 ITO stripes. Afterward, 3 Au stripes (100 nm thickness) orthogonal to ITO stripes were deposited on top of the material layer by evaporation under vacuum. Electron mobility devices were prepared by spin-coating a solution of CsCO_3_ in 2 methoxy ethanol (3 mg/mL, 4500 rpm for 60 s) onto a glass covered by 3 ITO stripes and the CsCO_3_ layer was annealed at 120 °C for 10 min. After that, a material layer was placed by spin-coating 110 μL of a solution of the material in CHCl_3_ (350 nm thickness, 30 mg/mL at 1500 rpm for 1 min). On top of it, 3 Al stripes (100 nm thickness) orthogonal to ITO stripes were deposited on top of the material layer by evaporation under vacuum. The chiroptical properties of T-2C*TPA in the mesophase were studied by circular dichroism in a Jasco J-810 spectropolarimeter. The thin film was prepared as follows: a 100 μL CHCl_3_ solution of the material was spin-coated onto a quartz plate (30 mg/mL at 1500 rpm for 1 min), heated to the isotropic liquid, and slowly cooled to room temperature to promote proper mesophase formation. CD spectra were recorded at different rotation angles around the light beam showed the same trace and were averaged in order to compensate for linear dichroism artifacts (Figure SI16).

### Gelation Properties

Gels were prepared by heating the materials in the solvent at 130 °C for 1-octanol and 90 °C for 1,4-dioxane and heptane until the material was completely dissolved. Then, the solution was slowly cooled at room temperature. The morphological characterization of the gels was carried out by transmission electron microscopy (TEM) using a TECNAI G2 20 operating at 200 kV (accelerating voltage). The samples were prepared by depositing one drop of dispersion on a carbon film copper grid, drying on air, and negatively stained with uranyl acetate prior to observation for better contrast. Scanning electron microscopy (SEM) images were recorded using an INSPECT-F50 operating at 10 kV (accelerating voltage). The samples were prepared by depositing one drop of dispersion on a glass slide and drying it on air. The surface of the samples was covered with a Pd coat. Atomic force microscopy (AFM) topographic images were obtained using a Multimode 8 microscope equipped with a Nanoscope V control unit from Bruker at a scan rate of 1.0–1.2 Hz, using Tapping mode. The data were collected using RTESPA-150 tips (nominal frequency of 150 kHz, from Bruker) in air. Circular dichroism experiments in the gel state at variable temperatures were carried out using a Jasco CDF-426S sample holder with a 0.1 mm quartz cell. The preparation of the sample was carried out by introducing the gel into the measurement cuvette, heating it to the sol state, and cooling down slowly to room temperature.

## Supporting Information Available

Experimental techniques, synthetic procedures, chemical characterization data, liquid crystalline properties, FTIR studies, CD studies, electrochemical properties, charge mobility measurements, and NMR studies (PDF)

## References

[ref1] Shen Z.; Huang W.; Li L.; Li H.; Huang J.; Cheng J.; Fu Y. Research Progress of Organic Field-Effect Transistor Based Chemical Sensors. Small 2023, 19 (41), 2302406 10.1002/smll.202302406. 37271887

[ref2] Nayak D.; Choudhary R. B. A survey of the structure, fabrication, and characterization of advanced organic light emitting diodes. Microelectron. Reliab. 2023, 144, 114959 10.1016/j.microrel.2023.114959.

[ref3] Tarique W. B.; Uddin A. A review of progress and challenges in the research developments on organic solar cells. Mater. Sci. Semicond. Process. 2023, 163, 107541 10.1016/j.mssp.2023.107541.

[ref4] Köhler A. Electronic and Optical Processes of Organic Semiconductors. In Electronic Processes in Organic Semiconductors; Wiley, 2015; pp 193–305.

[ref5] Termine R.; Golemme A. Charge Mobility in Discotic Liquid Crystals. Int. J. Mol. Sci. 2021, 22 (2), 877 10.3390/ijms22020877. 33467214 PMC7830985

[ref6] Bala I.; De J.; Pal S. K. Functional Discotic Liquid Crystals Through Molecular Self-Assembly: Toward Efficient Charge Transport Systems. In Molecular Architectonics and Nanoarchitectonics; Govindaraju T.; Ariga K., Eds.; Springer Singapore: Singapore, 2022; pp 89–130.

[ref7] Giménez R.; Martínez-Bueno A.; Sierra T. Noncovalent Design of Columnar LCs on the Way to Nanostructured Functional Materials. In Supramolecular Nanotechnology; Azzaroni O.; Conda-Sheridan M., Eds.; Wiley-VCH: Weinheim, 2023; Vol. 1, pp 425–446.

[ref8] Miyajima D.; Araoka F.; Takezoe H.; Kim J.; Kato K.; Takata M.; Aida T. Electric-Field-Responsive Handle for Large-Area Orientation of Discotic Liquid-Crystalline Molecules in Millimeter-Thick Films. Angew. Chem., Int. Ed. 2011, 50 (34), 7865–7869. 10.1002/anie.201102472. 21732509

[ref9] Sato K.; Itoh Y.; Aida T. Columnarly Assembled Liquid-Crystalline Peptidic Macrocycles Unidirectionally Orientable over a Large Area by an Electric Field. J. Am. Chem. Soc. 2011, 133 (35), 13767–13769. 10.1021/ja203894r. 21627140

[ref10] Guilleme J.; Aragó J.; Ortí E.; Cavero E.; Sierra T.; Ortega J.; Folcia C. L.; Etxebarria J.; González-Rodríguez D.; Torres T. A columnar liquid crystal with permanent polar order. J. Mater. Chem. C 2015, 3 (5), 985–989. 10.1039/C4TC02662D.

[ref11] Nguyen M. L.; Shin T. J.; Kim H.-J.; Cho B.-K. Oriented columnar films of a polar 1,2,3-triazole-based liquid crystal prepared by applying an electric field. J. Mater. Chem. C 2017, 5 (32), 8256–8265. 10.1039/C7TC02710A.

[ref12] Zhang C.; Nakano K.; Nakamura M.; Araoka F.; Tajima K.; Miyajima D. Noncentrosymmetric Columnar Liquid Crystals with the Bulk Photovoltaic Effect for Organic Photodetectors. J. Am. Chem. Soc. 2020, 142 (7), 3326–3330. 10.1021/jacs.9b12710. 32024364

[ref13] Adam D.; Schuhmacher P.; Simmerer J.; Häussling L.; Siemensmeyer K.; Etzbachi K. H.; Ringsdorf H.; Haarer D. Fast photoconduction in the highly ordered columnar phase of a discotic liquid crystal. Nature 1994, 371 (6493), 141–143. 10.1038/371141a0.

[ref14] Xiao Y.; Su X.; Sosa-Vargas L.; Lacaze E.; Heinrich B.; Donnio B.; Kreher D.; Mathevet F.; Attias A.-J. Chemical engineering of donor–acceptor liquid crystalline dyads and triads for the controlled nanostructuration of organic semiconductors. CrystEngComm 2016, 18 (25), 4787–4798. 10.1039/C6CE00365F.

[ref15] Feringán B.; Romero P.; Serrano J. L.; Folcia C. L.; Etxebarria J.; Ortega J.; Termine R.; Golemme A.; Giménez R.; Sierra T. H-Bonded Donor–Acceptor Units Segregated in Coaxial Columnar Assemblies: Toward High Mobility Ambipolar Organic Semiconductors. J. Am. Chem. Soc. 2016, 138 (38), 12511–12518. 10.1021/jacs.6b06792. 27577722

[ref16] An Z.; Yu J.; Jones S. C.; Barlow S.; Yoo S.; Domercq B.; Prins P.; Siebbeles L. D. A.; Kippelen B.; Marder S. R. High Electron Mobility in Room-Temperature Discotic Liquid-Crystalline Perylene Diimides. Adv. Mater. 2005, 17 (21), 2580–2583. 10.1002/adma.200500027.

[ref17] Bala I.; Singh N.; Yadav R. A. K.; De J.; Gupta S. P.; Singh D. P.; Dubey D. K.; Jou J.-H.; Douali R.; Pal S. K. Room temperature perylene based columnar liquid crystals as solid-state fluorescent emitters in solution-processable organic light-emitting diodes. J. Mater. Chem. C 2020, 8 (36), 12485–12494. 10.1039/D0TC02754E.

[ref18] Debije M. G.; Piris J.; de Haas M. P.; Warman J. M.; Tomović Ž.; Simpson C. D.; Watson M. D.; Müllen K. The Optical and Charge Transport Properties of Discotic Materials with Large Aromatic Hydrocarbon Cores. J. Am. Chem. Soc. 2004, 126 (14), 4641–4645. 10.1021/ja0395994. 15070380

[ref19] De J.; Bala I.; Gupta S. P.; Pandey U. K.; Pal S. K. High Hole Mobility and Efficient Ambipolar Charge Transport in Heterocoronene-Based Ordered Columnar Discotics. J. Am. Chem. Soc. 2019, 141 (47), 18799–18805. 10.1021/jacs.9b09126. 31682432

[ref20] De R.; De J.; Gupta S. P.; Bala I.; Ankita; Tarun; Pandey U. K.; Pal S. K. Oxadiazole-integrated heterocoronene discotics as ambipolar organic semiconductors. J. Mater. Chem. C 2023, 11 (3), 980–985. 10.1039/D2TC04144H.

[ref21] Feringán B.; Termine R.; Golemme A.; Granadino-Roldán J. M.; Navarro A.; Giménez R.; Sierra T. Triphenylamine- and triazine-containing hydrogen bonded complexes: liquid crystalline supramolecular semiconductors. J. Mater. Chem. C 2021, 9 (6), 1972–1982. 10.1039/D0TC05186A.

[ref22] Wang Y.-J.; Sheu H.-S.; Lai C. K. New star-shaped triarylamines: synthesis, mesomorphic behavior, and photophysical properties. Tetrahedron 2007, 63 (7), 1695–1705. 10.1016/j.tet.2006.11.058.

[ref23] Majumdar K. C.; Pal N.; Debnath P.; Rao N. V. S. A columnar mesophase from a disc-shaped molecule derived from triphenylamine: synthesis, mesomorphic behaviour and optical properties. Tetrahedron Lett. 2007, 48 (36), 6330–6333. 10.1016/j.tetlet.2007.07.026.

[ref24] Majumdar K. C.; Chattopadhyay B.; Shyam P. K.; Pal N. A new disc-shaped mesogenic compound with olefinic linkage derived from triphenylamine: synthesis, mesogenic behavior and fluorescence properties. Tetrahedron Lett. 2009, 50 (49), 6901–6905. 10.1016/j.tetlet.2009.09.140.

[ref25] Reghu R. R.; Simokaitiene J.; Grazulevicius J. V.; Raisys S.; Kazlauskas K.; Jursenas S.; Jankauskas V.; Reina A. Synthesis and properties of hole-transporting triphenylamine-derived dendritic compounds. Dyes Pigm. 2015, 115, 135–142. 10.1016/j.dyepig.2014.12.013.

[ref26] Domoto Y.; Busseron E.; Maaloum M.; Moulin E.; Giuseppone N. Control over Nanostructures and Associated Mesomorphic Properties of Doped Self-Assembled Triarylamine Liquid Crystals. Chem. - Eur. J. 2015, 21 (5), 1938–1948. 10.1002/chem.201405567. 25483214

[ref27] Liu X.; Li N.; Pang Y.; Li S.; Xiao Y. Synthesis, self-assembly and photophysical properties of AIEE-active triphenylamine-based discotic liquid crystals. J. Iran. Chem. Soc. 2022, 19 (11), 4411–4421. 10.1007/s13738-022-02611-x.

[ref28] Feringán B.; Martínez-Bueno A.; Sierra T.; Giménez R. Triphenylamine-Containing Benzoic Acids: Synthesis, Liquid Crystalline and Redox Properties. Molecules 2023, 28 (7), 2887 10.3390/molecules28072887. 37049649 PMC10096164

[ref29] Castillo-Vallés M.; Martínez-Bueno A.; Giménez R.; Sierra T.; Ros M. B. Beyond liquid crystals: new research trends for mesogenic molecules in liquids. J. Mater. Chem. C 2019, 7 (46), 14454–14470. 10.1039/C9TC04179F.

[ref30] Moulin E.; Armao J. J. I. V.; Giuseppone N. Triarylamine-Based Supramolecular Polymers: Structures, Dynamics, and Functions. Acc. Chem. Res. 2019, 52 (4), 975–983. 10.1021/acs.accounts.8b00536. 30915835

[ref31] Moulin E.; Niess F.; Maaloum M.; Buhler E.; Nyrkova I.; Giuseppone N. The Hierarchical Self-Assembly of Charge Nanocarriers: A Highly Cooperative Process Promoted by Visible Light. Angew. Chem., Int. Ed. 2010, 49 (39), 6974–6978. 10.1002/anie.201001833. 20645369

[ref32] Faramarzi V.; Niess F.; Moulin E.; Maaloum M.; Dayen J.-F.; Beaufrand J.-B.; Zanettini S.; Doudin B.; Giuseppone N. Light-triggered self-construction of supramolecular organic nanowires as metallic interconnects. Nat. Chem. 2012, 4 (6), 485–490. 10.1038/nchem.1332. 22614384

[ref33] Ellis T. K.; Galerne M.; Armao J. J. IV; Osypenko A.; Martel D.; Maaloum M.; Fuks G.; Gavat O.; Moulin E.; Giuseppone N. Supramolecular Electropolymerization. Angew. Chem., Int. Ed. 2018, 57 (48), 15749–15753. 10.1002/anie.201809756. 30288878

[ref34] Martínez-Bueno A.; Vidal R.; Ortega J.; Etxebarria J.; Folcia C. L.; Giménez R.; Sierra T. Soft nanostructures out of star-shaped triazines with flexible amide spacers: liquid crystals with a cubic to columnar transition with memory effect, gels and supramolecular chirality. Mater. Today Chem. 2023, 29, 101394 10.1016/j.mtchem.2023.101394.

[ref35] Beltrán E.; Serrano J. L.; Sierra T.; Giménez R. Tris(triazolyl)triazine via Click-Chemistry: A C3 Electron-Deficient Core with Liquid Crystalline and Luminescent Properties. Org. Lett. 2010, 12 (7), 1404–1407. 10.1021/ol902900y. 20196614

[ref36] El Malah T.; Nour H. F.; Nayl A. A.; Elkhashab R. A.; Abdel-Megeid F. M. E.; Ali M. M. Anticancer Evaluation of Tris(triazolyl)triazine Derivatives Generated via Click Chemistry. Aust. J. Chem. 2016, 69 (8), 905–910. 10.1071/CH16006.

[ref37] Smith W. B. Observations on the reduction of aryl nitro groups with palladium-sodium borohydride. J. Heterocycl. Chem. 1987, 24 (3), 745–748. 10.1002/jhet.5570240340.

[ref38] Feringán B.; Romero P.; Serrano J. L.; Giménez R.; Sierra T. Supramolecular Columnar Liquid Crystals Formed by Hydrogen Bonding between a Clicked Star-Shaped s-Triazine and Benzoic Acids. Chem. - Eur. J. 2015, 21 (24), 8859–8866. 10.1002/chem.201500477. 25962742

[ref39] Beltrán E.; Serrano J. L.; Sierra T.; Giménez R. Functional star-shaped tris(triazolyl)triazines: columnar liquid crystal, fluorescent, solvatofluorochromic and electrochemical properties. J. Mater. Chem. 2012, 22 (16), 7797–7805. 10.1039/c2jm15648b.

[ref40] Feringán B.; Cerdá J.; Diosdado B.; Aragó J.; Ortí E.; Giménez R.; Sierra T. On the Structure and Chiral Aggregation of Liquid Crystalline Star-Shaped Triazines H-Bonded to Benzoic Acids. Chem. - Eur. J. 2020, 26 (66), 15313–15322. 10.1002/chem.202001271. 32608135

[ref41] Gan K. P.; Yoshio M.; Kato T. Columnar liquid-crystalline assemblies of X-shaped pyrene–oligothiophene conjugates: photoconductivities and mechanochromic functions. J. Mater. Chem. C 2016, 4 (22), 5073–5080. 10.1039/C6TC00808A.

[ref42] Chiu K. Y.; Su T. X.; Hong Li J.; Lin T.-H.; Liou G.-S.; Cheng S.-H. Novel trends of electrochemical oxidation of amino-substituted triphenylamine derivatives. J. Electroanal. Chem. 2005, 575 (1), 95–101. 10.1016/j.jelechem.2004.09.005.

[ref43] Bala I.; Kaur H.; Maity M.; Yadav R. A. K.; De J.; Gupta S. P.; Jou J.-H.; Pandey U. K.; Pal S. K. Electroluminescent Aggregation-Induced Emission-Active Discotic Liquid Crystals Based on Alkoxy Cyanostilbene-Functionalized Benzenetricarboxamide with Ambipolar Charge Transport. ACS Appl. Electron. Mater. 2022, 4 (3), 1163–1174. 10.1021/acsaelm.1c01251.

[ref44] Dhingra S.; Bala I.; De J.; Gupta S. P.; Pandey U. K.; Pal S. K. An electron-deficient tris(triazole)-based discotic liquid crystal that exhibits fast electron transport. J. Mater. Chem. C 2021, 9 (17), 5628–5632. 10.1039/D1TC00866H.

[ref45] Castillo-Vallés M.; Beltrán E.; Cerdá J.; Aragó J.; Romero P.; Serrano J. L.; Ortí E.; Giménez R.; Sierra T. Self-Assembly of Clicked Star-Shaped Triazines into Functional Nanostructures. ChemNanoMat 2019, 5 (1), 130–137. 10.1002/cnma.201800484.

[ref46] Osypenko A.; Moulin E.; Gavat O.; Fuks G.; Maaloum M.; Koenis M. A. J.; Buma W. J.; Giuseppone N. Temperature Control of Sequential Nucleation–Growth Mechanisms in Hierarchical Supramolecular Polymers. Chem. - Eur. J. 2019, 25 (56), 13008–13016. 10.1002/chem.201902898. 31318991

