# Supplementary material for: Effect of Hydrogen Bonding and Chirality in Star-Shaped Molecules with Peripheral Triphenylamines: Liquid Crystal Semiconductors and Gels
Source: Chem Mater. 2024 May 2;36(9):4343–56. doi: 10.1021/acs.chemmater.3c03241 (PMC11104488; doi:10.1021/acs.chemmater.3c03241)
Supplement: Supplementary file 1 — cm3c03241_si_001.pdf [file cm3c03241_si_001.pdf]

SUPPORTING INFORMATION

Effect of hydrogen bonding and chirality in star-shaped molecules with peripheral triphenylamines: Liquid crystal semiconductors and gels

Alejandro Martínez-Bueno,[&] *Santiago Martín,*[#] *Josu Ortega,*[§] *César L. Folcia,*[§] *Roberto Termine,*[φ] *Attilio Golemme,*[φ] *Raquel Giménez,*[&*] *Teresa Sierra*[&*]

[&] Instituto de Nanociencia y Materiales de Aragón (INMA). Departamento de Química Orgánica, Facultad de Ciencias, CSIC-Universidad de Zaragoza, 50009 Zaragoza, Spain.

[#] Instituto de Nanociencia y Materiales de Aragón (INMA). Departamento de Química Física, Facultad de Ciencias, CSIC-Universidad de Zaragoza, 50009 Zaragoza, Spain. Laboratorio de Microscopias Avanzadas (LMA), Universidad de Zaragoza, 50018, Zaragoza, Spain.

[§] Department of Physics, Faculty of Science and Technology, Universidad del País Vasco UPV/EHU, 48080 Bilbao, Spain

[φ] CNR-NANOTEC SS di Rende, Dipartimento di Fisica, Università della Calabria, 87036 Rende (Italy).

Experimental details 2
Synthesis and characterization 3
DSC thermograms 20
X-Ray Diffraction 22
Density estimation 23
Calculation of Z 24
FTIR studies at variable temperature 25
CD spectra of T-2C*TPA 27
Electrochemical properties 29
Charge mobility measurements 30
Concentration dependent NMR experiments 33

Experimental details

All reagents were purchased from Aldrich or Fisher Scientific and used without further purification. Anhydrous CH_2Cl_2 and THF were purchased from Scharlab and dried by using a solvent purification system.

^{1}H-NMR and ^{13}C-NMR spectra were acquired on a Bruker AV400 spectrometer. The experiments were performed at room temperature in different deuterated solvents ($CDCl_3$, CD_2Cl_2 and THF-d8). Chemical shifts are given in ppm relative to TMS and the solvent residual peak was used as internal standard.

Infrared spectra were recorded on a Bruker Vertex 70 FT-IR spectrometer. For room temperature experiments, the samples were prepared on KBr pellets with a concentration of the product of 1-2% (w/w). For variable temperature experiments, KBr pellets were heated in a Mettler FP80 HT hot stage. For polarized FTIR experiments, a ZnSe polarizer from Pike technologies was used and the materials were placed between two KBr polished IR crystal windows (13 mm diameter x 2 mm thickness) purchased from Aldrich.

MALDI-TOF **mass spectrometry** was performed on an Autoflex Bruker mass spectrometer with a dithranol matrix. Positive and negative ion electrospray ionization high resolution (ESI HR$^+$ and ESI HR$^-$) was performed on a Bruker Q-TOF-MS in a positive or negative ESI mode.

The mesophases were examined by **polarizing optical microscopy** using a polarizing optical microscope Olympus BX51 equipped with an Olympus DP152 digital camera and connected to a Linkam THMS600 hot stage and a Linkam TMS94 controller. Transition temperatures and enthalpies were obtained by **differential scanning calorimetry** with DSC TA instruments Q20 and Q2000 at heating and cooling rates of 20 °C min^{-1}. The apparatus was previously calibrated with indium (156.6 °C, 28.71 J g^{-1}).

Powder X-ray experiments were performed in a Pinhole diffractometer (Anton Paar) operating with a point focused Ni-filtered Cu-Kα beam. The samples were held in Lindemann glass capillaries (0.9 mm diameter) and heated with a variable-temperature attachment. The diffraction patterns were collected on photographic films. Gel and xerogel X-ray diffraction diagrams were recorded using a Stoe Stadivari goniometer equipped with a Genix3D microfocus generator (Xenocs) and a Dectris Pilatus 100K detector. Temperature control was achieved using a nitrogen-gas Cryostream controller (Oxford Cryosystems) allowing for a temperature control of about 0.1 ºC. Lindemann capillaries of diameter 0.6 mm were utilized. In the case of gels and xerogels, the materials were held in loops of 300 – 500 µm of diameter (MiTeGen). Monochromatic Cu-Kα radiation (λ = 1.5418 Å) was used. The exposure time was 2 minutes.

Circular dichroism spectra were recorded on a Jasco J-810 spectropolarimeter. For thin film sample preparation, a 100 µL $CHCl_3$ solution of the material was spin-coated onto a quartz plate (30 mg/mL at 1500rpm during 1 min), heated to the isotropic liquid, and slowly cooled to room temperature to promote proper mesophase formation. CD spectra were recorded at different rotation angles around the light beam showed the same trace and were averaged in order to compensate linear dichroism artifacts (**Figure SI16**). Experiments in the gel state at variable temperature were carried out using a Jasco CDF-426S sample holder with a 0.1 mm quartz cell. The preparation of the samples was carried out by introducing the gels into the measurement cuvettes, heating them to the sol state and cooling down slowly to room temperature.

The morphological characterization of gels was carried out by **transmission electron microscopy** (**TEM**) recorded using a TECNAI G2 20 (LMA, Universidad de Zaragoza) operating at 200 kV (accelerating voltage). The samples were prepared by depositing one drop of a dispersion on a carbon film copper grid, drying on aire and negatively stained with uranyl acetate prior to observation for better contrast. **Scanning electron microscopy** (**SEM**) recorded using an INSPECT-F50 (LMA, Universidad de Zaragoza) operating at 10 kV (accelerating voltage). The samples were prepared by depositing one drop of a dispersion on a glass slide and drying it on air. The surface of the samples was covered with a Pd coat. **Atomic force microscope** (**AFM**). Topographic images were obtained by means of a Multimode 8 microscope equipped with a Nanoscope V control unit from Bruker at a scan rate of 1.0–1.2 Hz, using Tapping mode. The data were collected using RTESPA-150 tips (nominal frequency of 150kHz, from Bruker) in air. **Cyclic voltammetry** experiments were performed using an Autolab PGSTAT204 potentiostat. The cyclic voltammograms were recorded in a deoxygenated solution of the compound (10^{-4} M) and tetrabutylammoniumhexafluorophosphate (0.1 M) in CH_2Cl_2 and in drop-cast films of the compounds from solution of CH_2Cl_2 in acetonitrile solution of tetrabutylammoniumhexafluorophosphate (0.1 M).

Synthesis and characterization

***N,N*-dibenzyl-4-nitroaniline (1)**

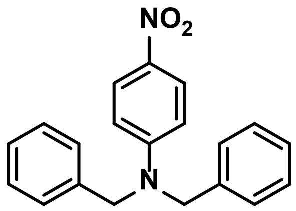

Potassium carbonate (181 mmol), sodium iodide (72.4 mmol) and benzyl bromide (181 mmol) were added to a solution of p-nitroaniline (36.2 mmol) in dry DMF (180 mL) under argon atmosphere. The resulted mixture was stirred at 120°C for 24 hours. Then DMF was evaporated under reduced pressure and the residue was redissolved in EtOAc (300 mL), washed with 1M HCl (3 x 300 mL) and brine (2 x 300 mL). The organic layer was dried with $MgSO_4$, filtered and the solvent was removed under reduced pressure. The crude product was recrystallized in ethanol two times obtaining a crystalline yellow solid. Yield: **52 %.**

^{1}H-NMR (400 MHz, $CDCl_3$): δ 8.10 - 8.04 (m, 2H, ArH), 7.42 - 7.27 (m, 6H, ArH), 7.24 - 7.17 (m, 4H, ArH), 6.73 - 6.67 (m, 2H, ArH), 4.76 (s, 4H, CH_2).

^{13}C-NMR (100 MHz, $CDCl_3$): δ 153.9, 138, 136.5, 129.2, 127.8, 126.4, 126.4, 111.2, 54.5.

IR (KBr, cm^{-1}): 1600 (NO_2), 1519 (C-C_{Ar}), 1487 (C-C_{Ar}), 1319 (NO_2), 1110 (C-N).

HRMS (ESI +): 341.12 $[M+Na]^+$, 659.2587 $[2M+Na]^+$.

N^1,N^1-dibenzylbenzene-1,4-diamine (2)

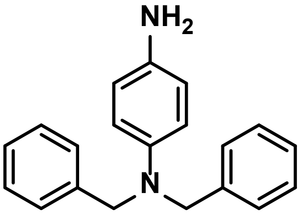

A suspension of *N,N*-dibenzyl-4-nitroaniline **(1)** (4.7 mmol) in MeOH (20 mL per mmol) was deoxygenated by bubbling Ar during 15 minutes. After this time, Pd/C (3 %wt) and a solution of $NaBH_4$ (4.7 mmol) and NaOH (2.35 mmol) in water (5 mL) were added dropwise. The reaction mixture was stirred during 30 minutes until the complete disappearance of the starting reagent is observed by TLC. $NaHCO_3$ (400 mL) resulting in the formation of a white emulsion. After this time, the reaction mixture was filtered through a celite® pad and the solvent was evaporated under reduced pressure. The obtained crude is redissolved in EtOAc (100 mL) and washed with distilled water (2 x 100 mL) and brine (1 x 100 mL). The organic layer was dried with $MgSO_4$, filtered and the solvent is evaporated under reduced pressure, giving rise to 1.35 g of a brown solid that is subsequently used without further purification. Yield: **quantitative.**

^{1}H-NMR (400 MHz, CD_2Cl_2): δ 7.34 - 7.26 (m, 8H, ArH), 7.25 - 7.20 (m, 2H, ArH), 6.65 - 6.60 (m, 2H, ArH), 6.56 - 6.51 (m, 2H, ArH), 4.50 (s, 4H, CH_2), 3.32 (s, 2H, NH_2).

^{13}C-NMR (100 MHz, CD_2Cl_2): δ 142.9, 140.1, 138.8, 128.9, 127.7, 127.2, 116.8, 116.4, 56.3.

IR (KBr, cm^{-1}): 3423, 3350 (NH_2), 3021 (C_{Ar}-H), 2913 (Csp^3-H), 2862 (Csp^3-H), 1601 (NH_2 δ), 1514 (C-C_{Ar}), 1492 (C-C_{Ar}).

HRMS (ESI +): 289.1642 $[M+H]^+$, 311.1466 $[M+Na]^+$.

1-(dodecyloxy)-4-iodobenzene (3)

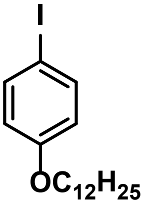

Potassium carbonate (95.6 mmol), potassium iodide (4.8 mmol) and 1-bromododecane (58.6) were added to a solution of 4-iodophenol (47.3 mmol) in butanone (100 mL) under argon atmosphere. The resulted mixture was stirred overnight under reflux. After cooling at room

temperature, the mixture was filtered through a celite® pad and the solvent was removed under reduced pressure. The crude was recrystallized in ethanol obtaining a white crystalline solid. Yield: **70 %.**

^{1}H-NMR (400 MHz, $CDCl_3$): δ 7.56 - 7.51 (m, 2H, ArH), 6.69 - 6.64 (m, 2H, ArH), 3.91 (t, J = 6.6 Hz, 2H, OCH_2), 1.81 - 1.71 (m, 2H, CH_2), 1.47 - 1.20 (m, 18H, CH_2), 0.88 (t, J = 6.9 Hz, 3H, CH_3).
^{13}C-NMR (100 MHz, $CDCl_3$): δ 159.2, 138.3, 117.1, 82.5, 68.3, 32.1, 29.8, 29.8, 29.7, 29.7, 29.50, 29.5, 29.3, 26.1, 22.8, 14.3.
IR (KBr, cm^{-1}): 2917 (Csp^3-H), 2849 (Csp^3-H), 1588 (C-C_{Ar}), 1489 (C-C_{Ar}), 1474 (C-C_{Ar}), 1464 (C-C_{Ar}), 1285 (C-O), 1249 (C-O).

N^1,N^1-dibenzyl-N^4,N^4-bis(4-(dodecyloxy)phenyl)benzene-1,4-diamine (4)

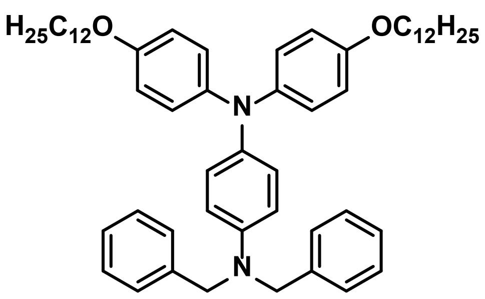

A suspension of N^1,N^1-dibenzylbenzene-1,4-diamine **(2)** (4.16 mmol), 1-(dodecyloxy)-4-iodobenzene **(3)** (12.1 mmol) and ^{t}BuOK (24.1 mmol) in dry toluene (30 mL) were prepared under argon atmosphere into a Schlenk. In a second Schlenk, $Pd_2(dba)_3$ and $(^tBu)_3P$ were dissolved in dry toluene (20 mL) under argon atmosphere. The solution of the second Schlenk were transferred to the first Schlenk with a cannula and the reaction mixture was stirred overnight at 90°C. After cooling at room temperature, the reaction mixture was diluted with EtOAc (200 mL), filtered through a celite® pad and washed with distilled water (2 x 200 mL) and brine (2 x 200 mL). The organic layer was dried with $MgSO_4$, filtered and the solvent was removed under reduced pressure. The crude was purified by flash chromatography using hexane/EtOAc (100:1). The product was obtained as a colorless oil.
Yield: **89 %**.

^{1}H-NMR (400 MHz, Acetone d_6): δ 7.36 - 7.28 (m, 8H, ArH), 7.27 - 7.19 (m, 2H, ArH), 6.91 - 6.74 (m, 10H, ArH), 6.73 - 6.66 (m, 2H, ArH), 4.65 (s, 4H, CH_2), 3.91 (t, J = 6.5 Hz, 4H, OCH_2), 1.78 - 1.69 (m, 4H, CH_2), 1.50 - 1.21 (m, 36H, CH_2), 0.88 (t, J = 6.8 Hz, 6H, CH_3).
^{13}C-NMR (100 MHz, Acetone d_6): δ 155.4, 146.1, 143.2, 140.4, 139.6, 129.5, 127.9, 127.8, 126.4, 125.2, 116.1, 114.9, 68.9, 55.8, 32.8, 30.5, 30.5, 30.5, 30.3, 30.2, 27, 23.5, 14.5.

IR (KBr, cm^{-1}): 3040 (C_{Ar}-H), 2925 (Csp^3-H), 2852 (Csp^3-H), 1604 (C-C_{Ar}), 1584 (C-C_{Ar}), 1502 (C-C_{Ar}), 1472 (C-C_{Ar}), 1455 (C-C_{Ar}), 1270 (C-O), 1237 (C-O).

HRMS (ESI +): 808.5855 $[M]^+$.

N,N-bis(4-dodecyloxyphenyl)-4-aminophenylaniline (5)

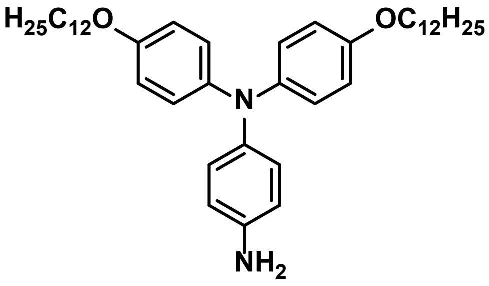

Pd/C 10% (0.37 g) was added to a solution of compound **(4)** (3.7 mmol) in ethyl acetate. The mixture was stirred at room temperature for 6 hours under H_2 atmosphere. Then the catalyst was filtered off through a celite pad and the solvent was removed under reduced pressure. The crude was purified by flash chromatography using hexane/EtOAc (9:1). The product was obtained as a waxed purple solid. Yield: **90 %.**

^{1}H-NMR (400 MHz, CD_2Cl_2): δ 6.92 - 6.86 (m, 4H, ArH), 6.85 - 6.80 (m, 2H, ArH), 6.77 - 6.71 (m, 4H, ArH), 6.61 - 6.55 (m, 2H, ArH), 3.89 (t, *J* = 6.6 Hz, 4H, OCH_2), 3.57 (s, 2H, NH_2), 1.79 - 1.69 (m, 4H, CH_2), 1.48 - 1.23 (m, 36H, CH_2), 0.88 (t, *J* = 6.9 Hz, 6H, CH_3).

^{13}C-NMR (100 MHz, CD_2Cl_2): δ 154.9, 142.9, 142.7, 140.6, 126.4, 124.7, 116.4, 115.6, 68.9, 32.5, 30.3, 30.2, 30.2, 30.2, 30, 30, 29.9, 26.6, 23.3, 14.5.

IR (KBr, cm^{-1}): 3453, 3370 (NH_2), 3040 (C_{Ar}-H), 2922 (Csp^3-H), 2851 (Csp^3-H), 1625 (C-C_{Ar}), 1501 (C-C_{Ar}), 1470 (C-C_{Ar}), 1263 (C-O), 1235 (C-O).

HRMS (ESI +): 628.4923 $[M]^+$.

General procedure of amidation

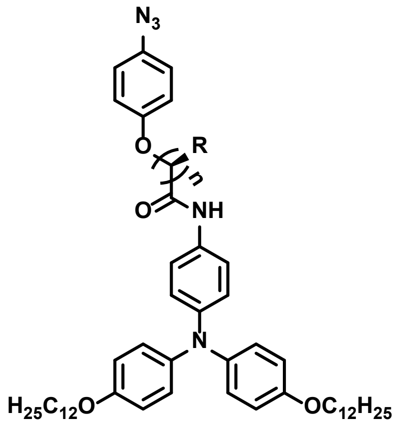

N_3-2CTPA, R = H, n = 1
N_3-2C*TPA, R = CH_3, n = 1
N_3-3CTPA, R = H, n = 2

EDC (1.5 mmol) was added to a solution of azide containig carboxylic acid (1.2 mmol) in dry THF (10 mL/mmol) under Ar atmosphere. The reaction mixture was stirring 30 min at room temperature. Then N,N-bis(4-dodecyloxyphenyl)-4-aminophenylaniline **(5)** (1 mmol) was added and the reaction mixture was stirring overnight at room temperature. Solvent was removed under reduced pressure and the crude residue was purified by flash chromatography using hexane/EtOAc. (Caution: storage in the freezer to avoid azide decomposition.

N_3-2CTPA

The crude was purified by flash chromatography using hexane/EtOAc (9:1) and recrystalized in EtOH giving a white powder. Yield: **80 %.**

^{1}H-NMR (400 MHz, CD_2Cl_2): δ 8.13 (s, 1H, NH), 7.39 - 7.33 (m, 2H, ArH), 7.05 - 6.96 (m, 8H, ArH), 6.92 - 6.87 (m, 2H, ArH), 6.83 - 6.77 (m, 4H, ArH), 4.56 (s, 2H, OCH_2) 3.92 (t, *J* = 6.6 Hz, 4H, OCH_2), 1.80 - 1.70 (m, 4H, CH_2), 1.50 - 1.20 (m, 36H, CH_2), 0.88 (t, *J* = 6.9 Hz, 6H, CH_3).

^{13}C-NMR (100 MHz, CD_2Cl_2): δ 166.1, 156.0, 155.1, 146.6, 141.4, 134.7, 130.5, 126.8, 121.9, 121.8, 120.9, 116.8, 115.8, 68.9, 68.6, 32.5, 30.3, 30.2, 30.2, 30.2, 30, 29.9, 26.6, 23.3, 14.5.

IR (KBr, cm^{-1}): 3324 (NH), 3044 (C_{Ar}-H), 2921 (Csp^3-H), 2851 (Csp^3-H), 2117 (N_3), 1662 (C=O), 1606 (C-C_{Ar}), 1505 (C-C_{Ar}), 1473 (C-C_{Ar}), 1238 (C-O).

HRMS (ESI +): 803.5338 $[M]^+$.

N_3-2C*TPA

The crude was purified by flash chromatography using hexane/EtOAc (9:1) and recrystalized in EtOH giving a white crystalline powder. Yield: **83 %.**

^{1}H-NMR (400 MHz, CD_2Cl_2): δ 8.04 (s, 1H, NH), 7.35 - 7.27 (m, 2H, ArH), 7.04 - 6.93 (m, 8H, ArH), 6.90 - 6.84 (m, 2H, ArH), 6.83 - 6.76 (m, 4H, ArH), 4.71 (q, *J* =6.7 Hz, 1H, OCH) 3.91 (t, *J* = 6.6 Hz, 4H, OCH_2), 1.80 - 1.70 (m, 4H, CH_2), 1.61 (d, *J* =6.7 Hz, 3H, CH_3), 1.50 - 1.20 (m, 36H, CH_2), 0.88 (t, *J* = 6.8 Hz, 6H, CH_3).

^{13}C-NMR (100 MHz, CD_2Cl_2): δ 170.1, 156.0, 154.8, 146.4, 141.4, 134.6, 130.8, 126.7, 121.9, 121.7, 120.9, 117.8, 115.8, 76.6, 68.9, 32.5, 30.3, 30.2, 30.2, 30.2, 30, 29.9, 26.6, 23.3, 19.0, 14.5.

IR (KBr, cm^{-1}): 3260 (NH), 3043 (C_{Ar}-H), 2921 (Csp^3-H), 2851 (Csp^3-H), 2110 (N_3), 1672 (C=O), 1603 (C-C_{Ar}), 1505 (C-C_{Ar}), 1476 (C-C_{Ar}), 1262 (C-O), 1239 (C-O).

HRMS (ESI +): 817.5517 $[M]^+$.

N_3-3CTPA

The crude was purified by flash chromatography using hexane/EtOAc (8:2) and and recrystalized in EtOH giving a white crystalline powder. Yield: **85 %.**

^{1}H-NMR (400 MHz, CD_2Cl_2): δ 7.50 (s, 1H, NH), 7.33 - 7.26 (m, 2H, ArH), 7.00 - 6.91 (m, 8H, ArH), 6.90 - 6.83 (m, 2H, ArH), 6.82 - 6.76 (m, 4H, ArH), 4.29 (t, *J* = 5.9 Hz, 2H, OCH_2), 3.91 (t, *J* = 6.5 Hz, 4H, OCH_2), 2.76 (t, *J* = 5.9 Hz, 2H, CH_2), 1.79 - 1.70 (m, 4H, CH_2), 1.49 - 1.18 (m, 36H, CH_2), 0.88 (t, J=6.9 Hz, 6H, CH_3).

^{13}C-NMR (100 MHz, $CDCl_3$): δ 168.7, 156.4, 155.9, 146.1, 141.5, 133.5, 131.7, 126.6, 122.1, 121.7, 120.6, 116.5, 115.8, 68.9, 65.3, 38.0, 32.5, 30.3, 30.2, 30.2, 30.2, 30.0, 29.9, 26.6, 23.3, 14.5.

IR (KBr, cm^{-1}): 3307 (NH), 3045 (C_{Ar}-H), 2920 (Csp^3-H), 2850 (Csp^3-H), 2107 (N_3), 1650 (C=O), 1594 (C-C_{Ar}), 1505 (C-C_{Ar}), 1475 (C-C_{Ar}), 1270 (C-O), 1240 (C-O).

HRMS (ESI +): 817.5509 $[M]^+$, 840.5418 $[M+Na]^+$.

General procedure of azide-alkyne cycloaddition

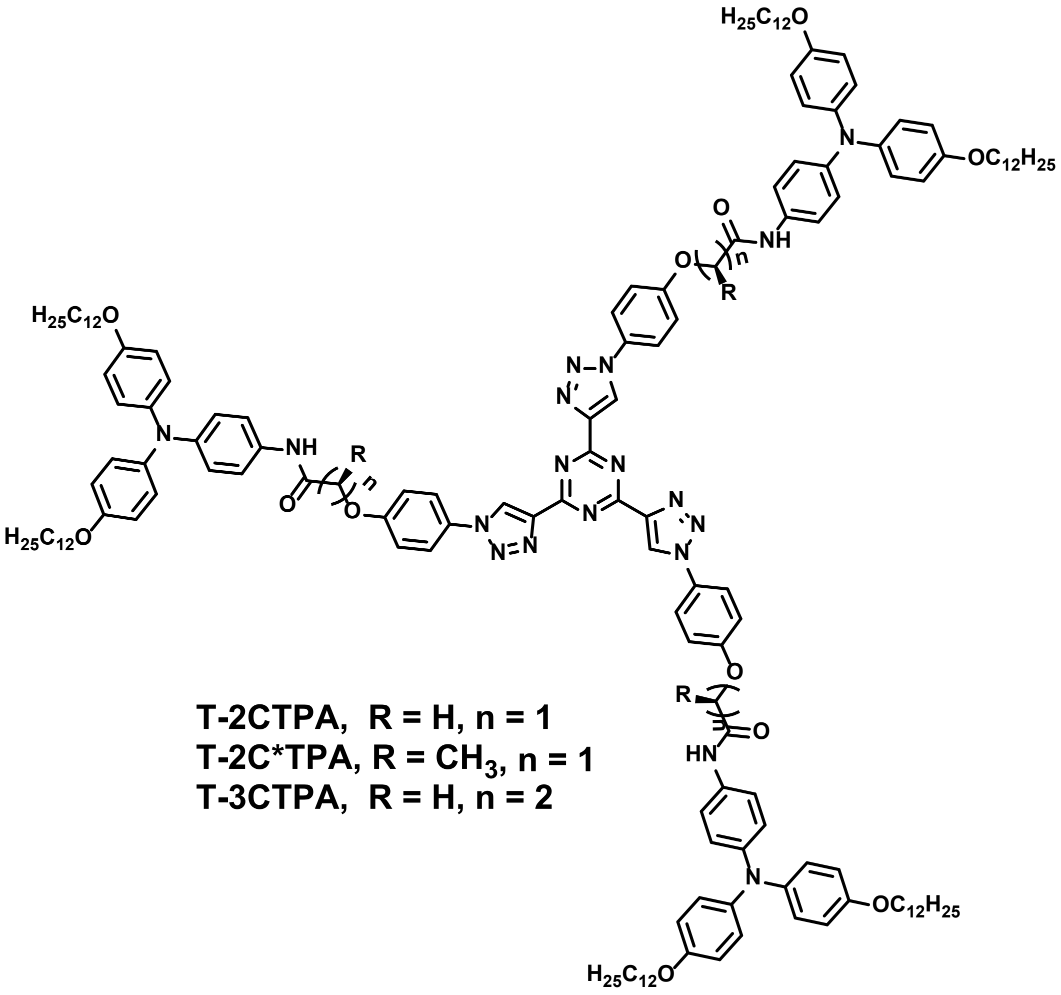

T-2CTPA, R = H, n = 1
T-2C*TPA, R = CH_3, n = 1
T-3CTPA, R = H, n = 2

$CuSO_4 \cdot 5H_2O$ (0.15 mmol), aryl azide (3.3 mmol), sodium ascorbate (0.3 mmol) and TBTA (0.15 mmol) were added to a mixture of H_2O/ ^tBuOH /CH_2Cl_2 (1/2/8, 33 mL) under Ar atmosphere and vigorous stirring. After 15 min, 2,4,6-tris(trimethylsilylethynyl)-1,3,5-triazine (1 mmol) was added and the solution turned red immediately. Then, KF solution (3.3 mmol in 1 mL of water) was added dropwise over 2 hours and the mixture was stirred overnight at room temperature in the darkness. The mixture was diluted with CH_2Cl_2 (30 mL) and the organic phase was washed with aqueous 0.1M EDTA-Na_2 solution (3 x 30 mL) and brine (3 x 30 mL). The organic layer was dried with $MgSO_4$, filtrated and the solvent was removed under reduced pressure.

T-2CTPA

The crude was purified by flash chromatography using DCM/MeOH (9:1) and the product was obtained as a yellow-brown solid. Yield: **61 %.**

^{1}H-NMR (400 MHz, $CDCl_3$): δ 9.11 (s, 3H, triazole), 8.22 (s, 3H, NH) 7.90 - 7.79 (m, 6H, ArH), 7.46 - 7.36 (m, 6H, ArH), 7.22 - 7.14 (m, 6H, ArH), 7.05 - 6.98 (m, 12H, ArH), 6.97 - 6.91 (m, 6H, ArH),

6.85 - 6.75 (m, 12H, ArH), 4.69 (s, 6H, OCH_2) 3.93 (t, *J* = 6.4 Hz, 12H, OCH_2), 1.84 - 1.71 (m, 12H, CH_2), 1.52 - 1.18 (m, 108H, CH_2), 0.89 (t, *J* = 6.5 Hz, 18H, CH_3).

^{13}C-NMR (100 MHz, $CDCl_3$): δ 166.8, 165.3, 157.8, 155.5, 146.3, 146, 140.9, 131.3, 129.7, 126.3, 125.9, 122.6, 121.7, 121.6, 116.1, 115.4, 68.4, 68.1, 32.1, 29.8, 29.8, 29.8, 29.6, 29.5, 29.5, 26.2, 22.8, 14.3.

IR (KBr, cm^{-1}): 3406 (NH free), 3305 (NH associated), 3041 (C_{Ar}-H), 2924 (Csp^3-H), 2853 (Csp^3-H), 1680 (C=O), 1595 (C-C_{Ar}), 1564 (NH δ), 1503 (C-C_{Ar}), 1467 (C-C_{Ar}), 1237 (C-O).

MS (MALDI+, dithranol): 2564.58 $[M]^+$.

EA calculated (%) for $C_{159}H_{210}N_{18}O_{12}$: C 74.44, H 8.25, N 9.83; found: C 74.76, H 8.15, N 9.61.

T-2C*TPA

The crude was purified by flash chromatography using DCM/MeOH (95:5) and the product was obtained as a yellow-brown solid. Yield: **52 %.**

^{1}H-NMR (400 MHz, $CDCl_3$): δ 9.10 (s, 3H, triazole), 8.11 (s, 3H, NH) 7.85 - 7.77 (m, 6H, ArH), 7.42 - 7.33 (m, 6H, ArH), 7.20 - 7.11 (m, 6H, ArH), 7.03 - 6.95 (m, 12H, ArH), 6.94 - 6.88 (m, 6H, ArH), 6.83 - 6.74 (m, 12H, ArH), 4.86 (q, *J* = 6.5 Hz, 3H, OCH_2), 3.90 (t, *J* = 6.4 Hz, 12H, OCH_2), 1.80 - 1.68 (m, 21H, $CH_2 + CH_3$), 1.50 - 1.20 (m, 108H, CH_2), 0.87 (t, *J* = 6.4 Hz, 18H, CH_3).

^{13}C-NMR (100 MHz, $CDCl_3$): δ 169.3, 166.9, 157.6, 155.5, 146.2, 146, 141, 131.3, 130, 126.2, 126, 122.8, 121.8, 121.4, 117, 115.4, 76.2, 68.5, 32.1, 29.8, 29.8, 29.7, 29.7, 29.6, 29.5, 29.5, 26.2, 22.8, 14.2.

IR (KBr, cm^{-1}): 3406 (NH free), 3305 (NH associated), 3042 (C_{Ar}-H), 2923 (Csp^3-H), 2852 (Csp^3-H), 1680 (C=O), 1599 (C-C_{Ar}), 1567 (NH δ), 1505 (C-C_{Ar}), 1469 (C-C_{Ar}), 1238 (C-O).

MS (MALDI+, dithranol): 2606.43 $[M]^+$.

EA calculated (%) for $C_{162}H_{216}N_{18}O_{12}$: C 74.62, H 8.35, N 9.67; found: C 74.56, H 8.44, N 9.60.

T-3CTPA

The crude was purified by flash chromatography using DCM/MeOH (9:1) and the product was obtained as a brown solid. Yield: **55 %.**

^{1}H-NMR (400 MHz, tetrahydrofuran-d_8): δ 9.43 (s, 3H, triazole), 9.07 (s, 3H, NH), 8.04 - 7.95 (m, 6H, ArH), 7.53 - 7.42 (m, 6H, ArH), 7.22 - 7.11 (m, 6H, ArH), 6.98 - 6.89 (m, 12H, ArH), 6.88 - 6.82 (m, 6H, ArH), 6.81 - 6.73 (m, 12H, ArH), 4.42 (t, *J* = 6.3 Hz, 6H, OCH_2), 3.91 (t, *J* = 6.4 Hz, 12H, OCH_2), 2.79 (t, *J* = 6.3 Hz, 6H, CH_2), 1.50 - 1.20 (m, 108H, CH_2), 0.89 (t, *J* = 6.8 Hz, 18H, CH_3).

^{13}C-NMR (100 MHz, tetrahydrofuran-d_8): δ 168.5, 168.1, 160.4, 156.3, 145.6, 142.5, 134.5, 131.6, 126.8, 123.1, 122.7, 121.1, 116.3, 68.9, 65.8, 37.7, 33, 30.8, 30.8, 30.6, 30.6, 30.5, 27.3, 23.7, 14.6.

IR (KBr, cm^{-1}): 3295 (NH associated), 3042 (C_{Ar}-H), 2923 (Csp^3-H), 2852 (Csp^3-H), 1660 (C=O), 1602 (C-C_{Ar}), 1567 (NH δ), 1504 (C-C_{Ar}), 1469 (C-C_{Ar}), 1239 (C-O).

MS (MALDI+, dithranol): 2606.70 [M]$^+$.

EA calculated (%) for $C_{162}H_{216}N_{18}O_{12}$: C 74.62, H 8.35, N 9.67; found: C 74.83, H 8.54, N 9.45.

Infrared spectra

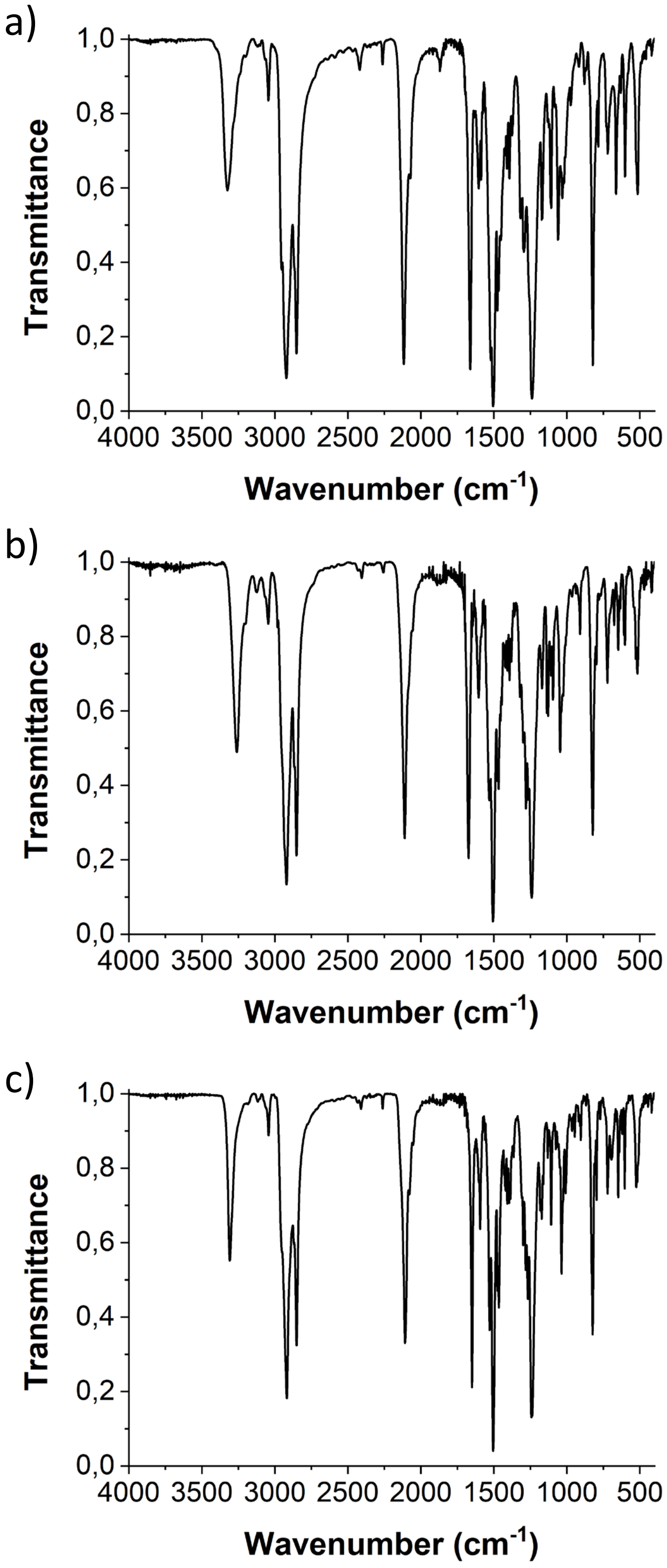

Figure S1. Infrared spectra of compounds **N_3-2CTPA** (a) **N_3-2C*TPA** (b) **N_3-3CTPA** (c) recorded on KBr pellets at room temperature.

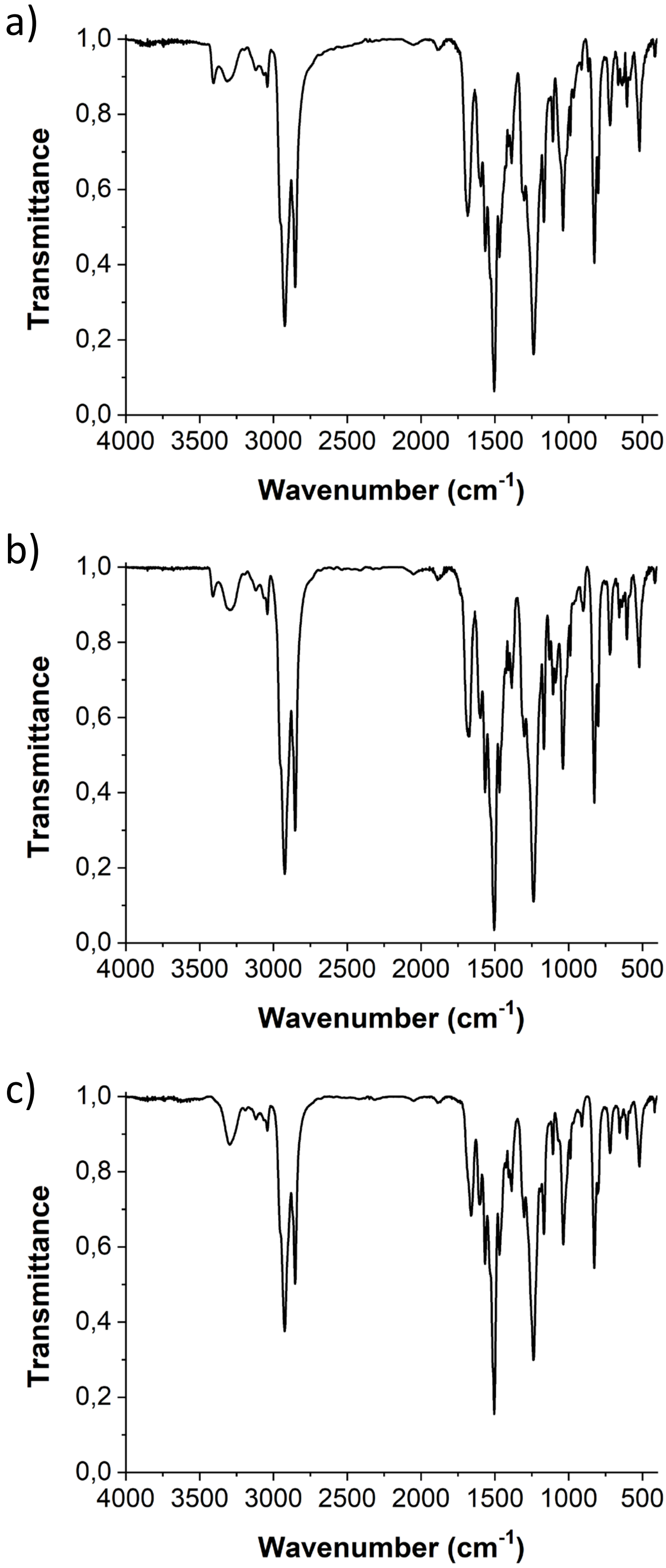

Figure S2. Infrared spectra of compounds **T-2CTPA** (a), **T-2C*TPA** (b), **T-3CTPA** (c) recorded on KBr pellets at room temperature.

^{1}H NMR and ^{13}C NMR spectra

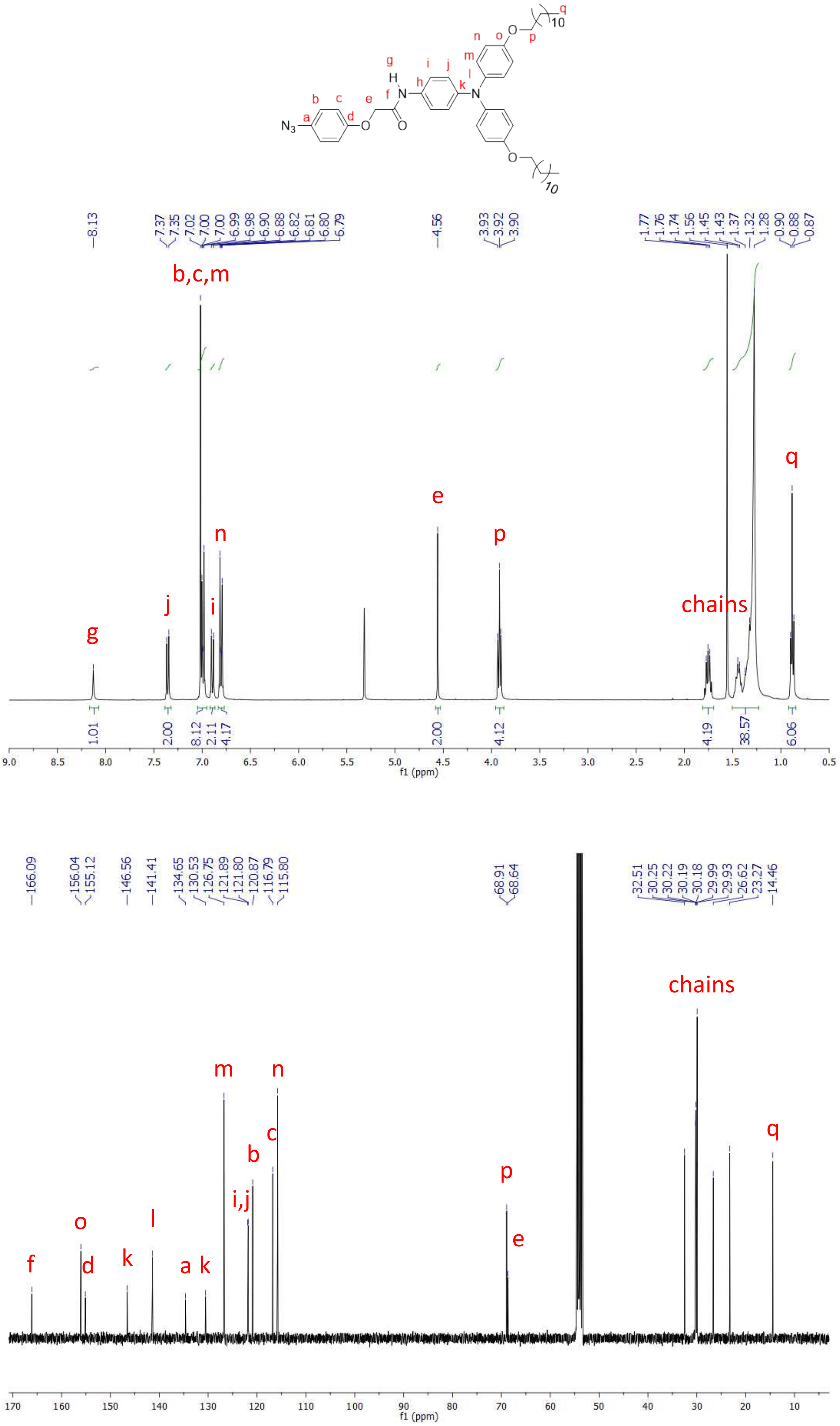

Figure S3. ^{1}H-NMR and ^{13}C-NMR spectra of compound **N_3-2CTPA** in CD_2Cl_2.

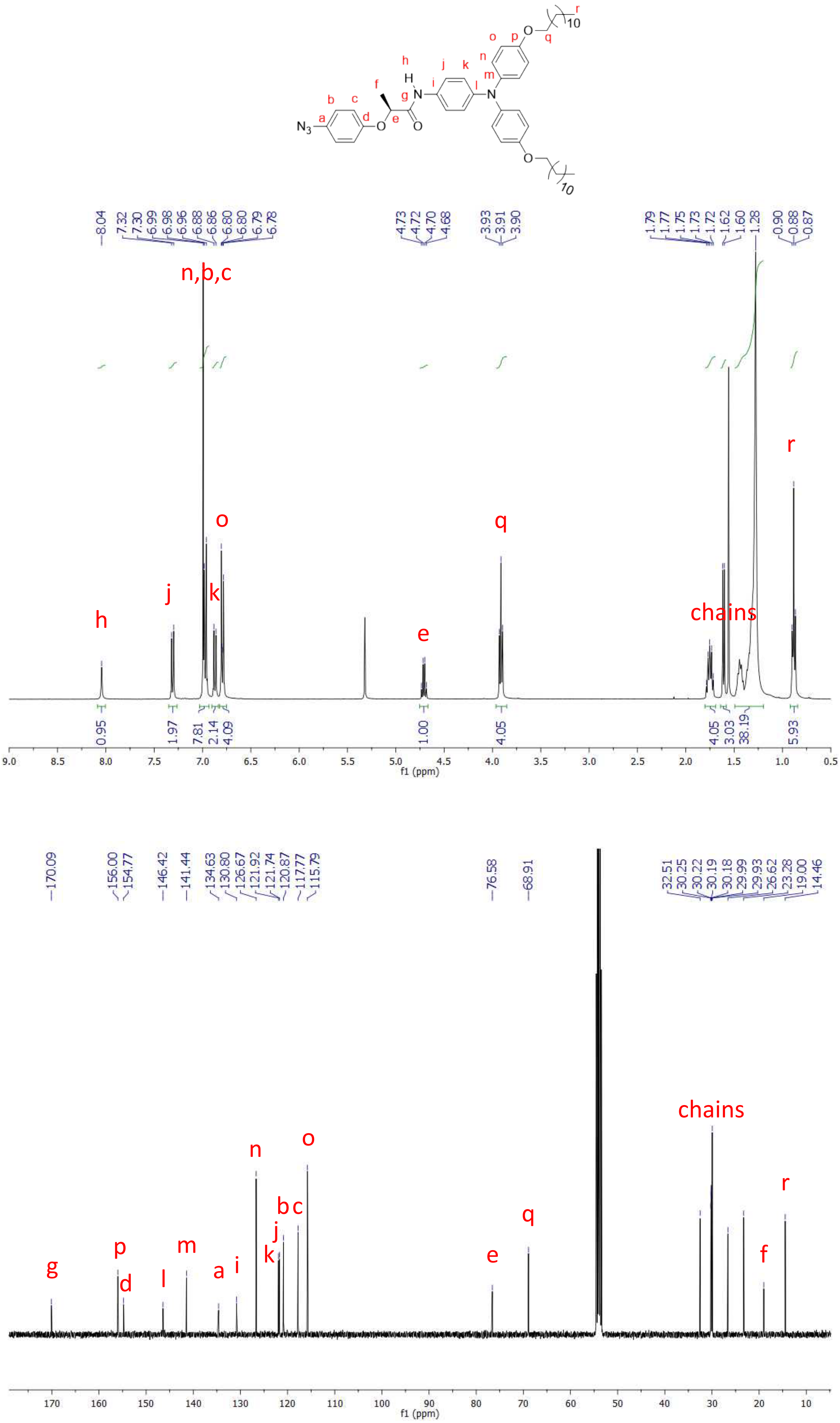

Figure S4. ^{1}H-NMR and ^{13}C-NMR spectra of compound **N_3-2C*TPA** in CD_2Cl_2.

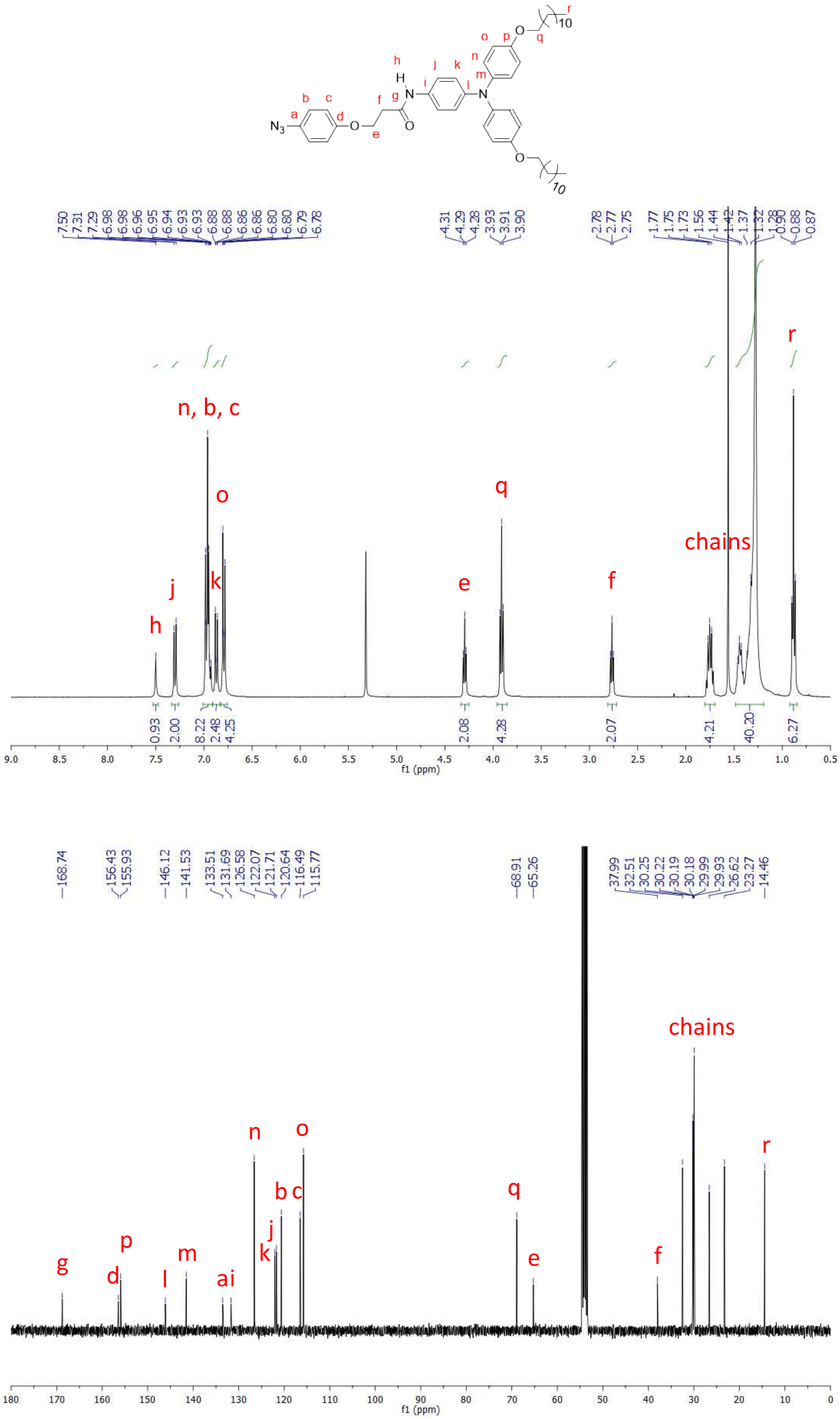

Figure S5. ^{1}H-NMR and ^{13}C-NMR spectra of compound **N_3-3CTPA** in CD_2Cl_2.

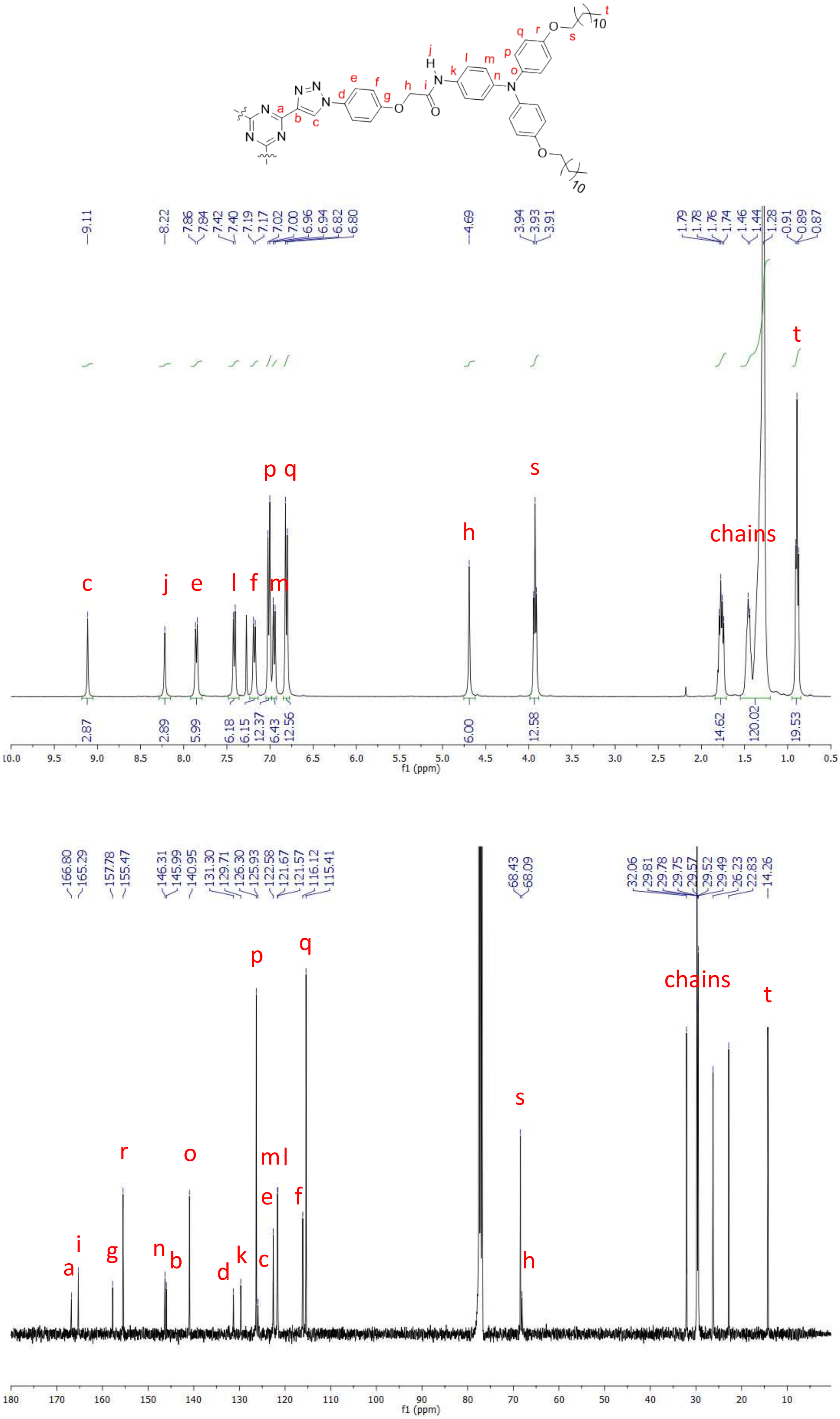

Figure S6. ^{1}H-NMR and ^{13}C-NMR spectra of compound **T-2CTPA** in $CDCl_3$.

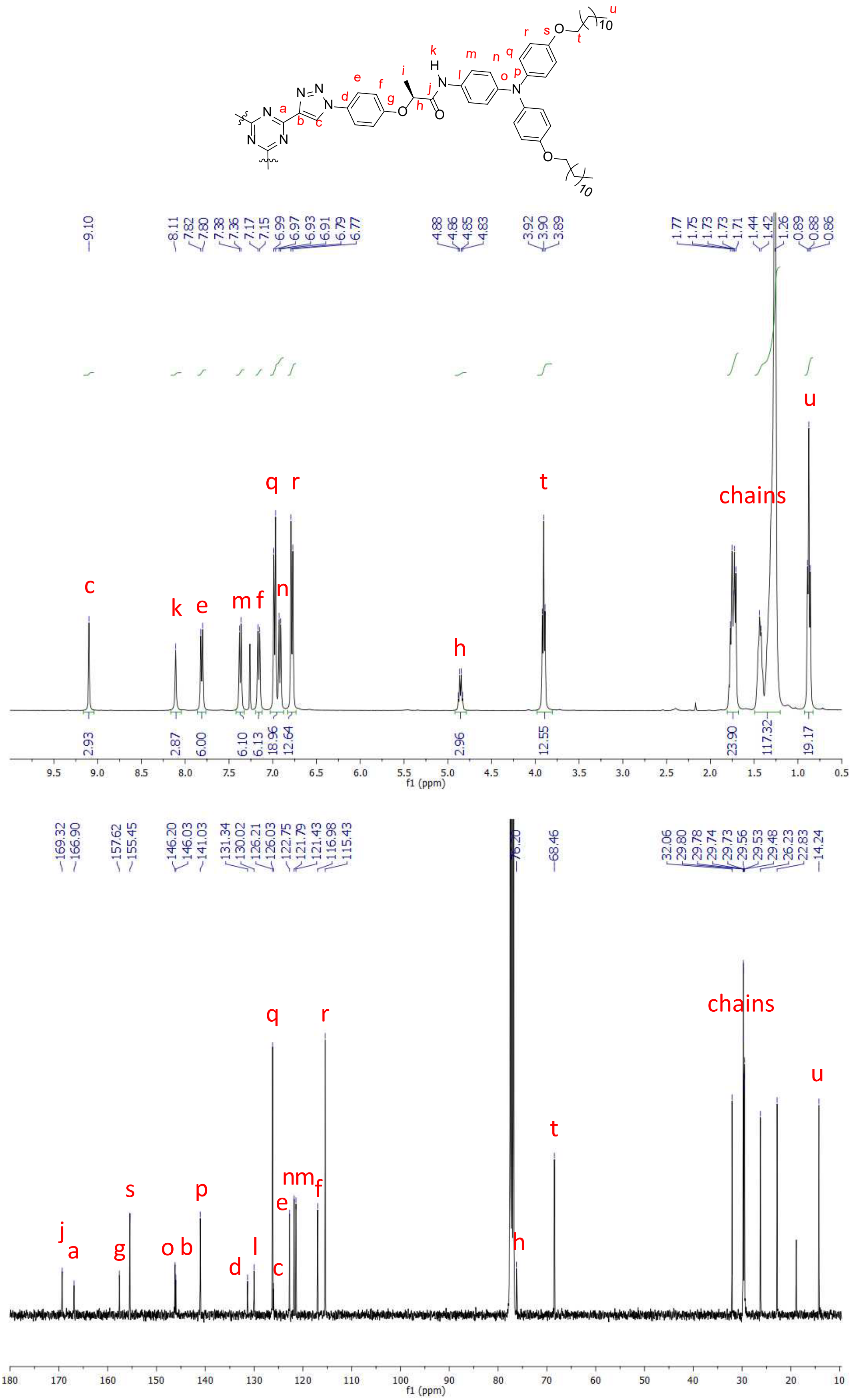

Figure S7. ^{1}H-NMR and ^{13}C-NMR spectra of compound **T-2C*TPA** in $CDCl_3$.

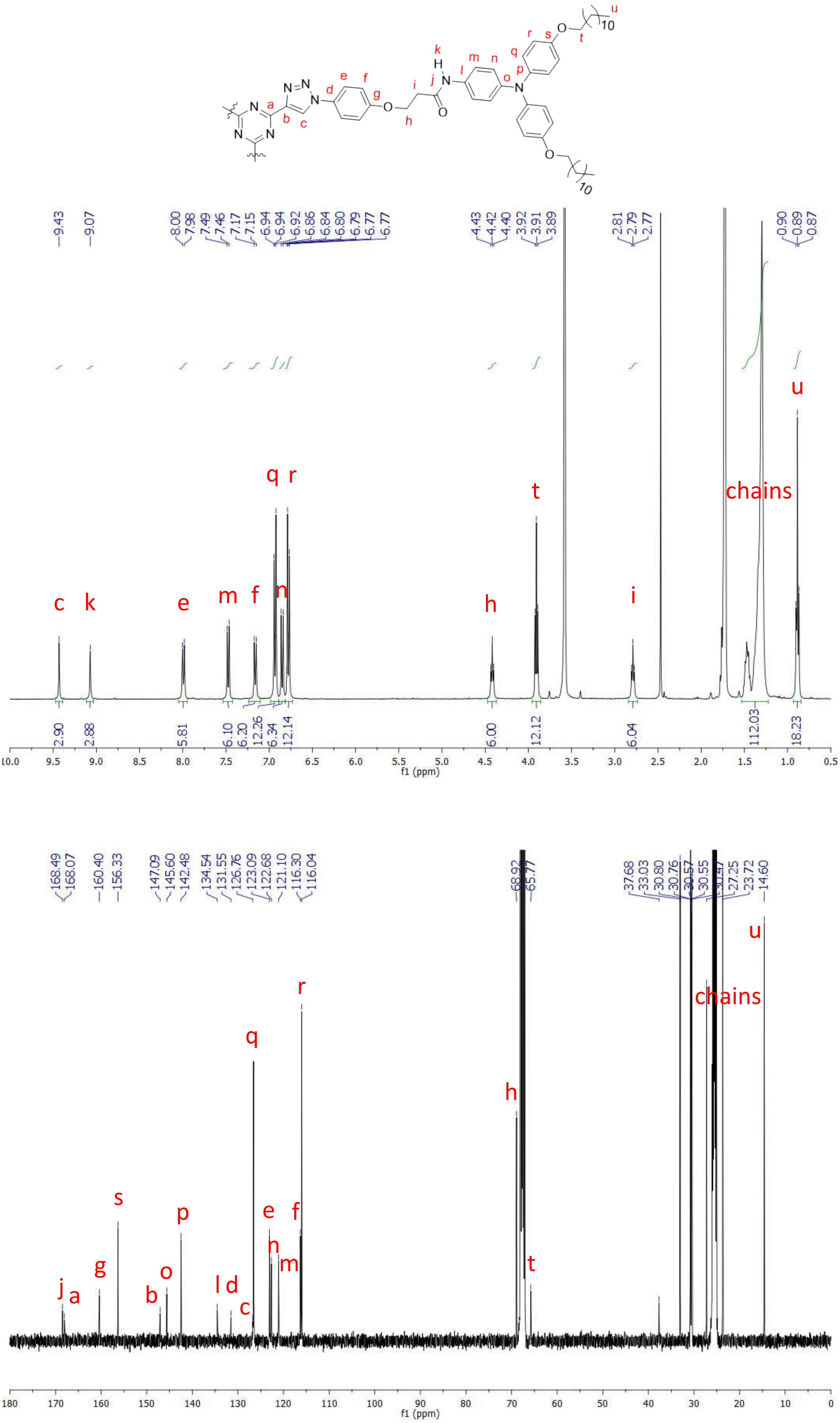

Figure S8. ^{1}H-NMR and ^{13}C-NMR spectra of compound **T-3CTPA** in $CDCl_3$.

Liquid Crystalline Properties

DSC thermograms

Due to the high clearing temperatures, compounds **T-2CTPA** and **T-3CTPA** started to decompose when the isotropic liquid state was reached. In order to avoid the decomposition and observe the intermediate transitions correctly, a first heating-cooling cycle 50 °C below the transition to the isotropic liquid was first recorded. The scans were performed at 20 °C/min to avoid the decomposition as much as possible.

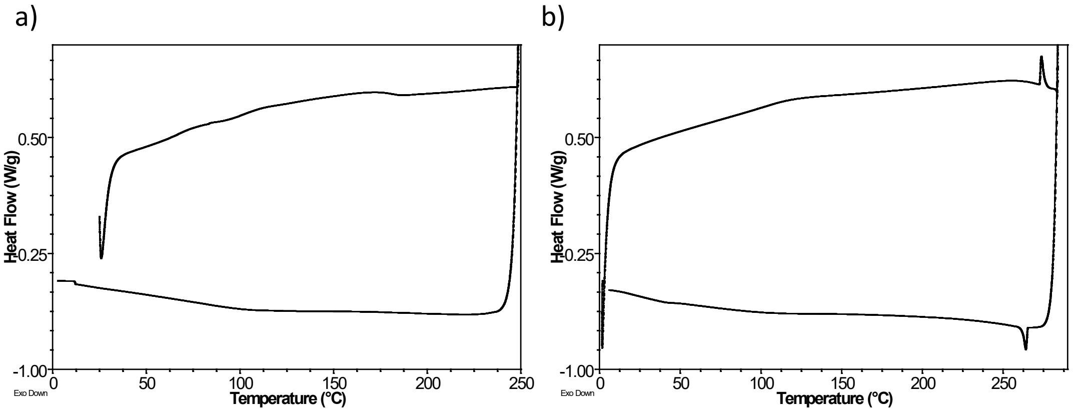

Figure S9. DSC thermograms corresponding to the first (a) and second (b) cycle of compound **T-2CTPA** at 20 °C/min.

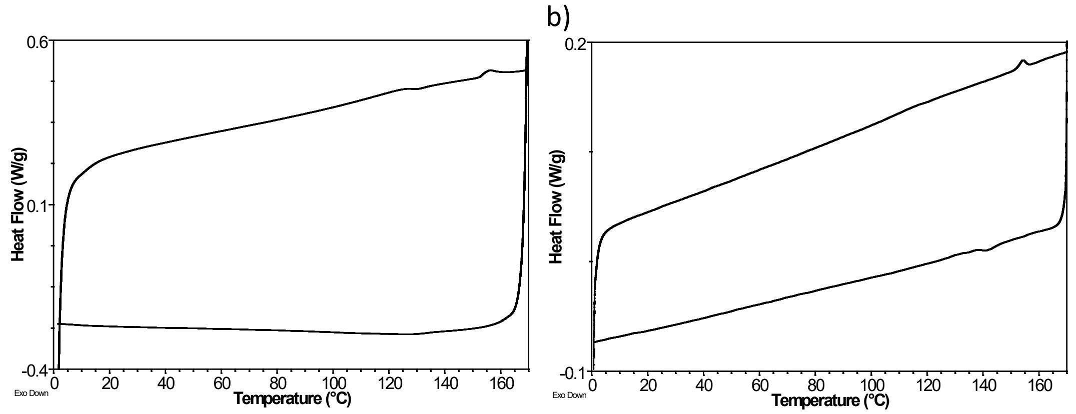

Figure S10. DSC thermograms corresponding to the first cycle at 10 °C/min (a) and second cycle at 2 °C/min (b) of compound **T-2C*TPA**.

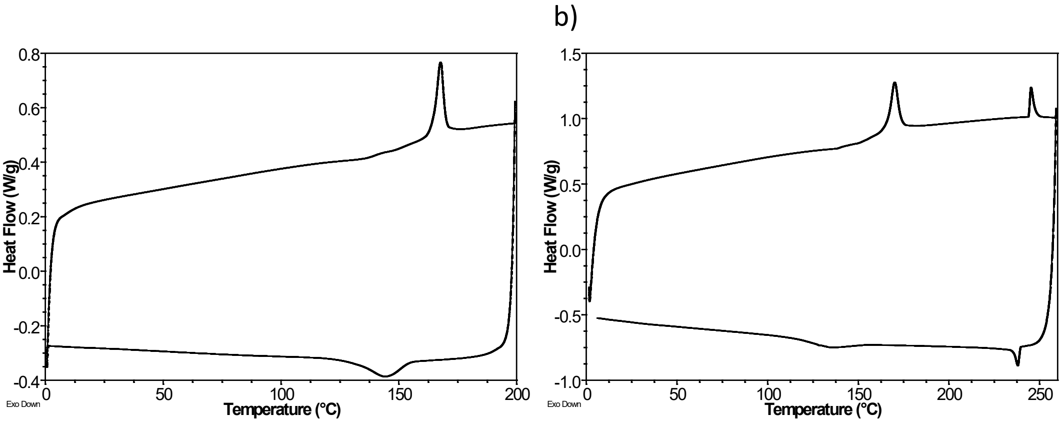

Figure S11. DSC thermograms corresponding to the first (a) and second cycle (b) of compound **T-3CTPA** at 20 °C/min.

X-Ray Diffraction

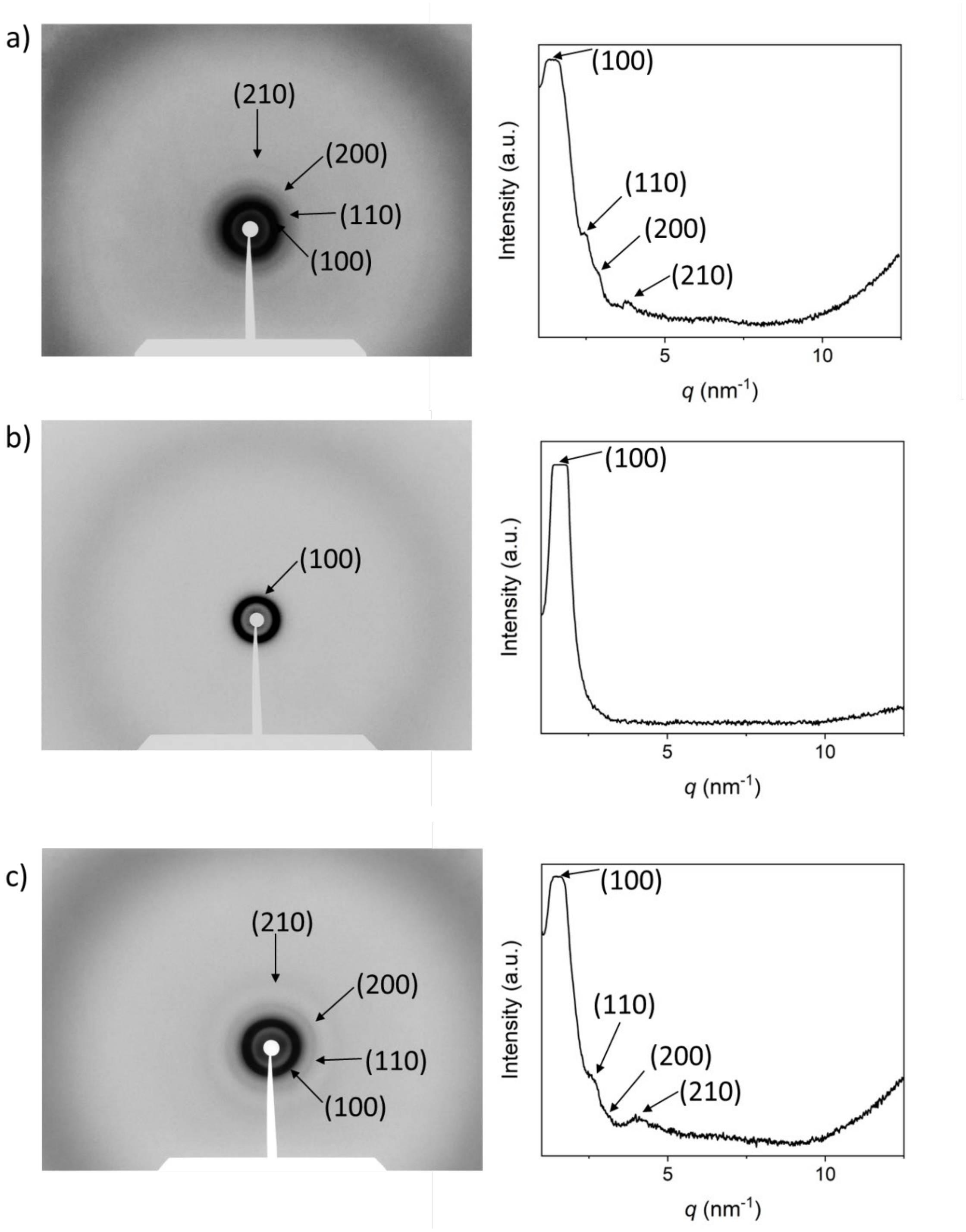

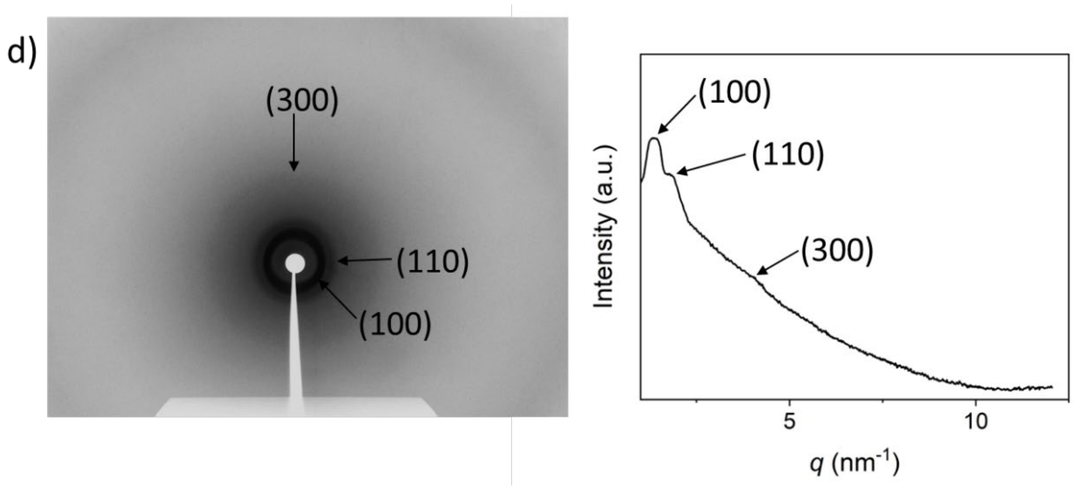

Figure S12. XRD patterns and their respective 1D plot of the compounds: (a) **T-2CTPA**, r.t., (b) **T-2C*TPA**, r.t.**,** (c) **T-3CTPA**, rt, and (d) **T-3CTPA**, 170 °C.

Density estimation

The density of the materials was calculated experimentally by buoyancy method and theoretically with the lattice parameters obtained by XRD.

Buoyancy method was carried out by suspending materials in 1 mL of 1-bromohexane (ρ = 1.176 g/mL) and adding portions of 20 μL of hexane (ρ = 0.661 g/mL) progressively until the material stopped floating. Finally, the density of the mixture was calculated with the mass and the volume of the mixture. For compounds **T-2CTPA** and **T-3CTPA**, density values of 1.1 g/cm^3 were obtained while for compound **T-2C*TPA** a density of 1 g/cm^3 was measured.

Theoretical calculations were carried out with the density equation $\rho = (M \cdot Z)/(N_A . V)$, where the volume of the unit cell can be calculated with the equation $V = a^2 \cdot \sqrt{3}/2 \cdot c$, considering Z = 1 and 2.

T-2CTPA

ρ = (*1 molecule x* 2565.54 g·mol^{-1} x 10^{24} Å^3·cm^{-3}) / ((*49.5 Å*)^2*x* √3/2 x 3.4 Å x *6.022·10*^{23} *mol*$^{-1}$) = 0.59 *g·cm*$^{-3}$

ρ = (*2 molecules x* 2565.54 g·mol^{-1} x 10^{24} Å^3·cm^{-3}) / ((*49.5 Å*)^2*x* √3/2 x 3.4 Å x *6.022·10*^{23} *mol*$^{-1}$) = 1.18 *g·cm*$^{-3}$

T-2C*TPA

ρ = (*1 molecule x* 2607.62 g·mol^{-1} x 10^{24} Å^3·cm^{-3}) / ((*40.6 Å*)^2*x* √3/2 x 3.4 Å x *6.022·10*^{23} *mol*$^{-1}$) = 0.89 *g·cm*$^{-3}$

ρ = (*2 molecules x* 2607.62 g·mol^{-1} x 10^{24} Å^3·cm^{-3}) / ((*40.6 Å*)^2*x* √3/2 x 3.4 Å x *6.022·10*^{23} *mol*$^{-1}$) = 1.78 *g·cm*$^{-3}$

T-3C

ρ = (*1 molecule x* 2607.62 g·mol^{-1} x 10^{24} Å^3·cm^{-3}) / (*(47.5 Å)^{2}x* √3/2 x 3.4 Å x *6.022·10^{23} mol^{-1})* = 0.65 *g·cm^{-3}*

ρ = (*2 molecules x* 2607.62 g·mol^{-1} x 10^{24} Å^3·cm^{-3}) / (*(47.5 Å)^{2}x* √3/2 x 3.4 Å x *6.022·10^{23} mol^{-1})* = 1.30 *g·cm^{-3}*

Calculation of Z

The number of molecules per unit cell (Z) in the Col_h mesophase was estimated with the density equation $\rho = (M \cdot Z)/(N_A . V)$, where the volume of the unit cell can be calculated with the equation $V = a^2 \cdot \surd 3/2 \cdot c$ and therefore, the first equation can be restructured as $Z = (\rho \cdot a^2 \cdot \surd 3/2 \cdot c \cdot N_A)/M$.

T-2CTPA

Z = (*1.1 g·cm^{-3} x (49.5 Å)^{2}x* √3/2 x 3.4 Å x *6.022·10^{23} mol^{-1})* / (2565.54 g·mol^{-1} x 10^{24} Å^3·cm^{-3}) = 1.9 molecules

T-2C*TPA

Z = (*1 g·cm^{-3} x (40.6 Å)^{2}x* √3/2 x 3.4 Å x *6.022·10^{23} mol^{-1})* / (2607.62 g·mol^{-1} x 10^{24} Å^3·cm^{-3}) = 1.1 molecules

T-3C

Z = (*1.1 g·cm^{-3} x (47.5 Å)^{2}x* √3/2 x 3.4 Å x *6.022·10^{23} mol^{-1})* / (2607.62 g·mol^{-1} x 10^{24} Å^3·cm^{-3}) = 1.7 molecules

FTIR studies at variable temperature

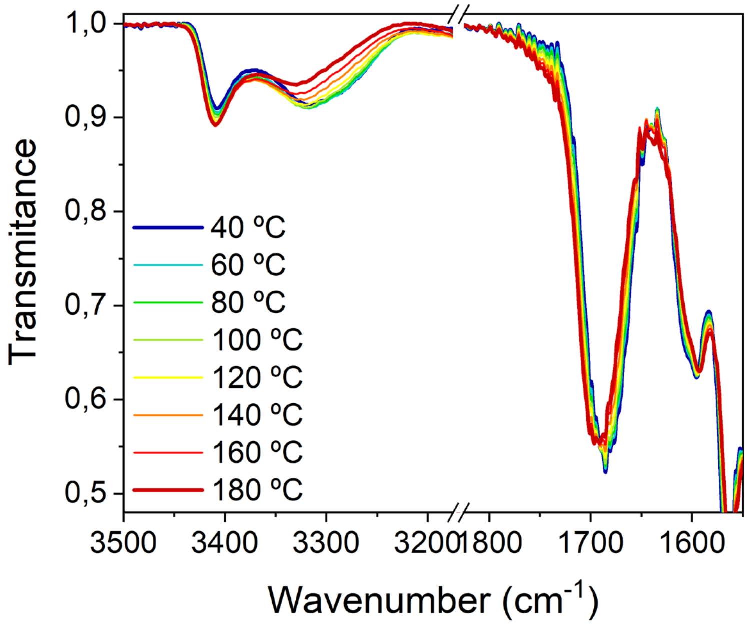

Figure SI13. Infrared spectra of compound **T-2CTPA** at variable temperature.

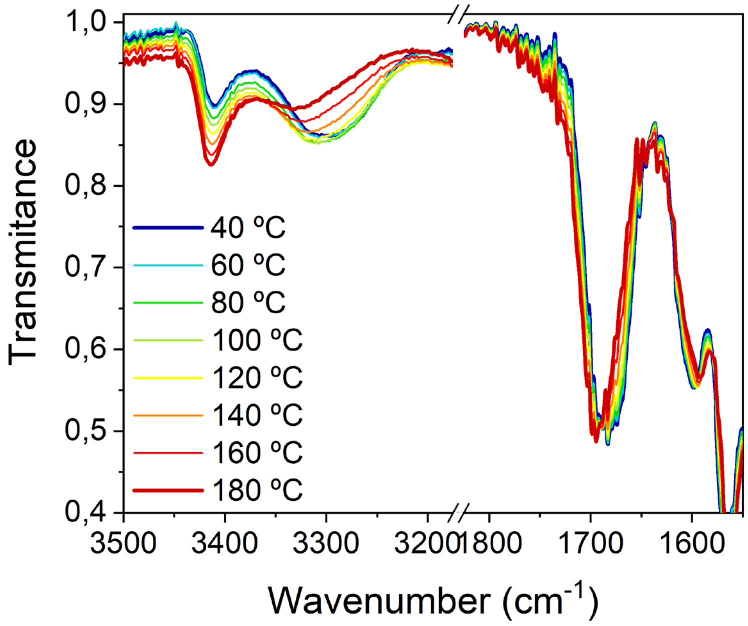

Figure SI14. Infrared spectra of compound **T-2C*TPA** at variable temperature.

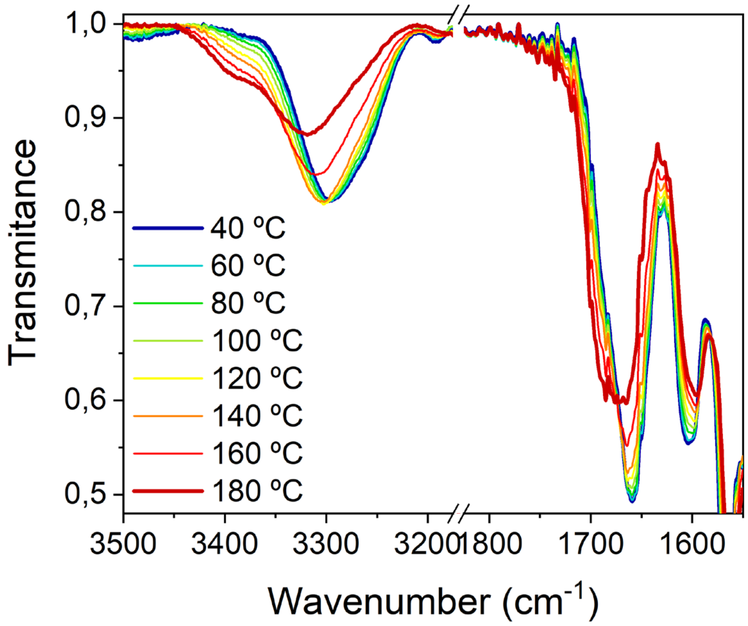

Figure SI15. Infrared spectra of compound **T-3CTPA** at variable temperature.

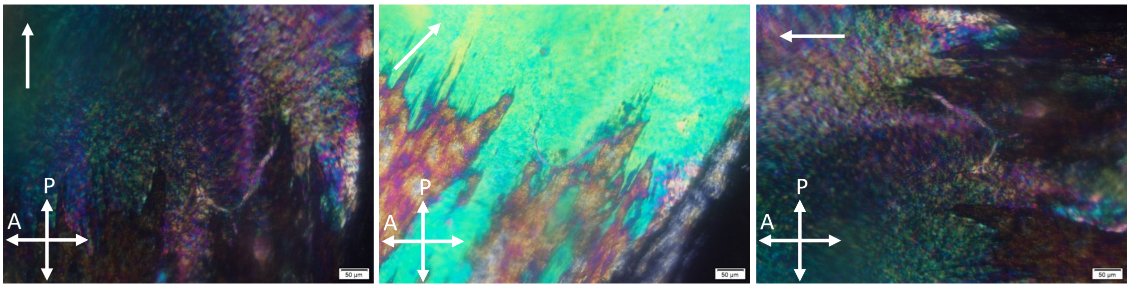

Figure SI16. MOP textures of partially aligned sample of compound **T-2CTPA** between KBr plates. The alignment direction is indicated with an arrow.

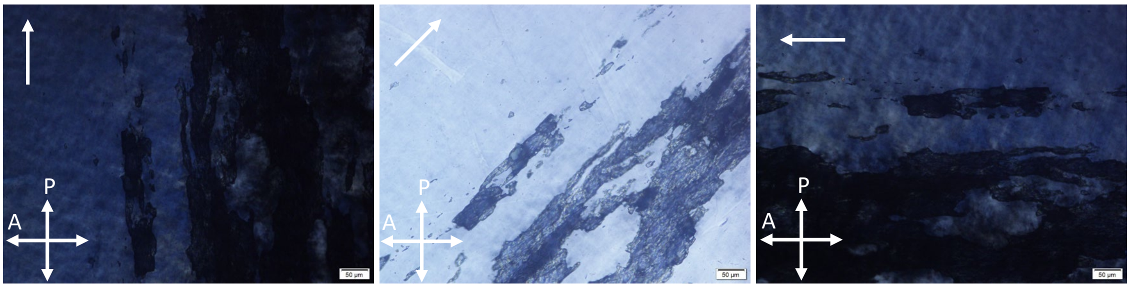

Figure SI17. MOP textures of partially aligned sample of compound **T-3CTPA** between KBr plates. The alignment direction is indicated with an arrow.

CD spectra of T-2C*TPA

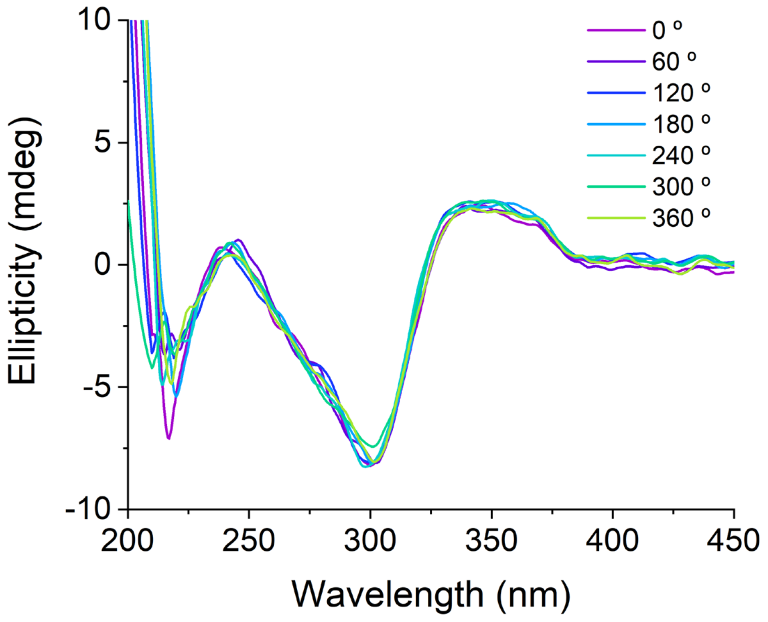

Figure SI18. CD spectra of compound **T-2C*TPA** in the mesophase recorded every 60° rotating the sample around the measurement direction.

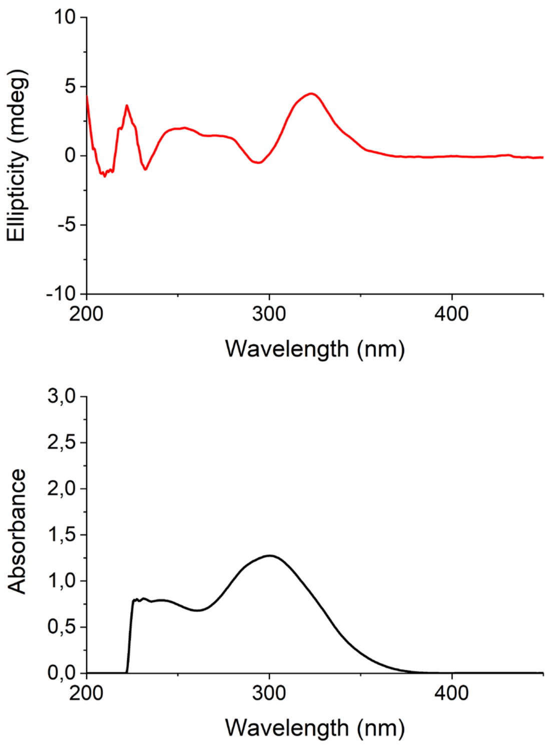

Figure SI19. CD and Uv-vis spectra of compound **T-2C*TPA** in diluted solution (10^{-5} M) in DCM.

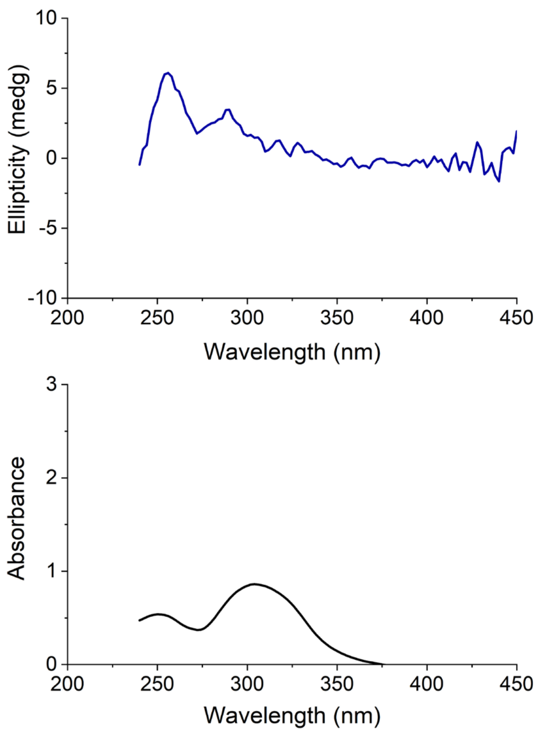

Figure SI20. CD and UV-vis spectra of compound **N_3-2C*TPA** in THF diluted solution ($3{\cdot}10^{-5}$ M).

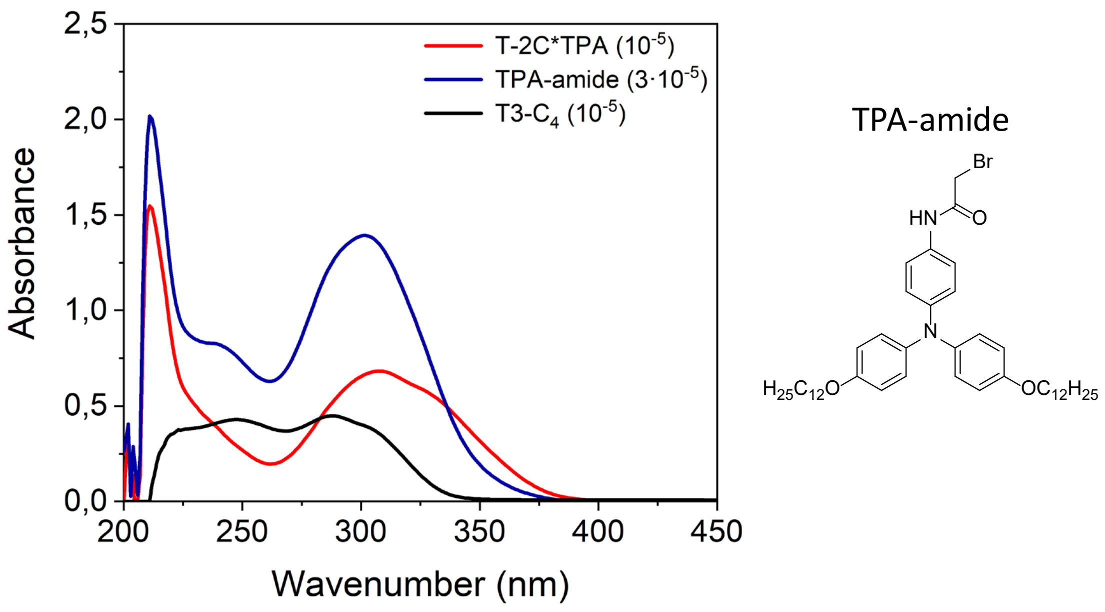

Figure SI21. UV-vis spectrum of compounds **T-2CTPA***, **TPA-amide** and the tristriazolyltriazine derivative[1] core in THF diluted solution.

1. Feringán, B.; Romero, P.; Serrano, J. L.; Giménez, R.; Sierra, T., Supramolecular Columnar Liquid Crystals Formed by Hydrogen Bonding between a Clicked Star-Shaped s-Triazine and Benzoic Acids. *Chem. Eur. J.* **2015,** *21* (24), 8859-8866.

Electrochemical properties

Table SI1. Electrochemical data of the compounds **T-2CTPA**, **T-2C*TPA** and **T-3CTPA** in tetrahydrofuran solution.

**Compound**	**E^{red} (V) *vs* Ag/AgCl**	**$E^{ox}_{1/2}$ (V) *vs* Ag/AgCl**	**E^{red} (V)[a] *vs* FOC**	**$E^{ox}_{1/2}$ (V)[a] *vs* FOC**	**HOMO (eV)[b]**	**LUMO (eV)[c]**
**T-2CTPA**	-1.61	0.78	-2.17	0.22	-5.02	-2.63
**T-2C*TPA**	-1.61	0.78	-2.17	0.22	-5.02	-2.63
**T-3CTPA**	-1.63	0.76	-2.19	0.20	-5.00	-2.61

[a]$E_{1/2}$ = 0.56 V *vs* Ag/AgCl. [b]E_{HOMO} = -e[$E^{ox}_{1/2}$ *vs* FOC + 4.8 V]. [c]E_{LUMO} = -e[E^{red} *vs* FOC + 4.8 V].

Cyclic voltammograms

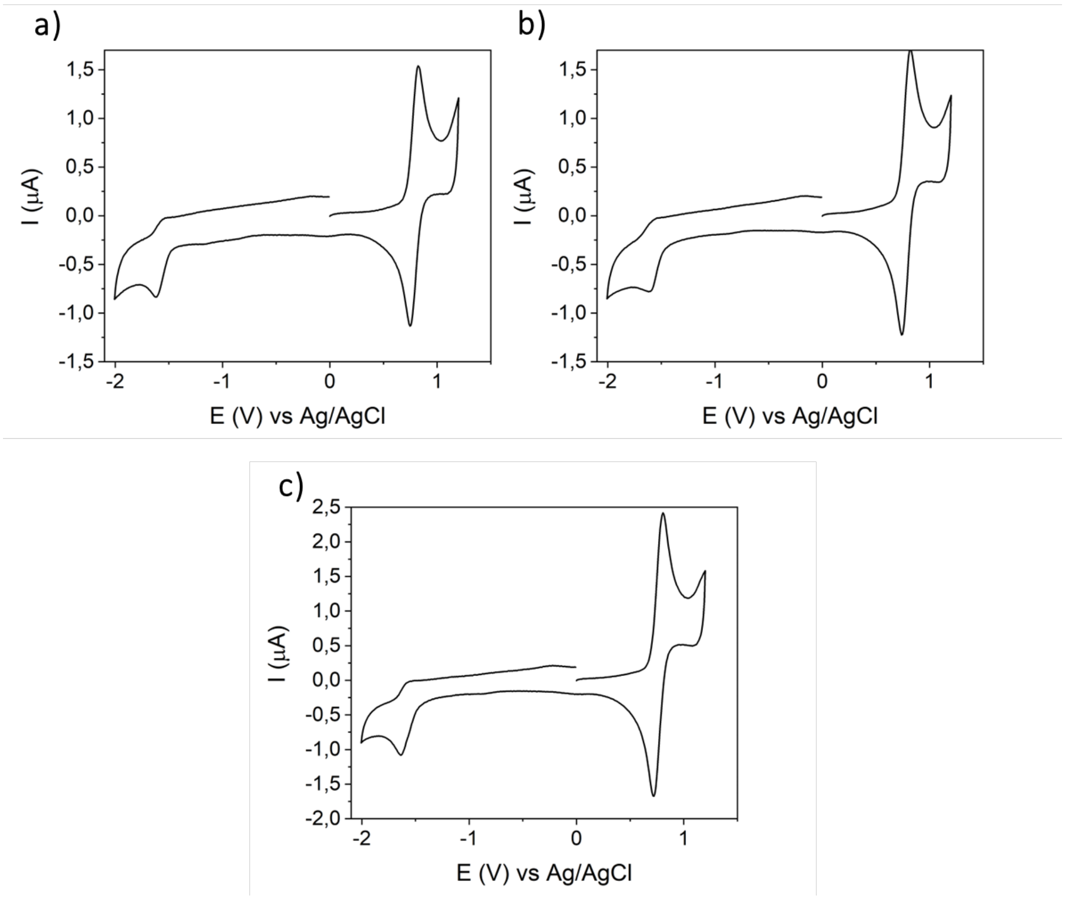

Figure SI22. Cyclic voltammograms in solution of **T-2CTPA** (a), **T-2C*TPA** (c) and **T-3CTPA** (e) in THF $1{\cdot}10^{-4}$ M and 0.1 M of NBu4PF6.

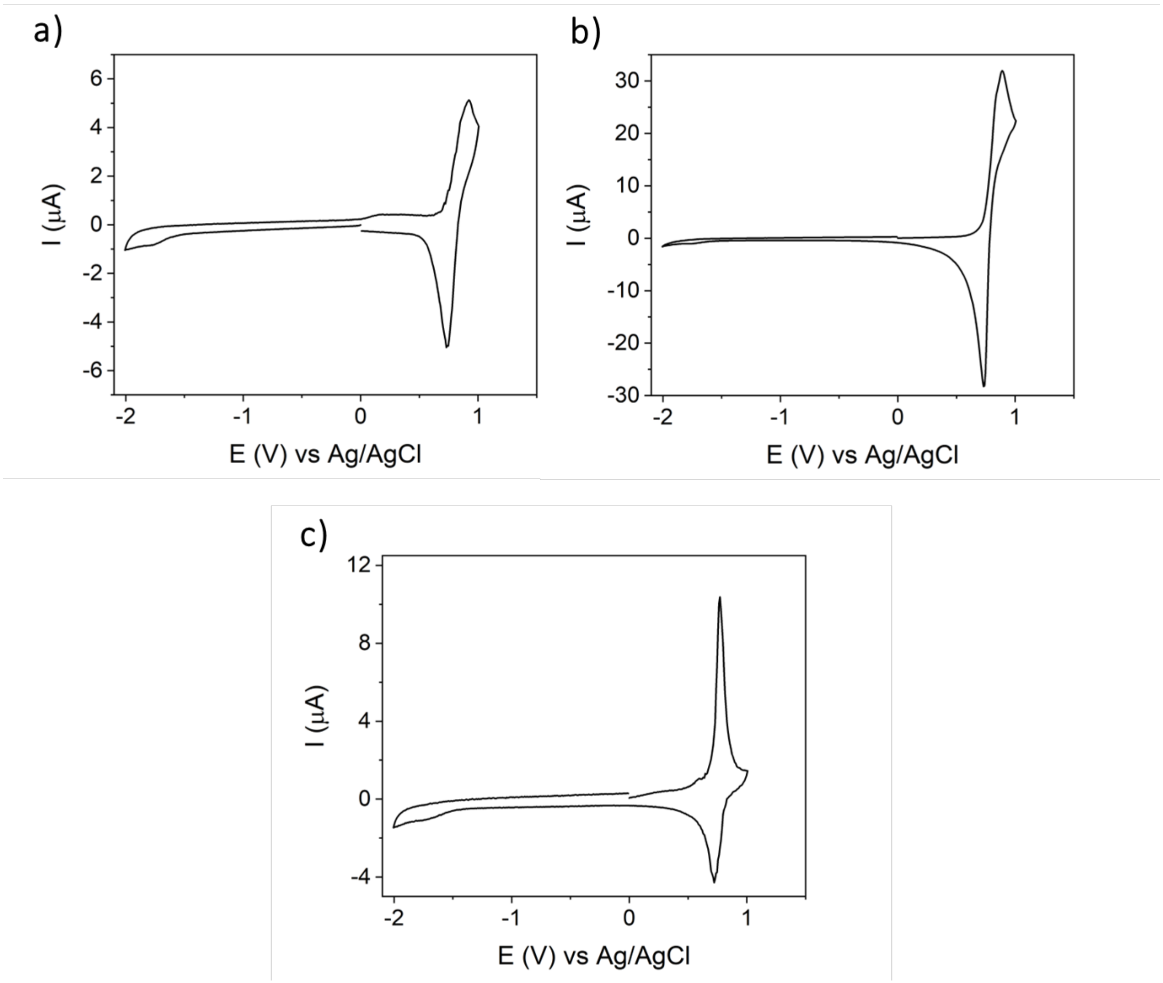

Figure SI23. Cyclic voltammograms in thin film of compounds **T-2CTPA** (b), **T-2C*TPA** (d) and **T-3CTPA** (f) in a 0.1 M NBu4PF6 solution in acetonitrile.

<u>Charge mobility measurements</u>

The charge mobility of the three materials was measured by the Space-Charge Limited Current (SCLC) method in solution processed samples. It entails the acquisition of the electric current flowing through the material, placed between two electrodes, as a function of the applied voltage. For low applied voltages, the current follows Ohm's law, while at higher voltages, if it is limited by a space-charge field, the current dependence on voltage is quadratic, following the Mott-Gurney law, in which the effect of the traps is neglected:

$$J = \frac{9}{8}\varepsilon\varepsilon_0\mu\frac{V^2}{d^3}$$

Where *J* is the current density, ε is the relative dielectric constant of the material, ε_0 is the dielectric constant of the vacuum, *V* is the applied voltage and *d* is the sample thickness.

To obtain a good charge injection between the electrode and the material, the contact between both should be ohmic, i.e. the difference between the HOMO (in the case of p-conductors) or

the LUMO (in the case of n-conductors) level energy of the material and the work function of the injecting electrode must not be higher than 0.3-0.4 eV. Since in the case of these materials the energy of HOMO level is 5.1 eV, Au electrodes (work function 5.0-5.1 eV) were used to obtain ohmic contact in the hole measurements cells. For electron measurements cells, considering the energy LUMO level is 2.7 eV, $CsCO_3$ coated ITO electrodes (work function 2.9 – 3.1 eV) were used to obtain ohmic contact.

Hole mobility devices were prepared by spin-coating 110 µL of a solution of the material in $CHCl_3$ (350 nm thickness, 30 mg/mL at 1500rpm during 1 min) onto a glass covered by 3 ITO stripes. Afterwards, 3 Au stripes (100 nm thickness) orthogonal to ITO stripes were deposited on top of the material layer by evaporation under vacuum. In this way, 9 independent 0.6 mm^2 areas between both electrodes are obtained.

Electron mobility devices were prepared by spin-coating a solution of $CsCO_3$ in 2 methoxy ethanol (3 mg/ml, 4500 rpm for 60 sec) onto a glass covered by 3 ITO stripes and the $CsCO_3$ layer was annealed at 120 ºC for 10 min. After that, a material layer was placed by spin-coating 110 µL of a solution of the material in $CHCl_3$ (350 nm thickness, 30 mg/mL at 1500rpm during 1 min). On top of it, 3 Al stripes (100 nm thickness) orthogonal to ITO stripes were deposited on top of the material layer by evaporation under vacuum. In this way, 9 independent 0.6 mm^2 areas between both electrodes are obtained.

In both types of devices, the samples were annealed below the clearing point in order to favor the correct mesophase formation and orientation. The compound **T-2C*TPA** was annealed at 140 °C during four hours, and compounds **T-2CTPA** and **T-3CTPA** were annealed at 200 °C for one hour to avoid decomposition. Representative POM pictures of hole mobility samples from compounds **T-2CTPA** and **T-3CTPA** shown in figures SI24.

I/V measurement was carried out using an electrometer, either a Keithley 6517A or a Keithley 2636B. An HP 4284A Precision LCR Meter was used to measure the capacity of the cells and obtain the dielectric constant.

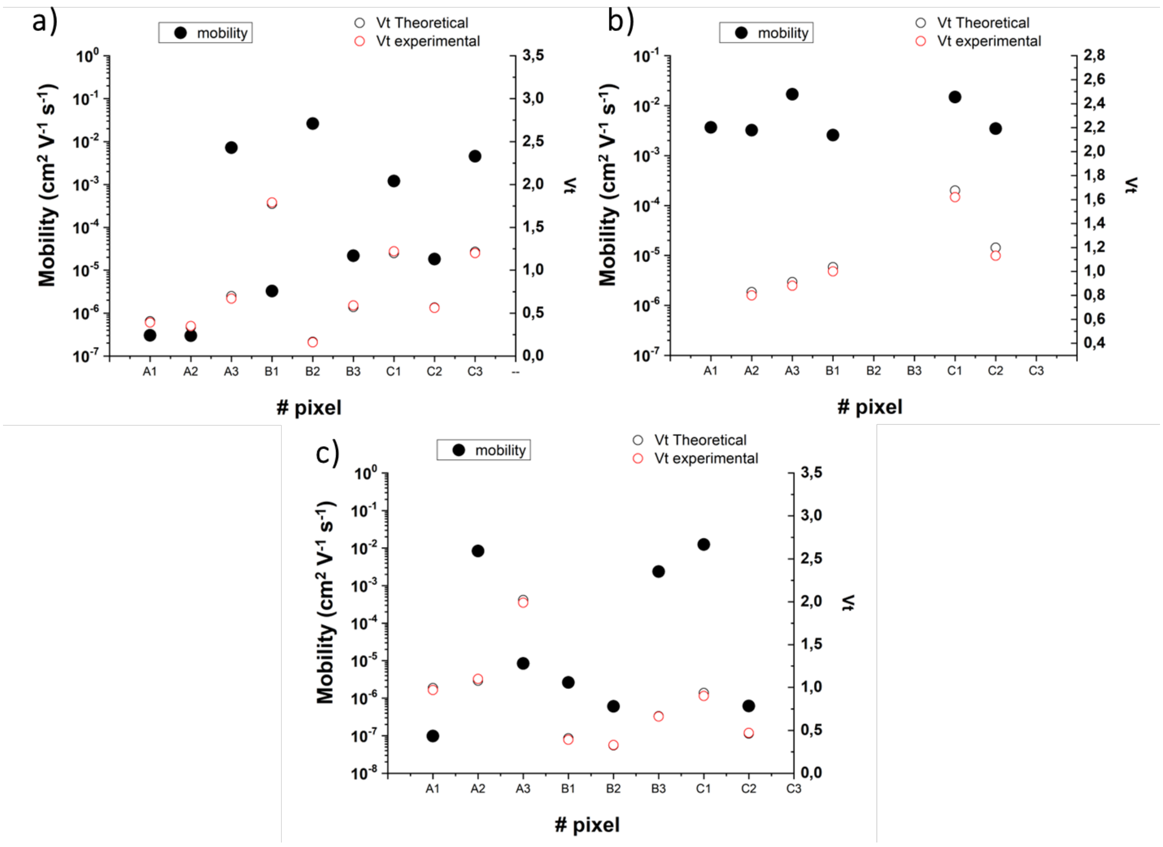

Figure SI24. Hole mobility measurements of compounds (a) **T-2CTPA**, (b) **T-2C*TPA** and (c) **T-3CTPA** in different areas (labelled A1, A2, *etc.*) after thermal treatment. In areas without measurements high currents were obtained but the SCLC regime was not reached.

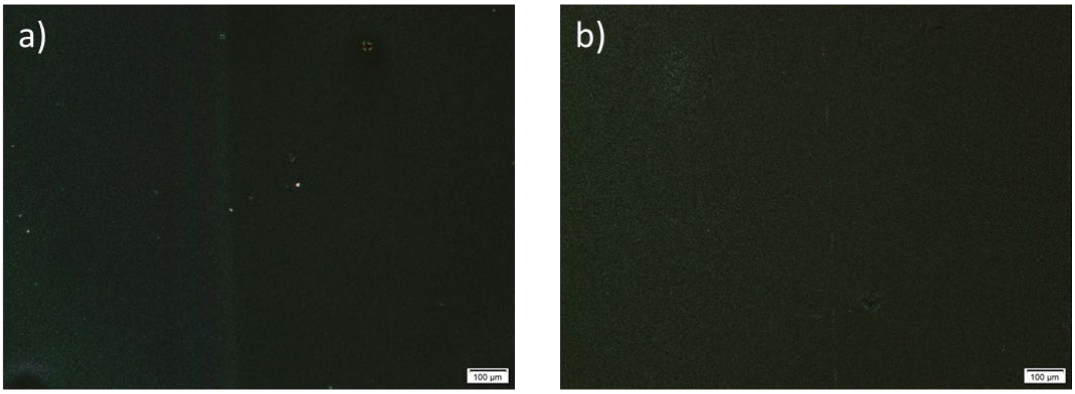

Figure SI25. Photomicrographs of textures observed by POM for (a) **T-2CTPA** and (b) **T-3CTPA** at room temperature after treated at 200 °C during 1h.

Concentration dependent NMR experiments

a)

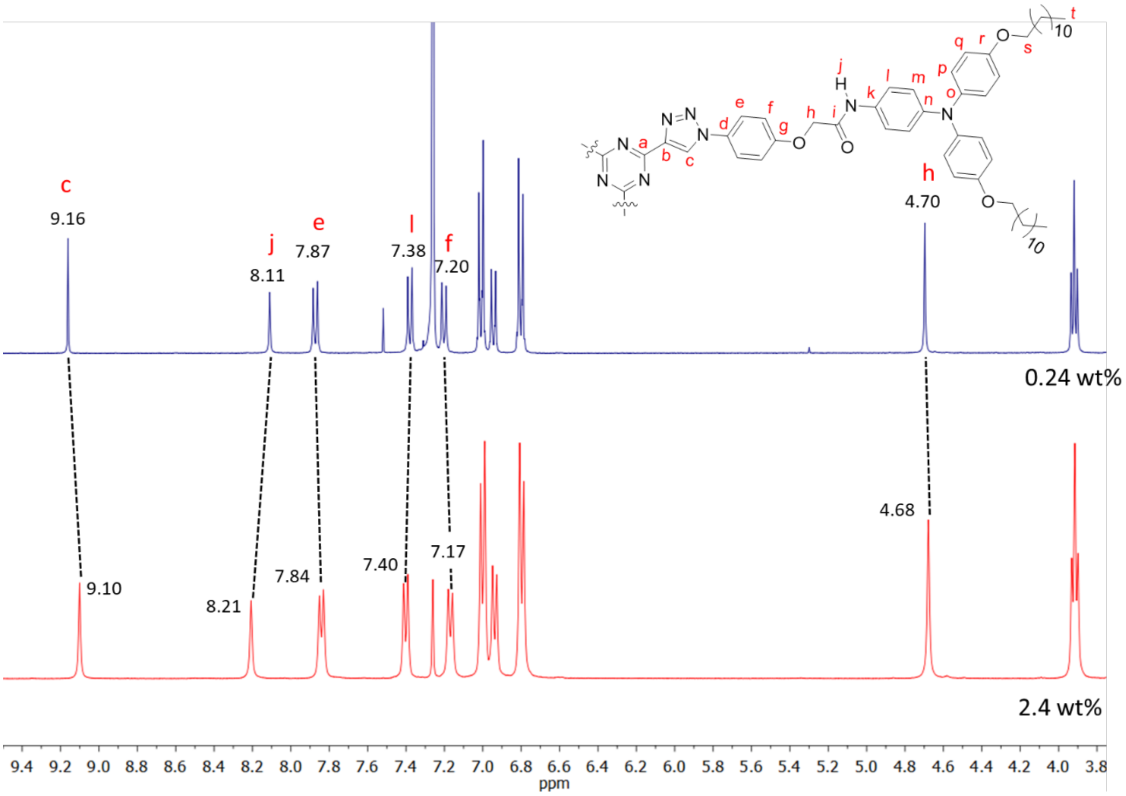

b)

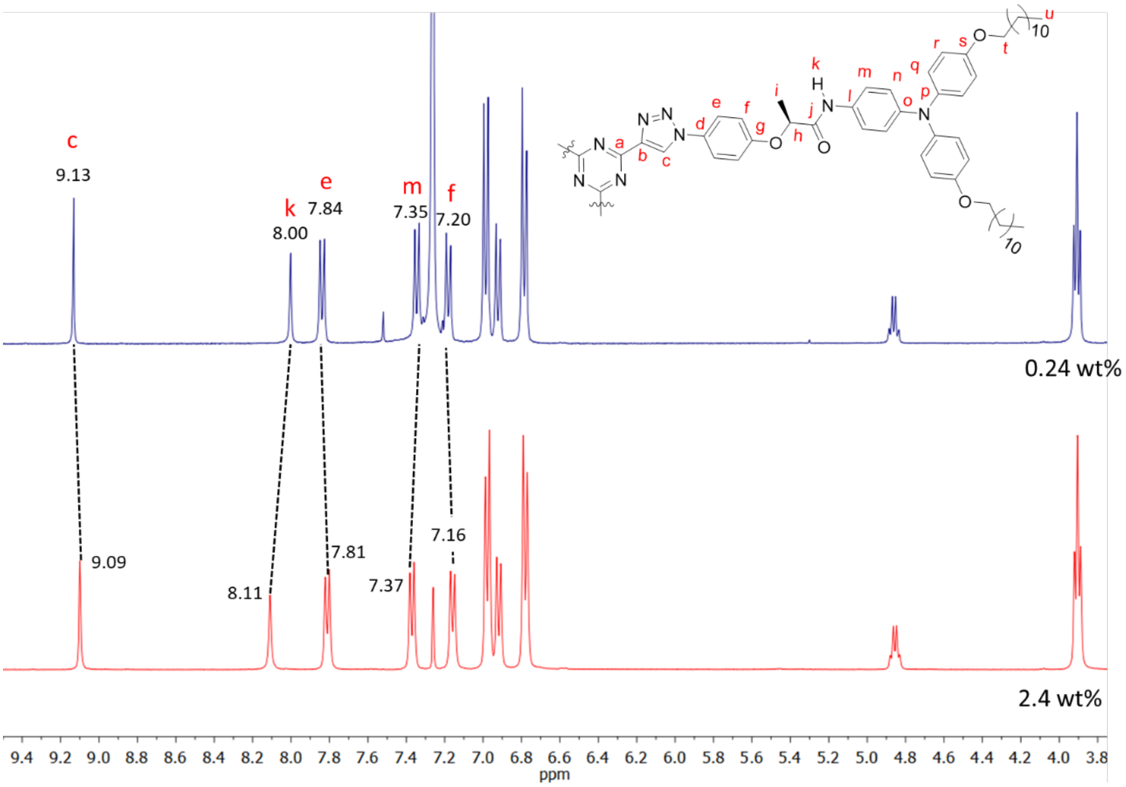

c)

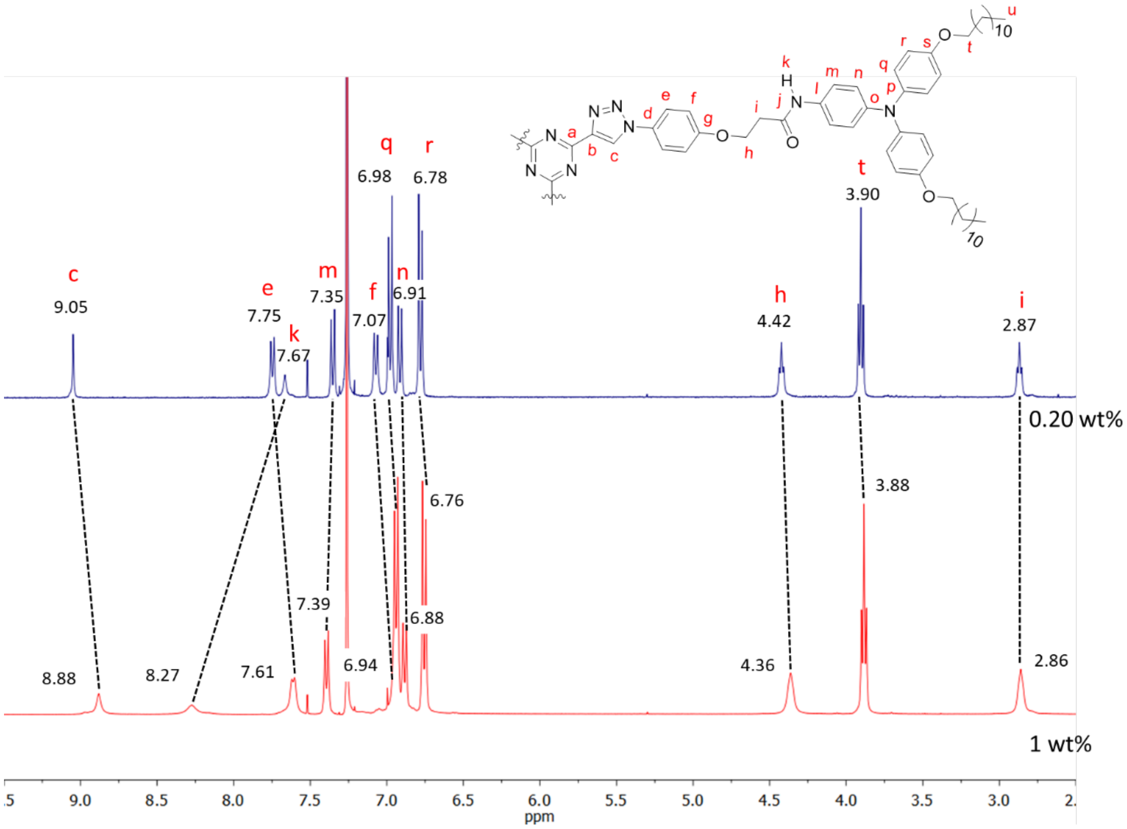

Figure SI26. NMR spectra of (a) **T-2CTPA**, (b) **T-2C*TPA** and (c) **T-3CTPA** in $CDCl_3$ recorded at two concentrations and indicating the shift of the most significant peaks.
